# Supplementary figures and images for: Tissue-specific mitochondrial HIGD1C promotes oxygen sensitivity in carotid body chemoreceptors
Source: eLife. 2022 Oct 18;11:e78915. doi: 10.7554/eLife.78915 (PMC9635879; doi:10.7554/eLife.78915)

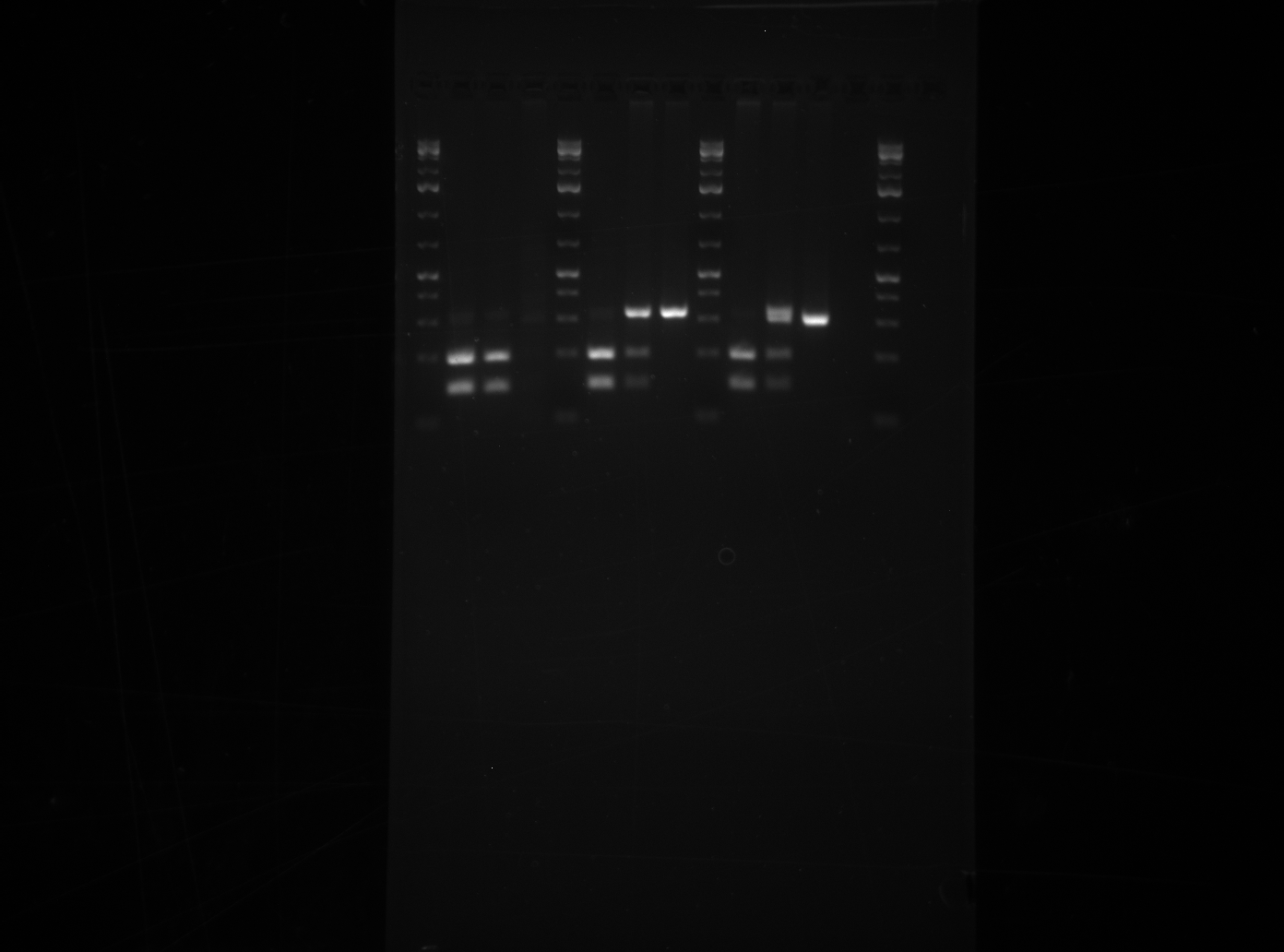

Supplement: Figure 1—figure supplement 2—source data 1. [file elife-78915-fig1-figsupp2-data1.zip › Fig 1-figure supplement 2-source data 1/Fig1-S2C-right-P9P8.tiff]

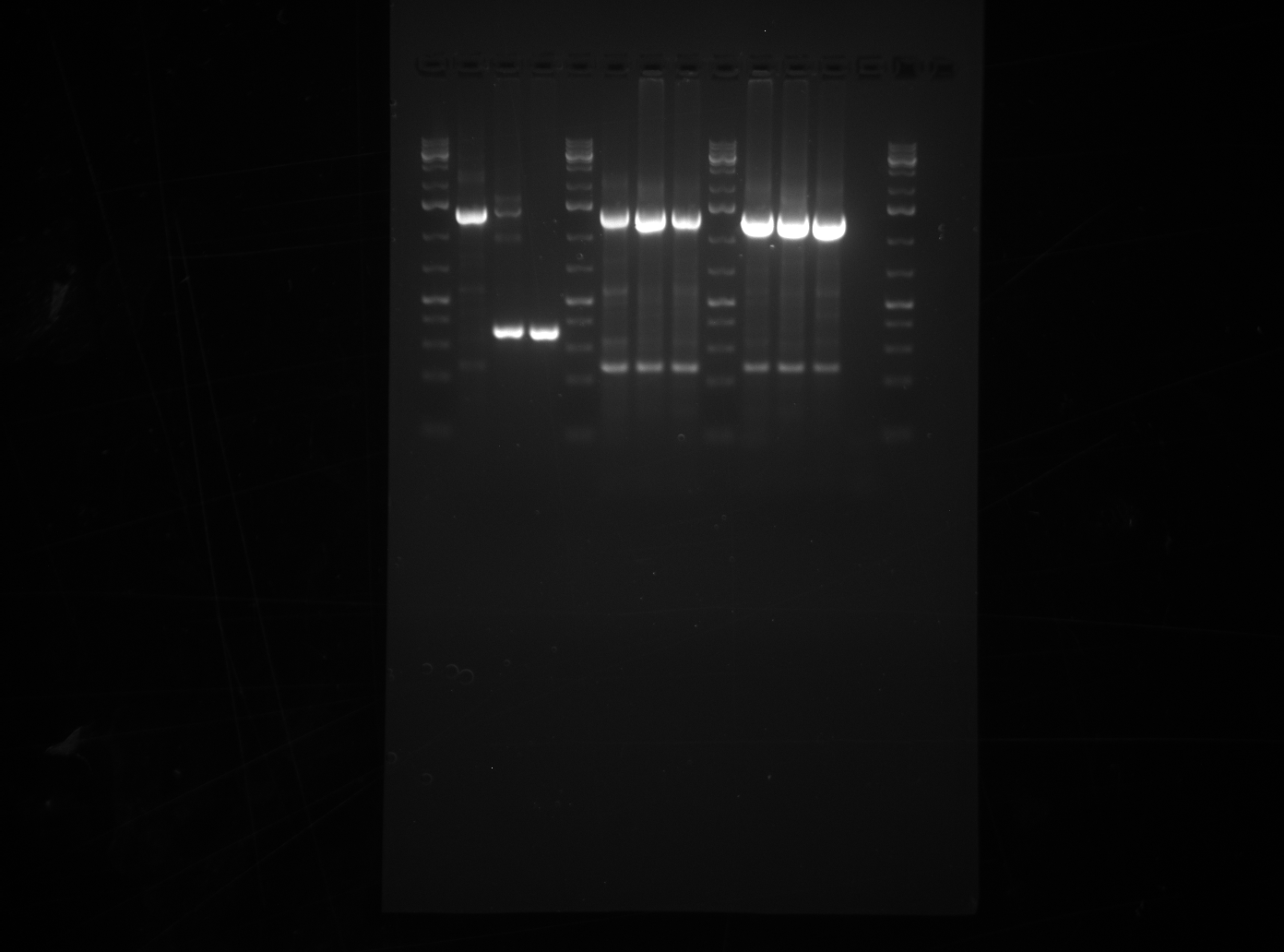

Supplement: Figure 1—figure supplement 2—source data 1. [file elife-78915-fig1-figsupp2-data1.zip › Fig 1-figure supplement 2-source data 1/Fig1-S2C-left-P9P11.tiff]

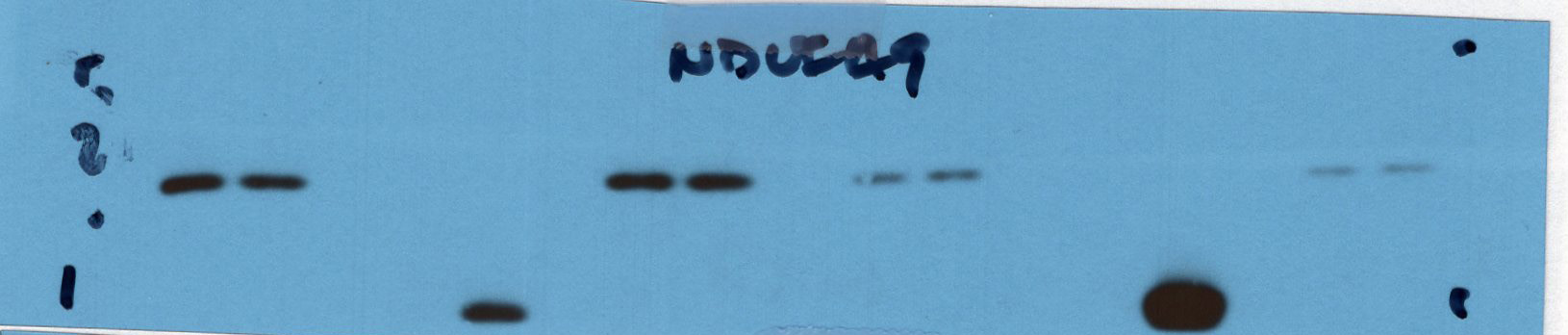

Supplement: Figure 5—source data 1. [file elife-78915-fig5-data1.zip › Fig 5-source data 1/Fig5B_ndufa9.jpg]

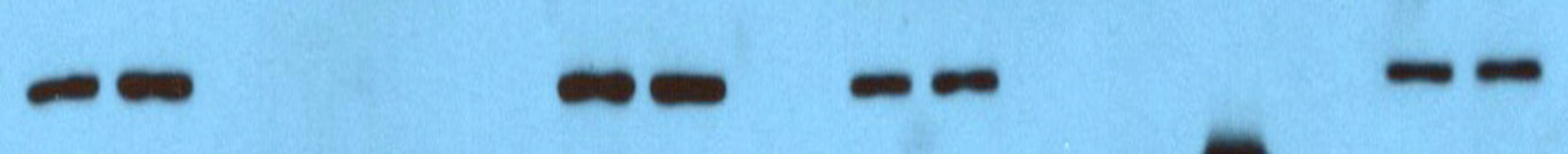

Supplement: Figure 5—source data 1. [file elife-78915-fig5-data1.zip › Fig 5-source data 1/Fig5B_sdha.jpg]

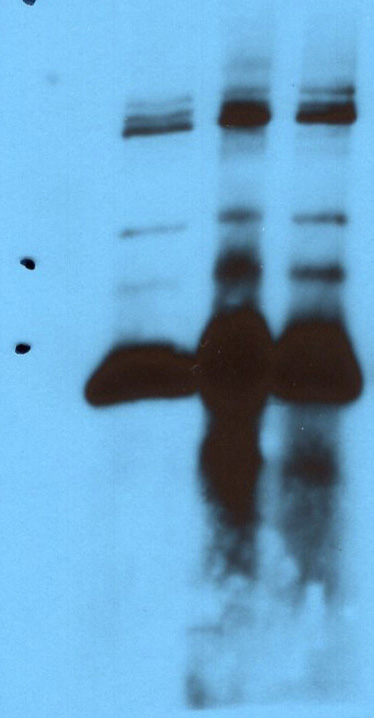

Supplement: Figure 5—source data 1. [file elife-78915-fig5-data1.zip › Fig 5-source data 1/Fig5A_cox5b.jpg]

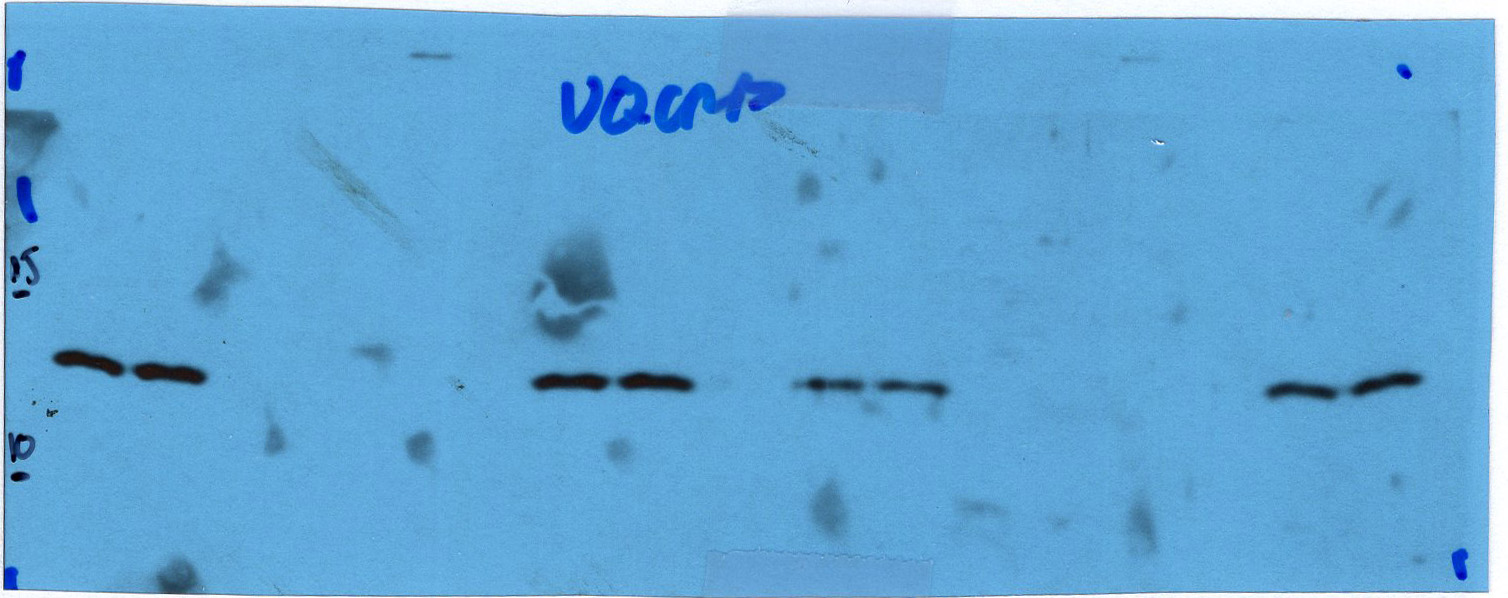

Supplement: Figure 5—source data 1. [file elife-78915-fig5-data1.zip › Fig 5-source data 1/Fig5B_uqcrb.jpg]

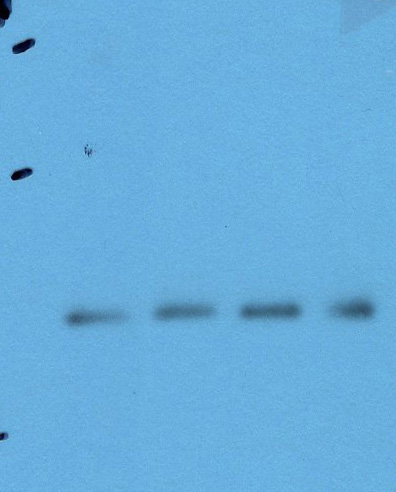

Supplement: Figure 5—source data 1. [file elife-78915-fig5-data1.zip › Fig 5-source data 1/Fig5C_sdhaDDM.jpg]

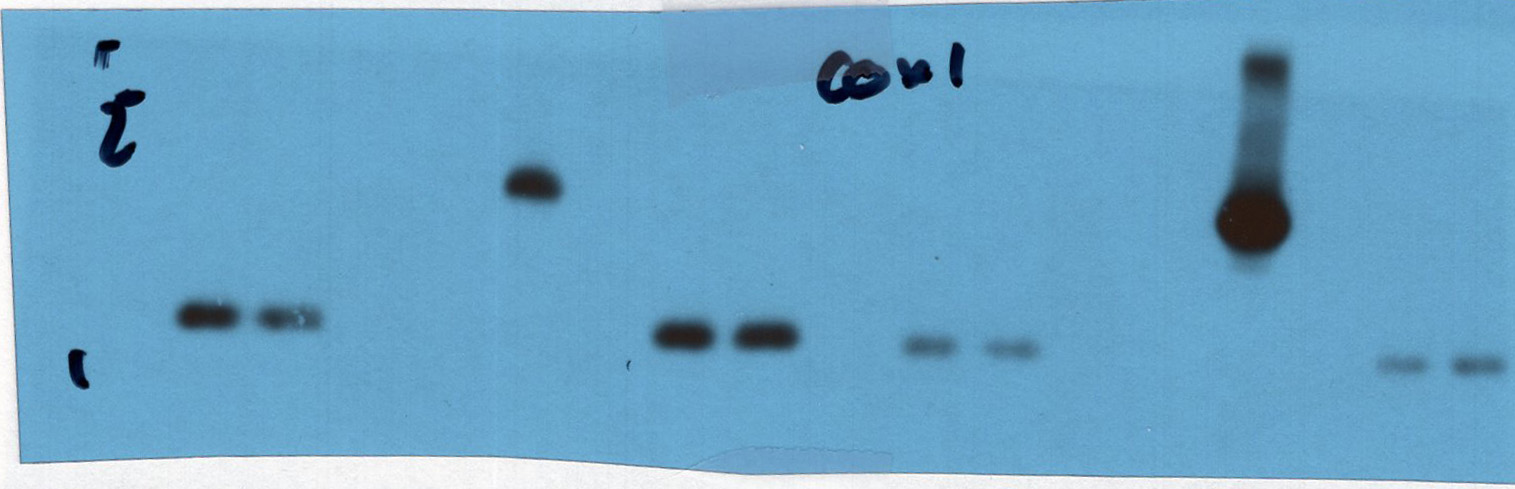

Supplement: Figure 5—source data 1. [file elife-78915-fig5-data1.zip › Fig 5-source data 1/Fig5B_cox1.jpg]

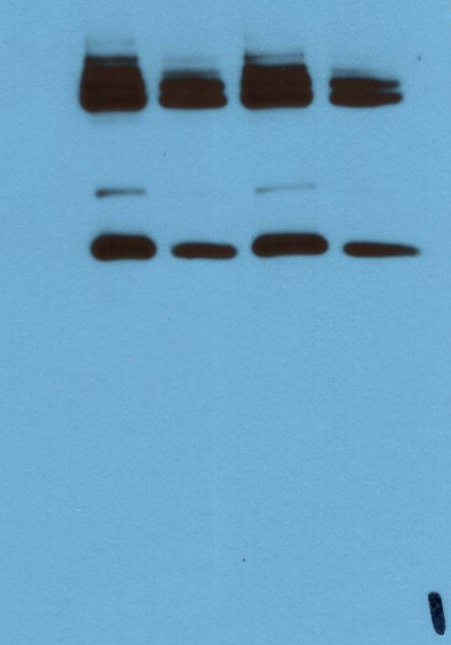

Supplement: Figure 5—source data 1. [file elife-78915-fig5-data1.zip › Fig 5-source data 1/Fig5C_core2DIG.jpg]

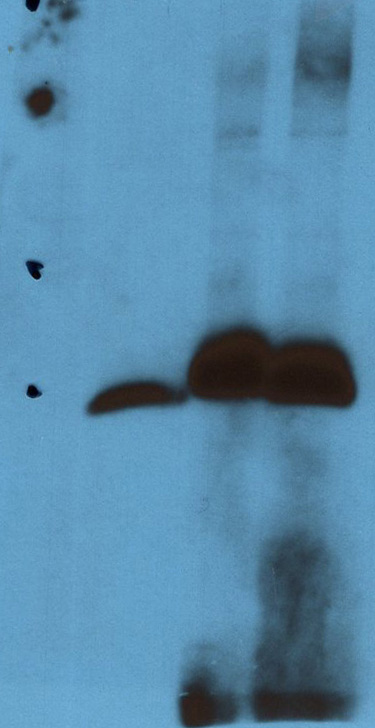

Supplement: Figure 5—source data 1. [file elife-78915-fig5-data1.zip › Fig 5-source data 1/Fig5A_flag.jpg]

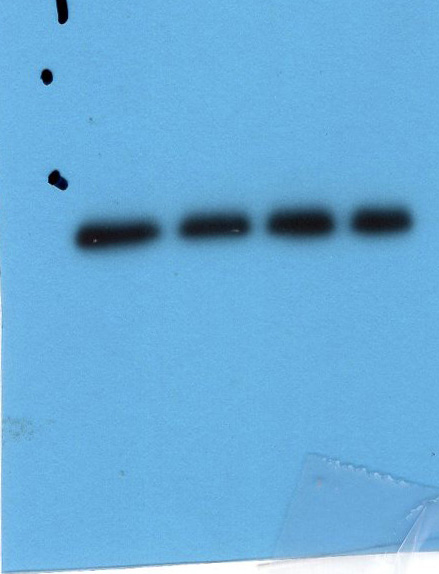

Supplement: Figure 5—source data 1. [file elife-78915-fig5-data1.zip › Fig 5-source data 1/Fig5C_cox1DDM.jpg]

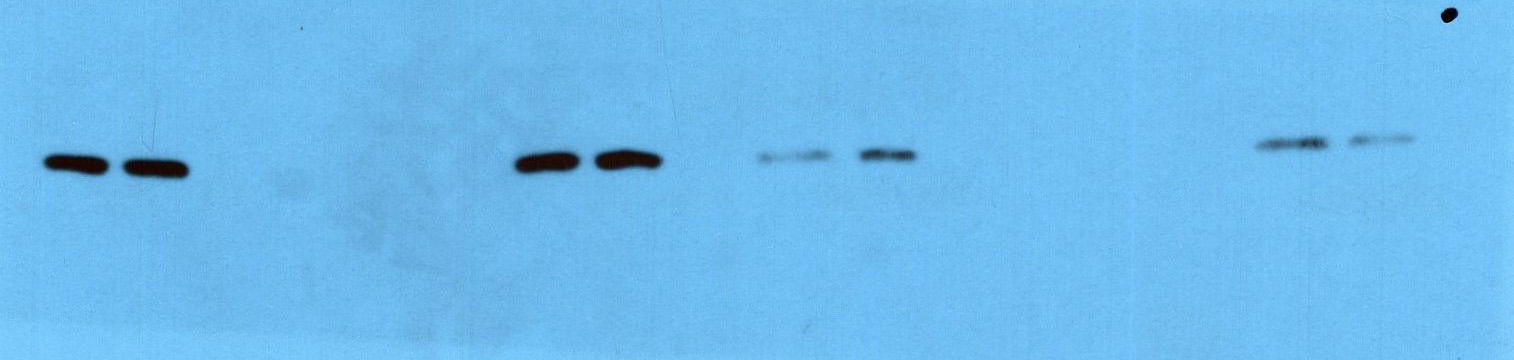

Supplement: Figure 5—source data 1. [file elife-78915-fig5-data1.zip › Fig 5-source data 1/Fig5B_cox4i1.jpg]

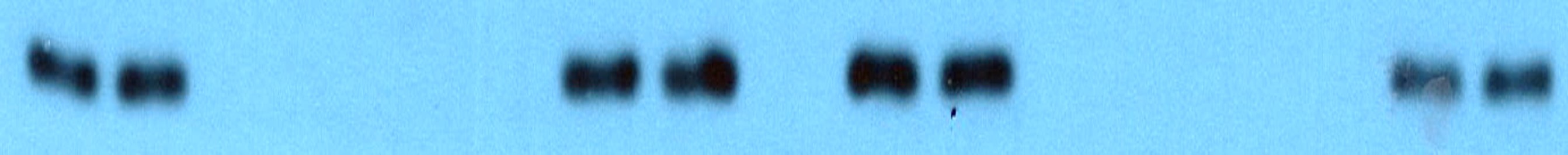

Supplement: Figure 5—source data 1. [file elife-78915-fig5-data1.zip › Fig 5-source data 1/Fig5B_core2.jpg]

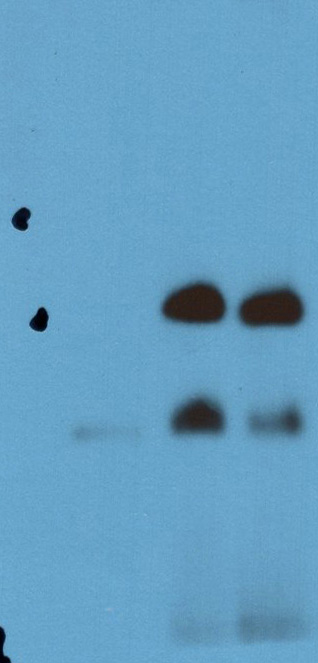

Supplement: Figure 5—source data 1. [file elife-78915-fig5-data1.zip › Fig 5-source data 1/Fig5A_sdha.jpg]

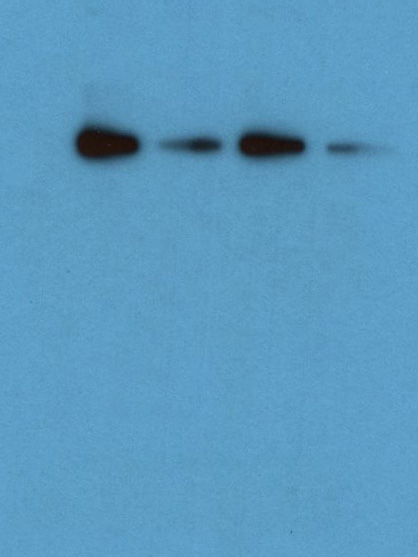

Supplement: Figure 5—source data 1. [file elife-78915-fig5-data1.zip › Fig 5-source data 1/Fig5C_core2DDM.jpg]

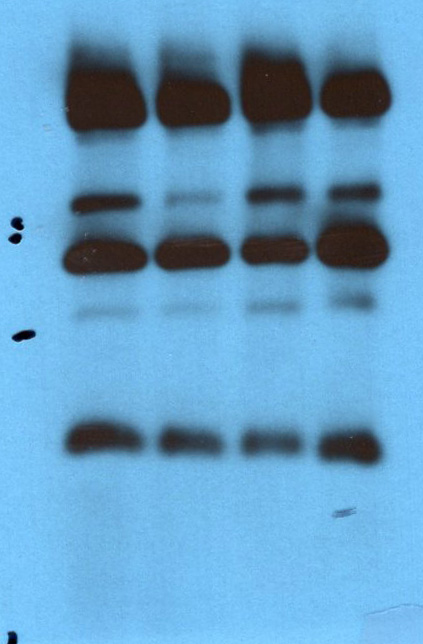

Supplement: Figure 5—source data 1. [file elife-78915-fig5-data1.zip › Fig 5-source data 1/Fig5C_sdhaDIG.jpg]

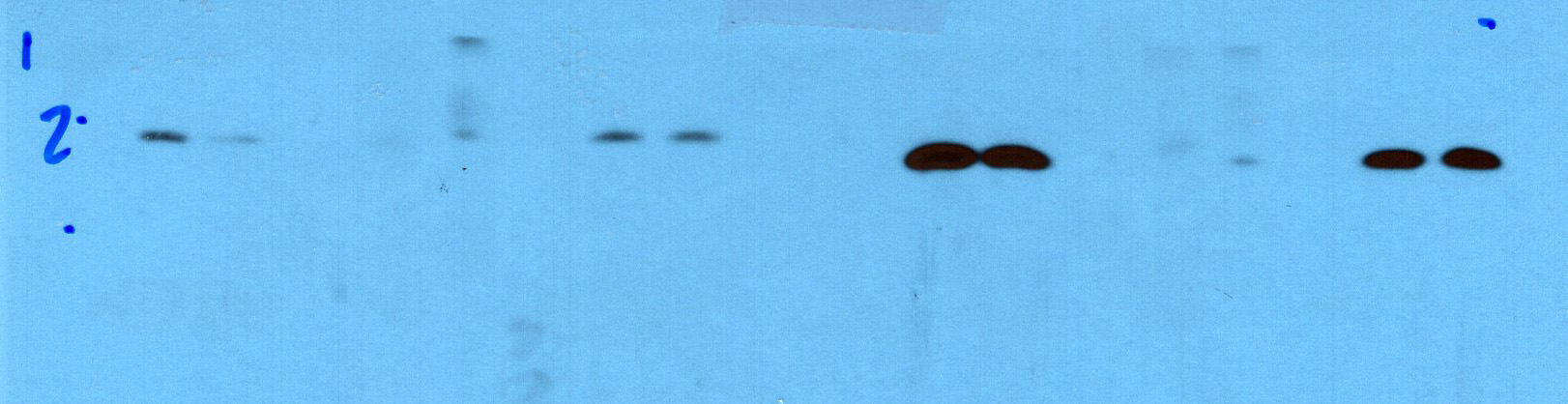

Supplement: Figure 5—source data 1. [file elife-78915-fig5-data1.zip › Fig 5-source data 1/Fig5B_cytc.jpg]

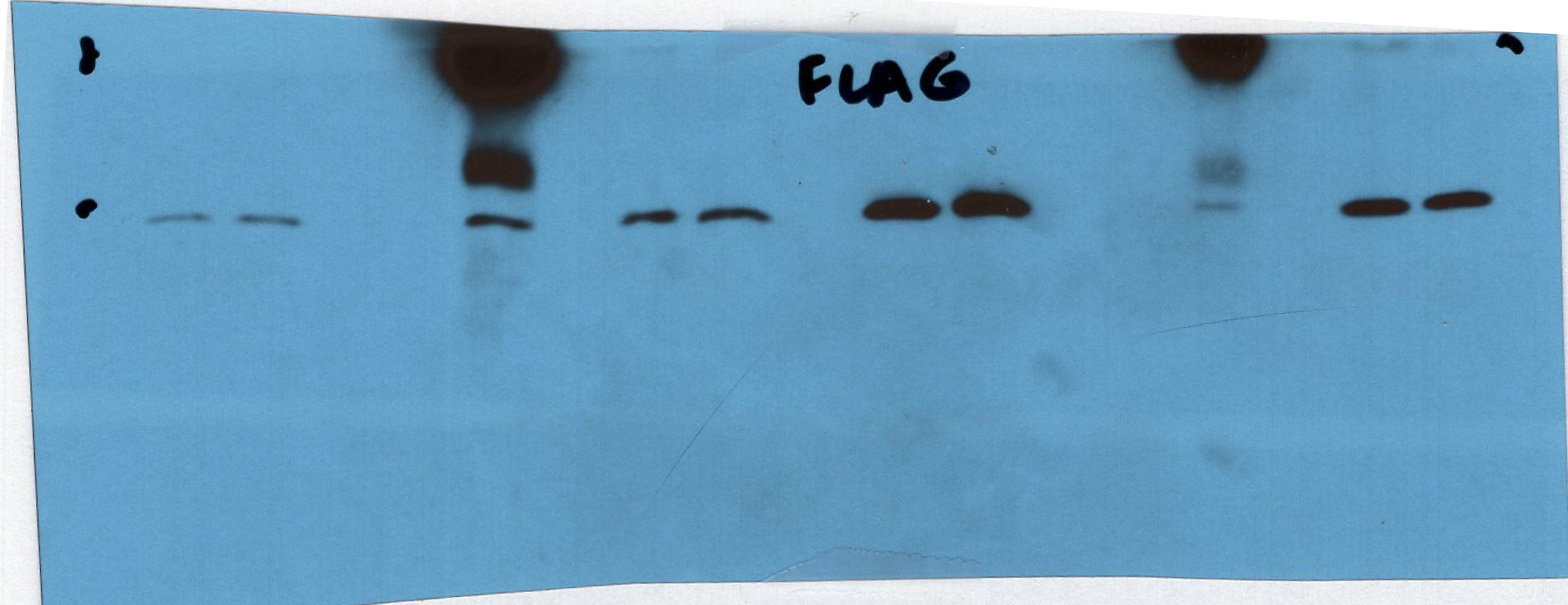

Supplement: Figure 5—source data 1. [file elife-78915-fig5-data1.zip › Fig 5-source data 1/Fig5B_flag.jpg]

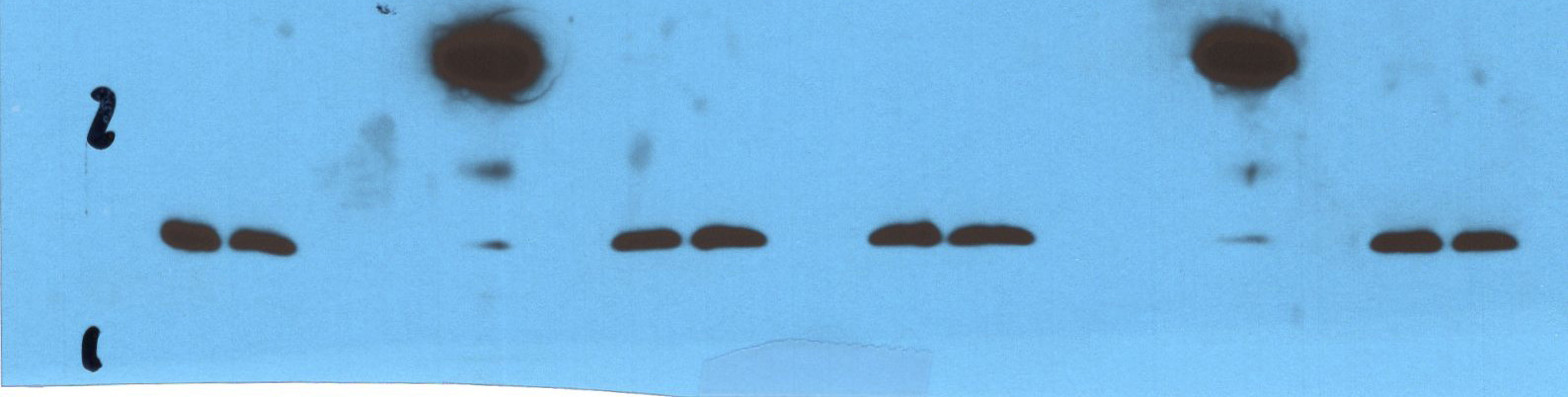

Supplement: Figure 5—source data 1. [file elife-78915-fig5-data1.zip › Fig 5-source data 1/Fig5B_cox5b.jpg]

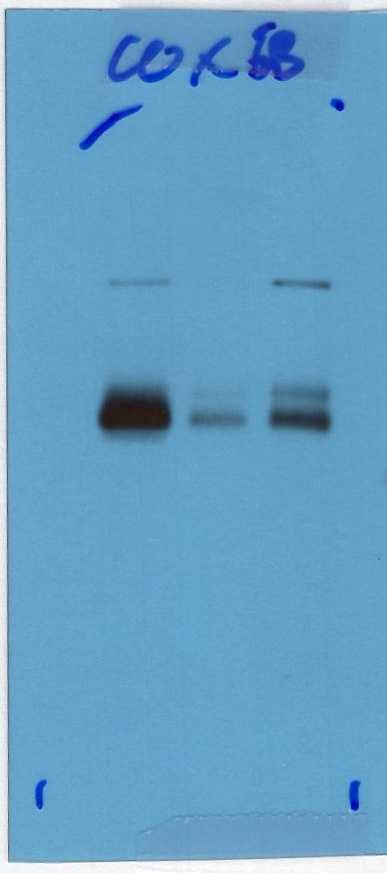

Supplement: Figure 5—figure supplement 1—source data 1. [file elife-78915-fig5-figsupp1-data1.zip › Fig 5-figure supplement 1-source data 1/Fig5s1D_cox5b.jpg]

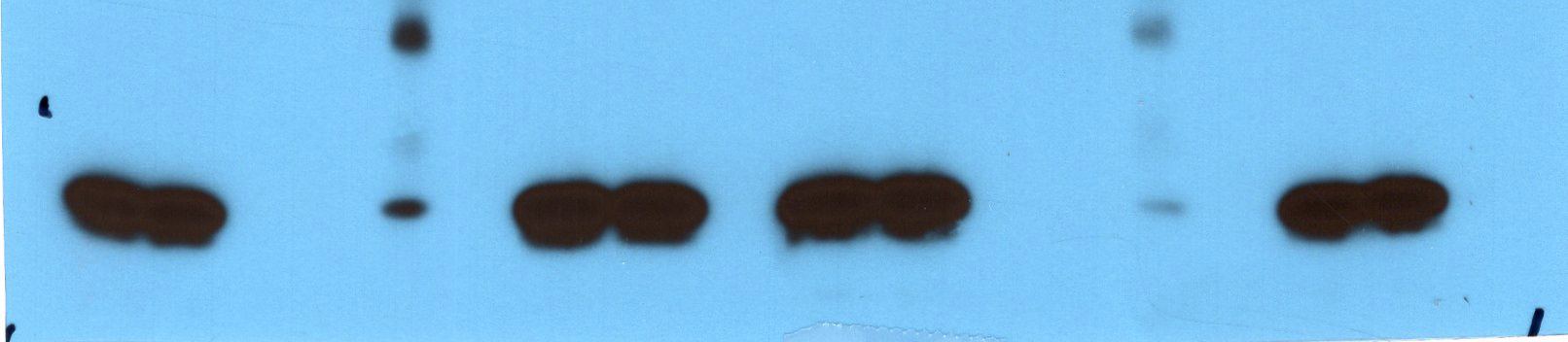

Supplement: Figure 5—figure supplement 1—source data 1. [file elife-78915-fig5-figsupp1-data1.zip › Fig 5-figure supplement 1-source data 1/Fig5s1C_cytc.jpg]

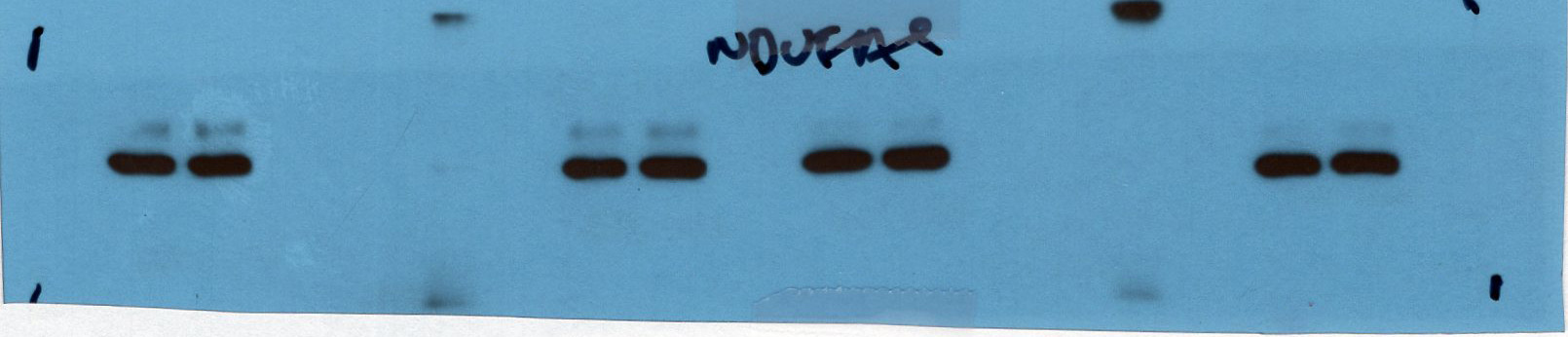

Supplement: Figure 5—figure supplement 1—source data 1. [file elife-78915-fig5-figsupp1-data1.zip › Fig 5-figure supplement 1-source data 1/Fig5s1C_ndufa9.jpg]

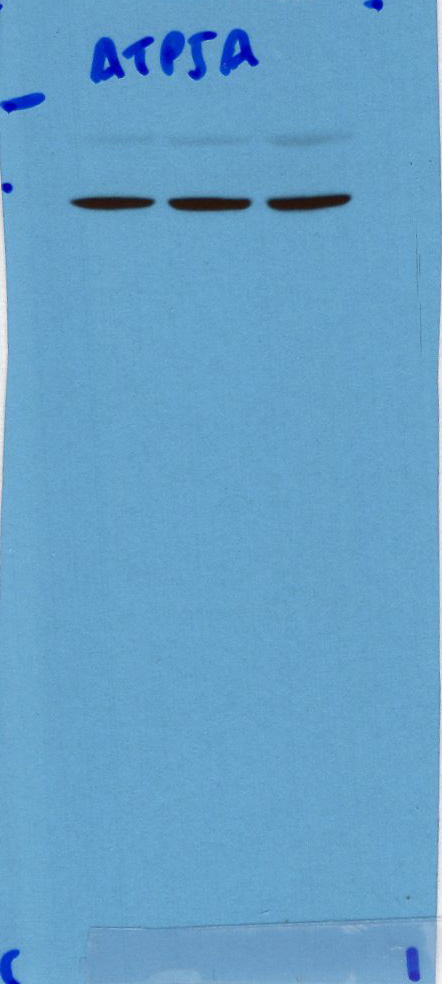

Supplement: Figure 5—figure supplement 1—source data 1. [file elife-78915-fig5-figsupp1-data1.zip › Fig 5-figure supplement 1-source data 1/Fig5s1B_atp5a.jpg]

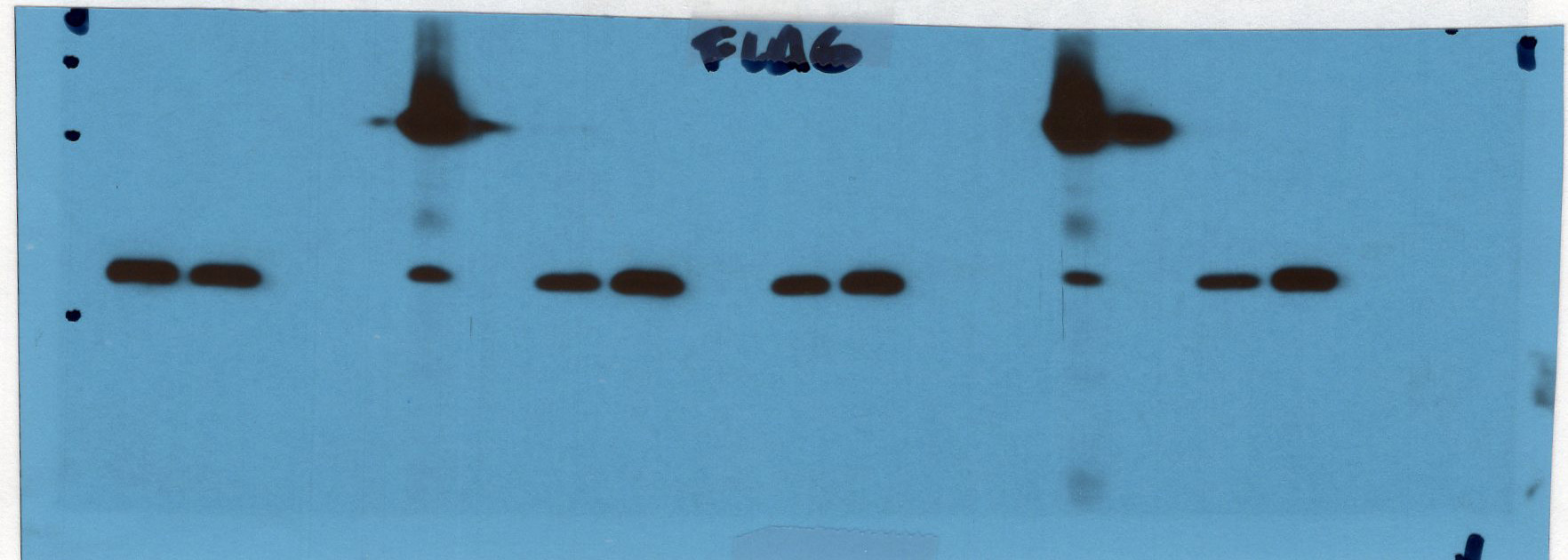

Supplement: Figure 5—figure supplement 1—source data 1. [file elife-78915-fig5-figsupp1-data1.zip › Fig 5-figure supplement 1-source data 1/Fig5s1C_flag.jpg]

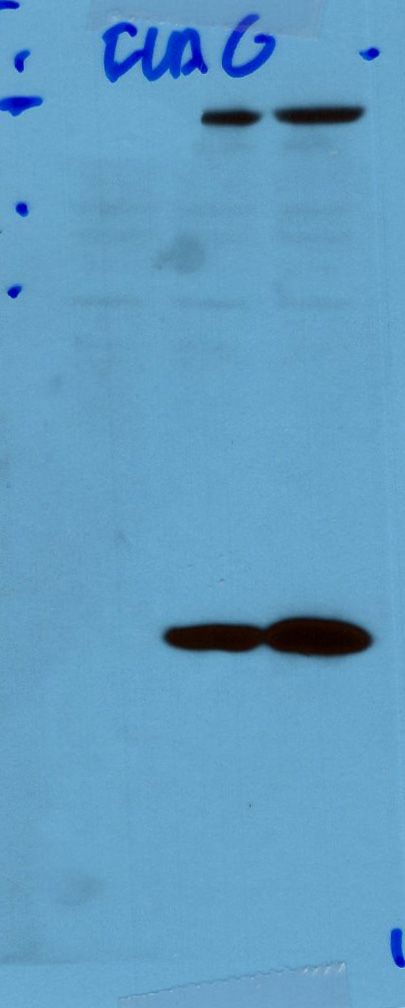

Supplement: Figure 5—figure supplement 1—source data 1. [file elife-78915-fig5-figsupp1-data1.zip › Fig 5-figure supplement 1-source data 1/Fig5s1B_flag.jpg]

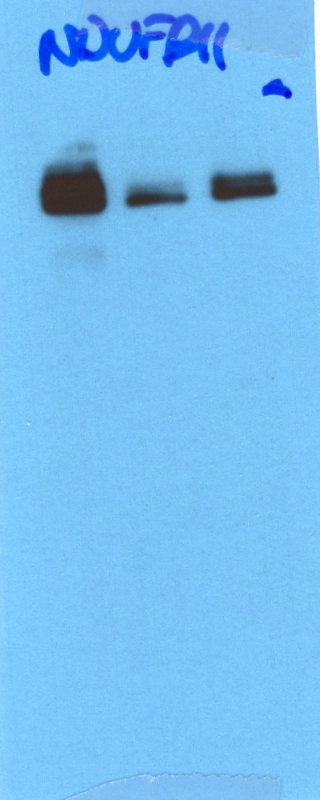

Supplement: Figure 5—figure supplement 1—source data 1. [file elife-78915-fig5-figsupp1-data1.zip › Fig 5-figure supplement 1-source data 1/Fig5s1E_ndufb11.jpg]

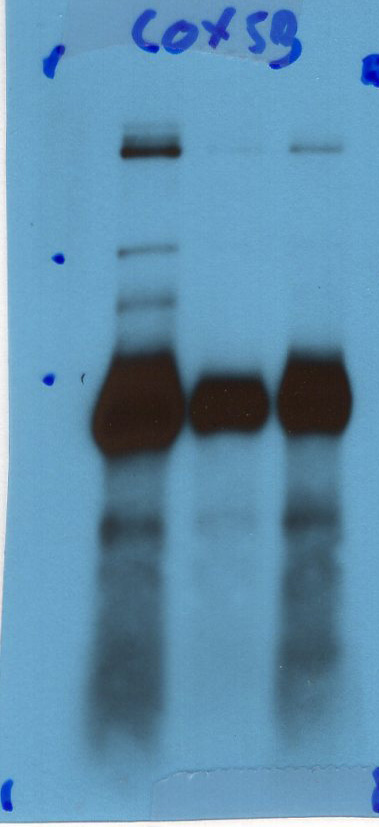

Supplement: Figure 5—figure supplement 1—source data 1. [file elife-78915-fig5-figsupp1-data1.zip › Fig 5-figure supplement 1-source data 1/Fig5s1E_cox5b.jpg]

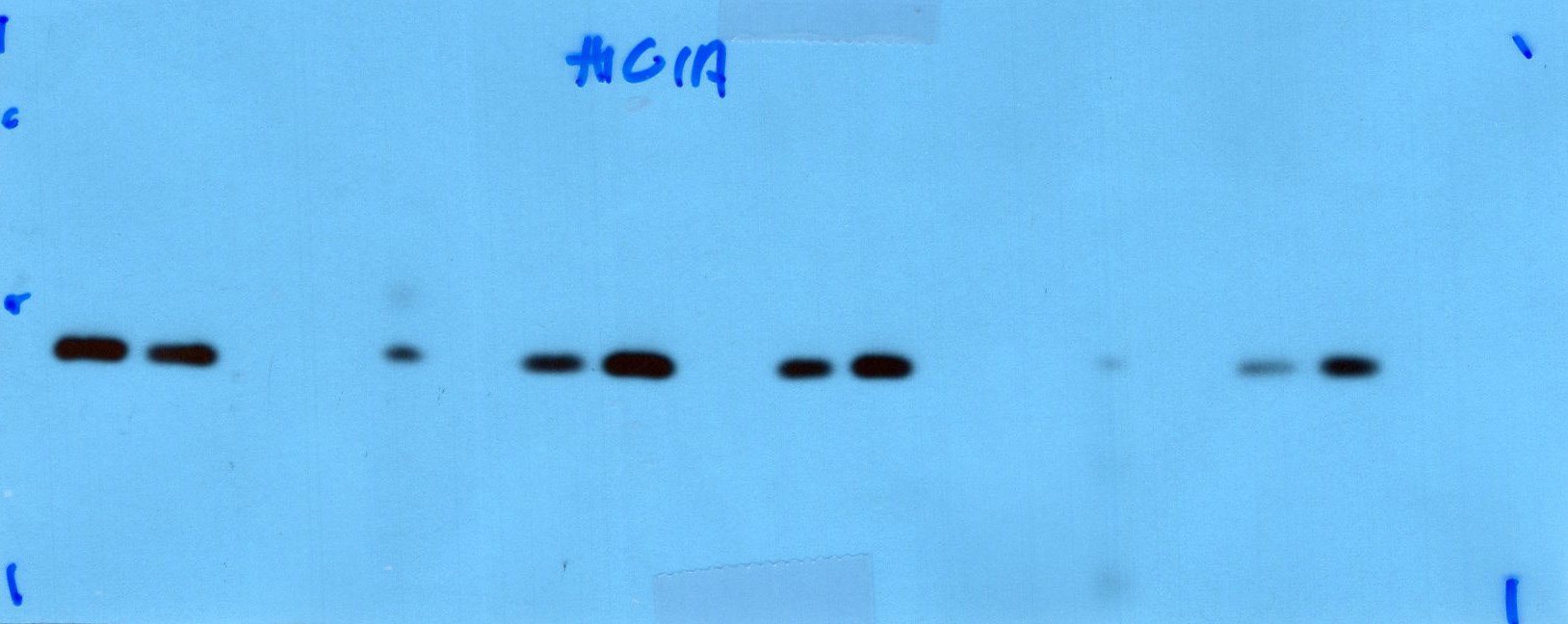

Supplement: Figure 5—figure supplement 1—source data 1. [file elife-78915-fig5-figsupp1-data1.zip › Fig 5-figure supplement 1-source data 1/Fig5s1C_higd1a.jpg]

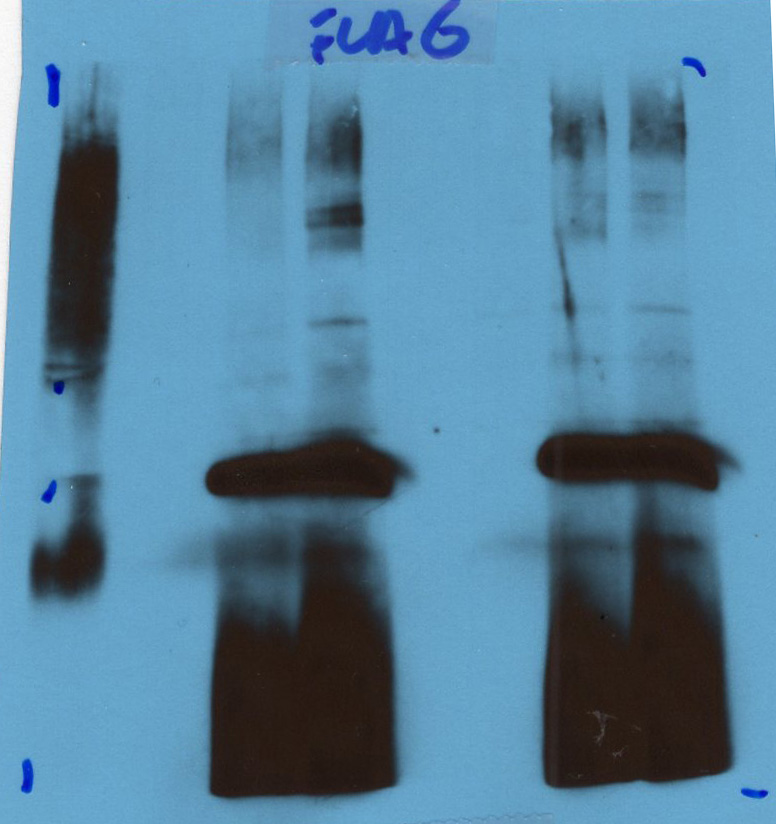

Supplement: Figure 5—figure supplement 1—source data 1. [file elife-78915-fig5-figsupp1-data1.zip › Fig 5-figure supplement 1-source data 1/Fig5s1E_flag.jpg]

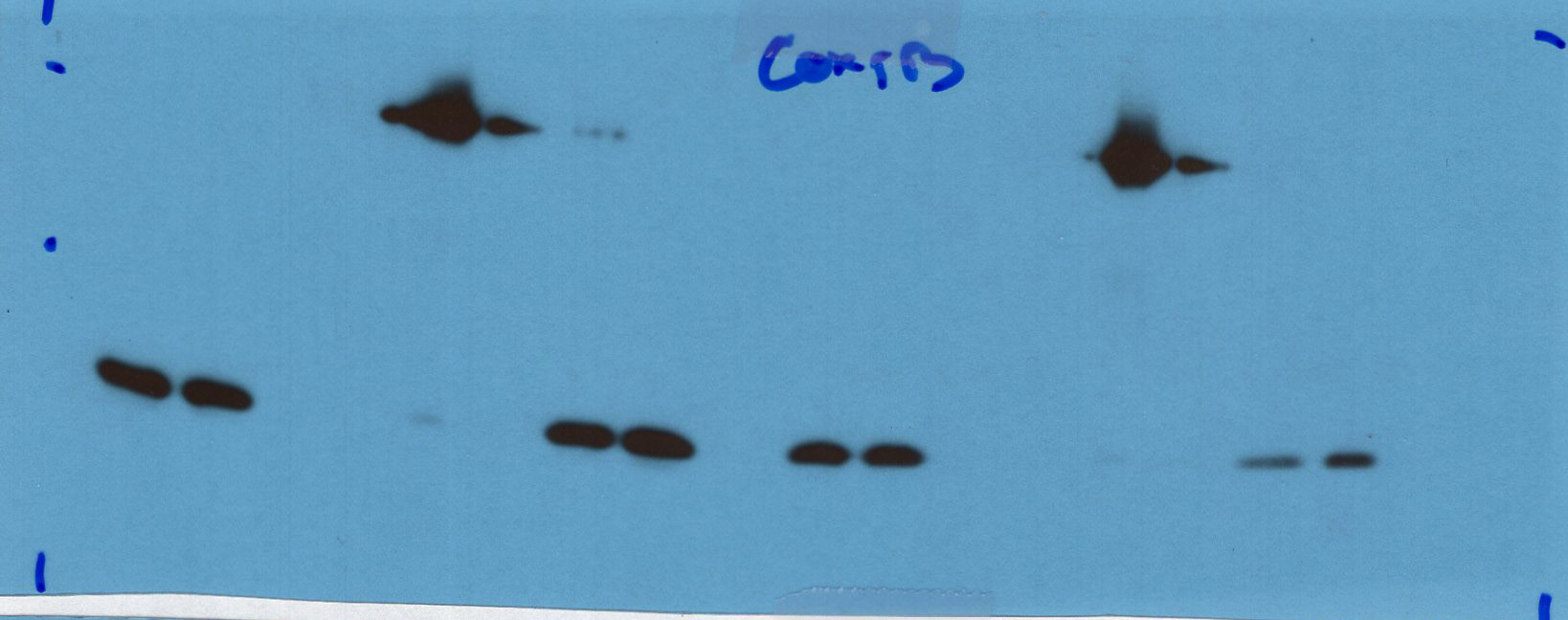

Supplement: Figure 5—figure supplement 1—source data 1. [file elife-78915-fig5-figsupp1-data1.zip › Fig 5-figure supplement 1-source data 1/Fig5s1C_cox5b.jpg]

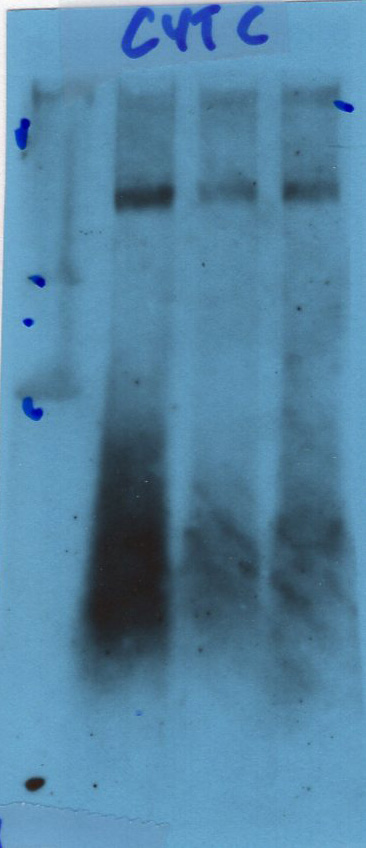

Supplement: Figure 5—figure supplement 1—source data 1. [file elife-78915-fig5-figsupp1-data1.zip › Fig 5-figure supplement 1-source data 1/Fig5s1E_cytc.jpg]

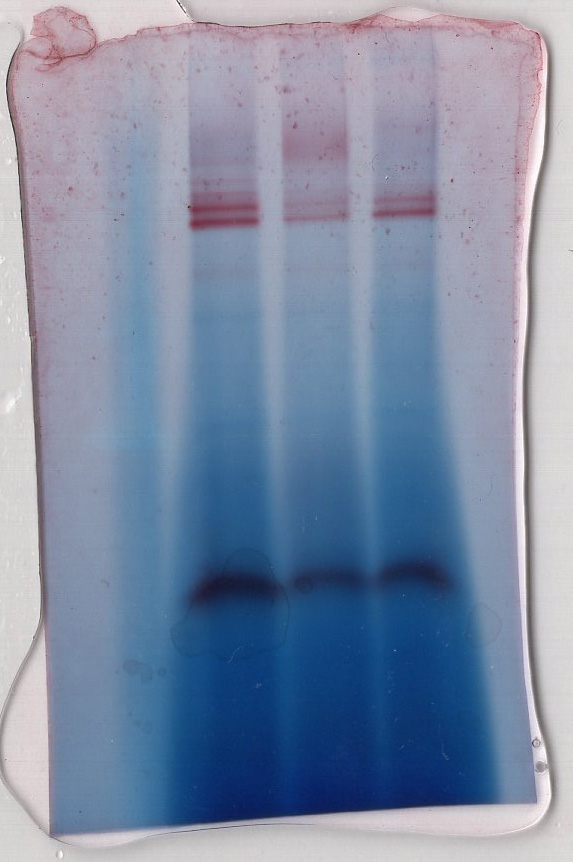

Supplement: Figure 5—figure supplement 1—source data 1. [file elife-78915-fig5-figsupp1-data1.zip › Fig 5-figure supplement 1-source data 1/Fig5s1E_IGACI.jpg]

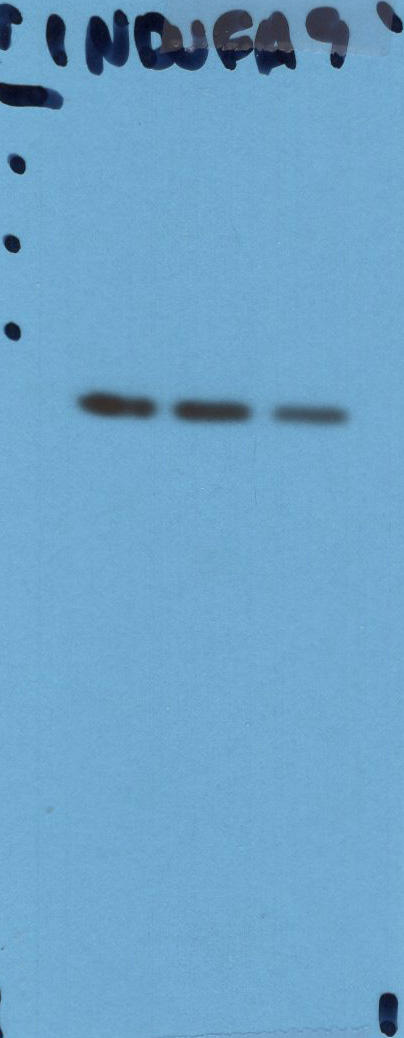

Supplement: Figure 5—figure supplement 1—source data 1. [file elife-78915-fig5-figsupp1-data1.zip › Fig 5-figure supplement 1-source data 1/Fig5s1B_ndufa9.jpg]

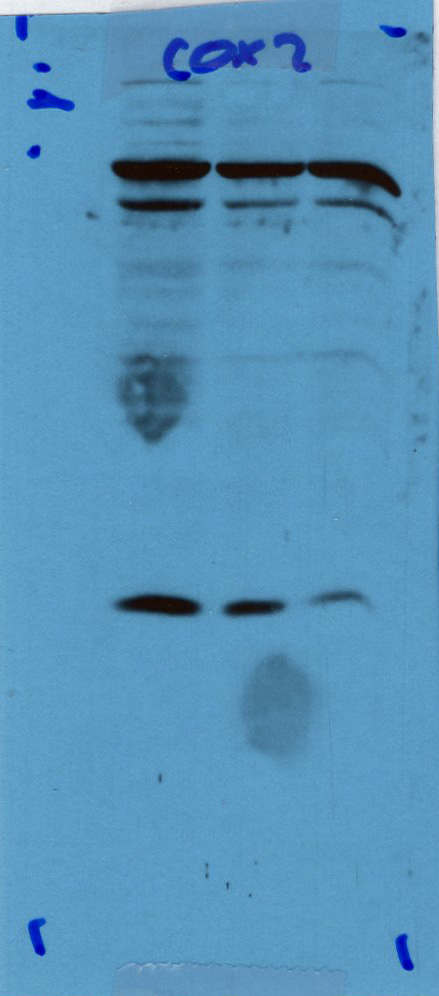

Supplement: Figure 5—figure supplement 1—source data 1. [file elife-78915-fig5-figsupp1-data1.zip › Fig 5-figure supplement 1-source data 1/Fig5s1B_cox2.jpg]

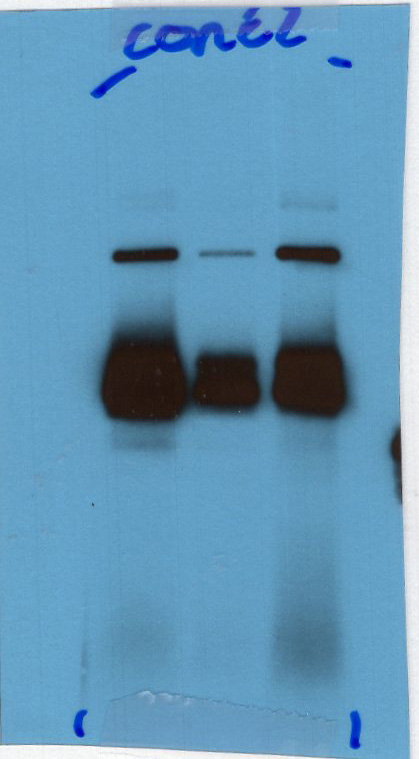

Supplement: Figure 5—figure supplement 1—source data 1. [file elife-78915-fig5-figsupp1-data1.zip › Fig 5-figure supplement 1-source data 1/Fig5s1D_core2.jpg]

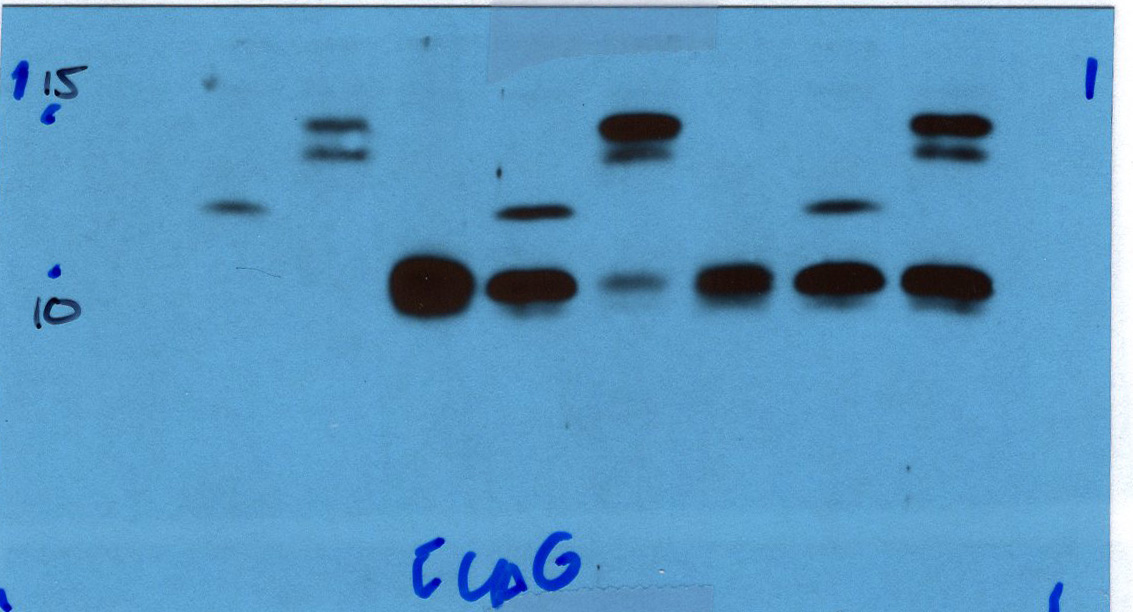

Supplement: Figure 5—figure supplement 1—source data 1. [file elife-78915-fig5-figsupp1-data1.zip › Fig 5-figure supplement 1-source data 1/Fig5s1A_flag.jpg]

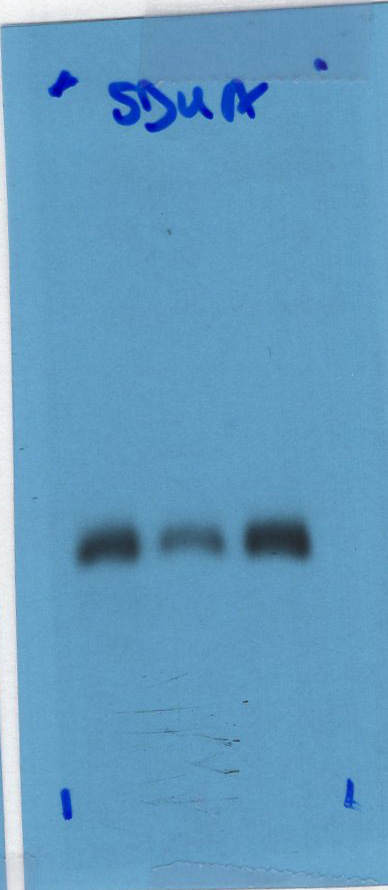

Supplement: Figure 5—figure supplement 1—source data 1. [file elife-78915-fig5-figsupp1-data1.zip › Fig 5-figure supplement 1-source data 1/Fig5s1E_sdha.jpg]

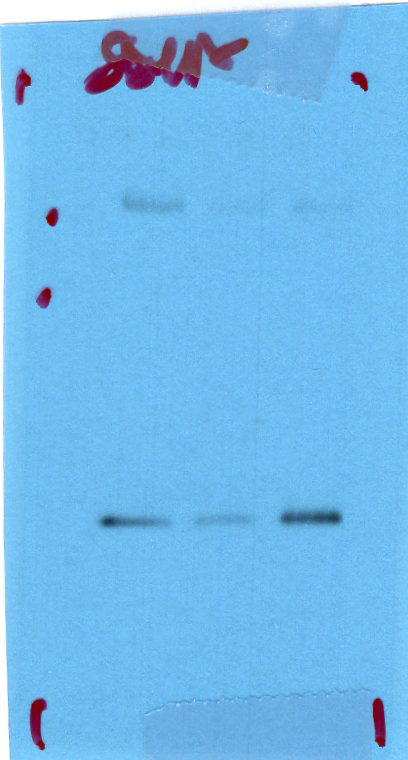

Supplement: Figure 5—figure supplement 1—source data 1. [file elife-78915-fig5-figsupp1-data1.zip › Fig 5-figure supplement 1-source data 1/Fig5s1D_sdha.jpg]

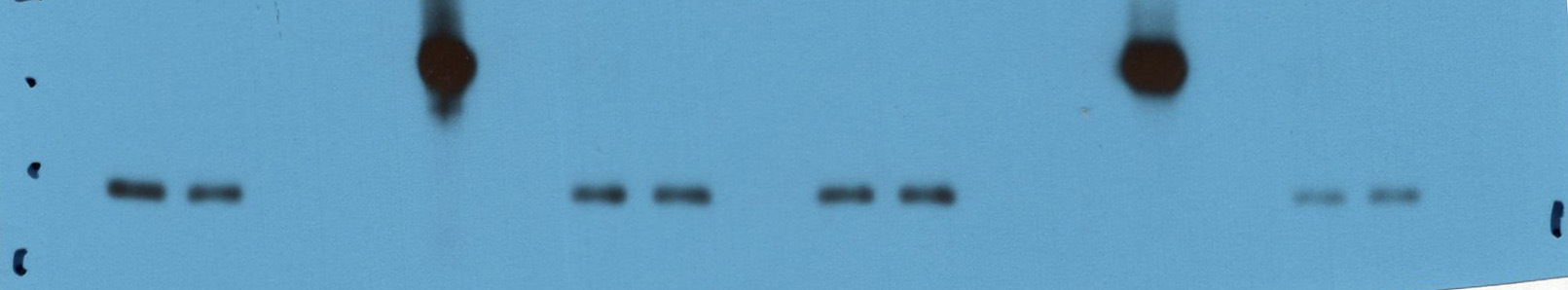

Supplement: Figure 5—figure supplement 1—source data 1. [file elife-78915-fig5-figsupp1-data1.zip › Fig 5-figure supplement 1-source data 1/Fig5s1C_cox1.jpg]

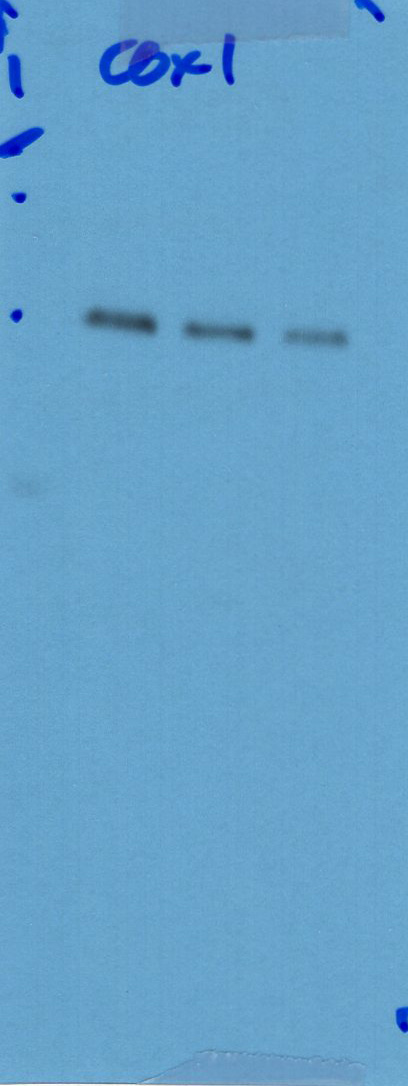

Supplement: Figure 5—figure supplement 1—source data 1. [file elife-78915-fig5-figsupp1-data1.zip › Fig 5-figure supplement 1-source data 1/Fig5s1B_cox1.jpg]

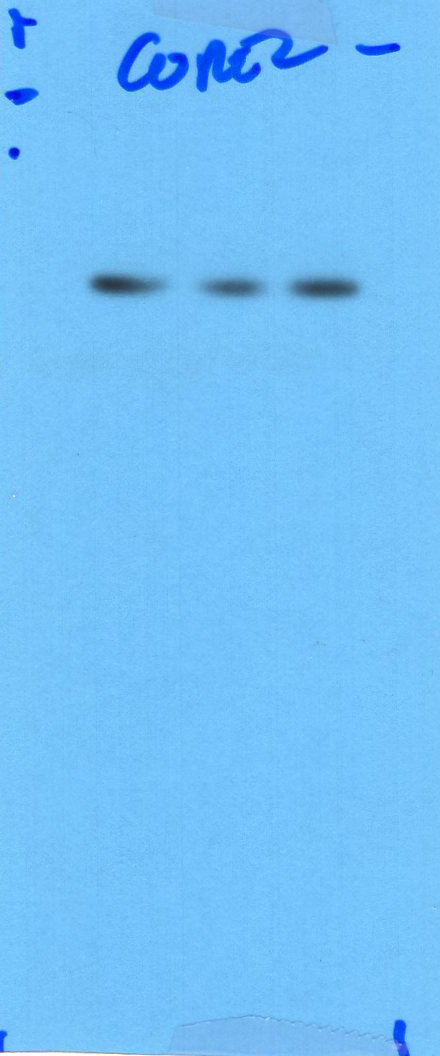

Supplement: Figure 5—figure supplement 1—source data 1. [file elife-78915-fig5-figsupp1-data1.zip › Fig 5-figure supplement 1-source data 1/Fig5s1B_core2.jpg]

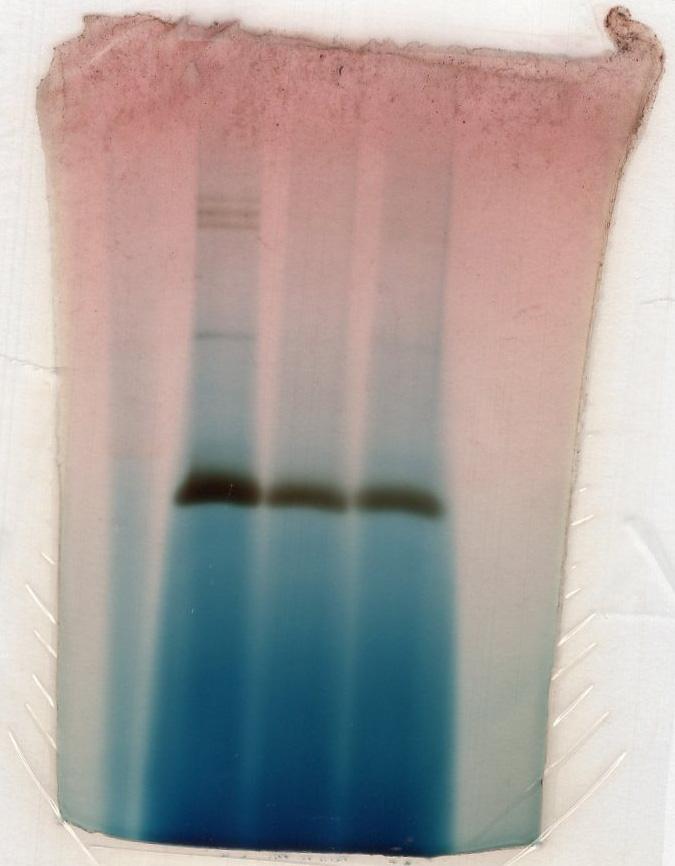

Supplement: Figure 5—figure supplement 1—source data 1. [file elife-78915-fig5-figsupp1-data1.zip › Fig 5-figure supplement 1-source data 1/Fig5s1E_IGACIV.jpg]

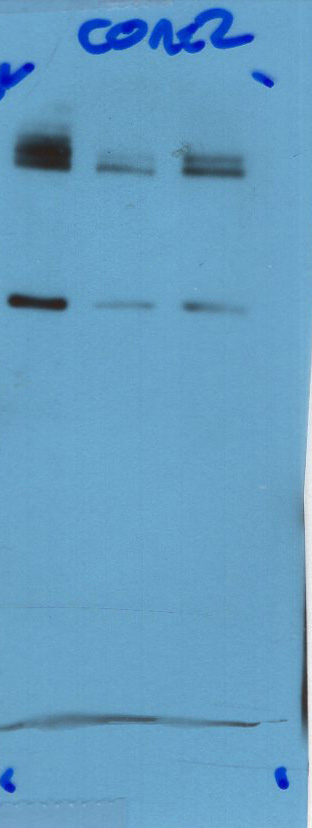

Supplement: Figure 5—figure supplement 1—source data 1. [file elife-78915-fig5-figsupp1-data1.zip › Fig 5-figure supplement 1-source data 1/Fig5s1E_core2.jpg]

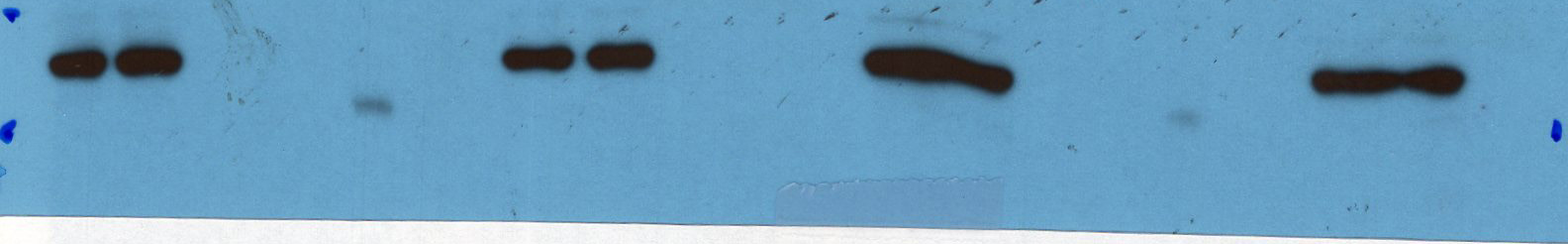

Supplement: Figure 5—figure supplement 1—source data 1. [file elife-78915-fig5-figsupp1-data1.zip › Fig 5-figure supplement 1-source data 1/Fig5s1C_uqcrb.jpg]

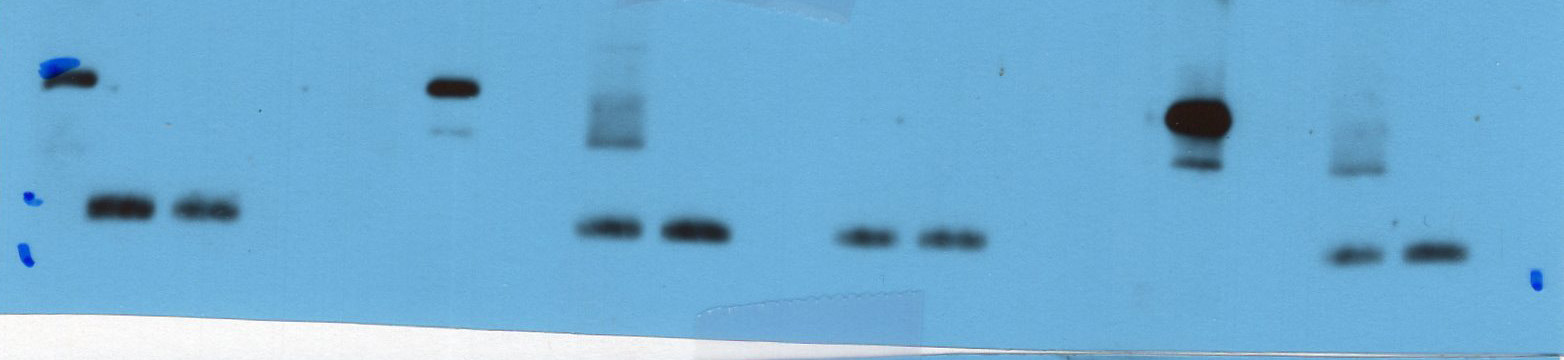

Supplement: Figure 5—figure supplement 1—source data 1. [file elife-78915-fig5-figsupp1-data1.zip › Fig 5-figure supplement 1-source data 1/Fig5s1C_core2.jpg]

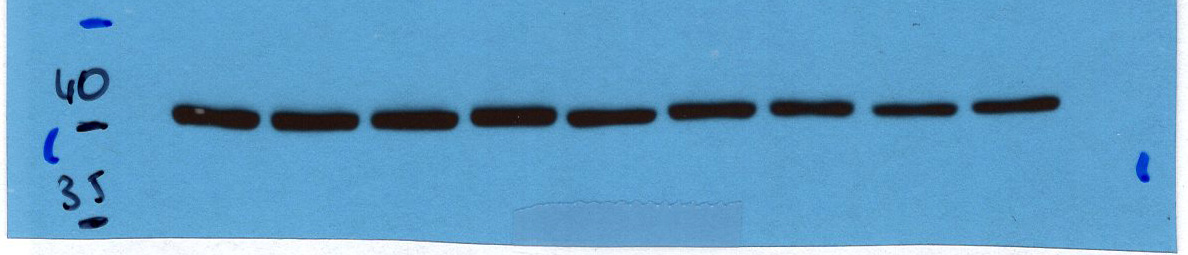

Supplement: Figure 5—figure supplement 1—source data 1. [file elife-78915-fig5-figsupp1-data1.zip › Fig 5-figure supplement 1-source data 1/Fig5s1A_actin.jpg]

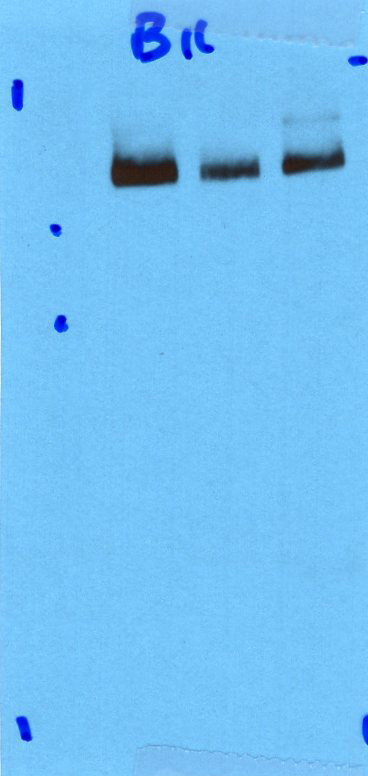

Supplement: Figure 5—figure supplement 1—source data 1. [file elife-78915-fig5-figsupp1-data1.zip › Fig 5-figure supplement 1-source data 1/Fig5s1D_ndufb11.jpg]

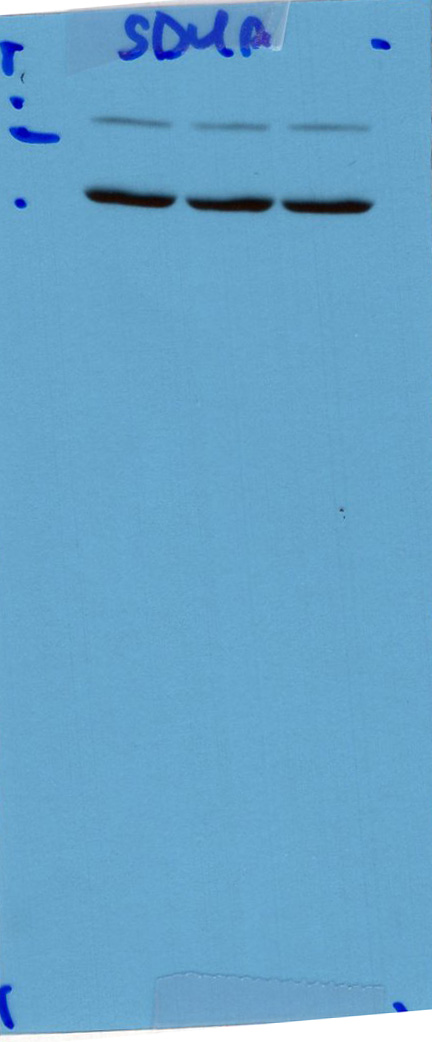

Supplement: Figure 5—figure supplement 1—source data 1. [file elife-78915-fig5-figsupp1-data1.zip › Fig 5-figure supplement 1-source data 1/Fig5s1B_sdha.jpg]

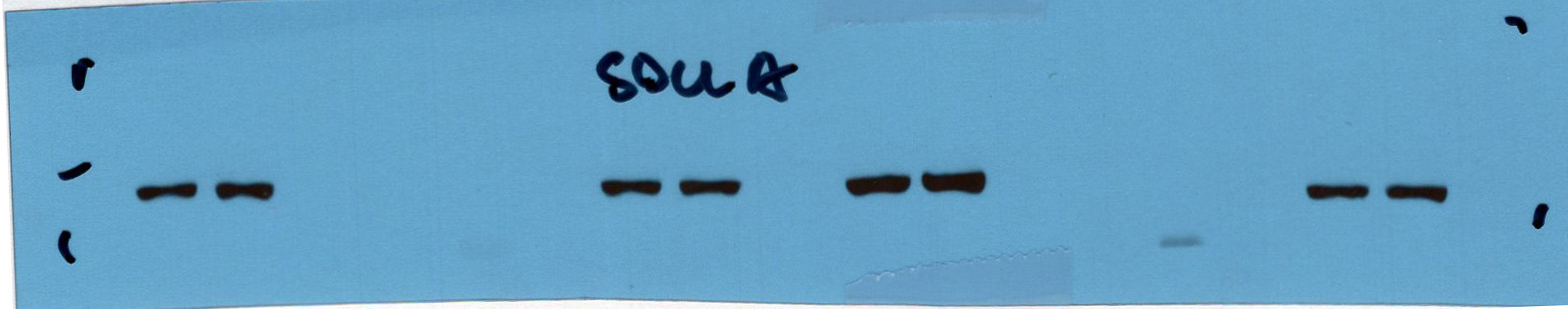

Supplement: Figure 5—figure supplement 1—source data 1. [file elife-78915-fig5-figsupp1-data1.zip › Fig 5-figure supplement 1-source data 1/Fig5s1C_sdha.jpg]

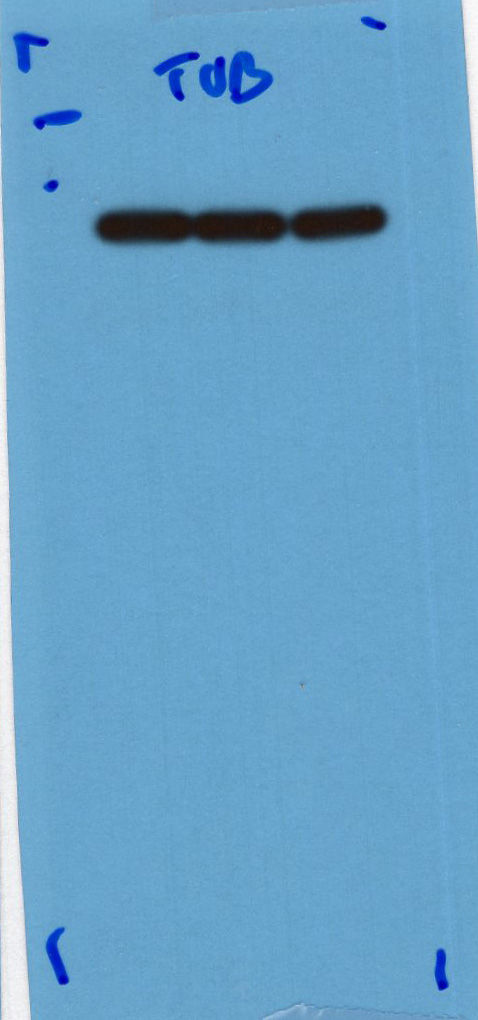

Supplement: Figure 5—figure supplement 1—source data 1. [file elife-78915-fig5-figsupp1-data1.zip › Fig 5-figure supplement 1-source data 1/Fig5s1B_tub.jpg]

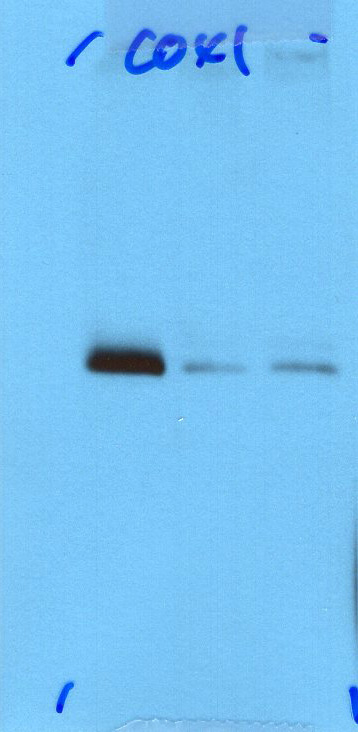

Supplement: Figure 5—figure supplement 1—source data 1. [file elife-78915-fig5-figsupp1-data1.zip › Fig 5-figure supplement 1-source data 1/Fig5s1D_cox1.jpg]

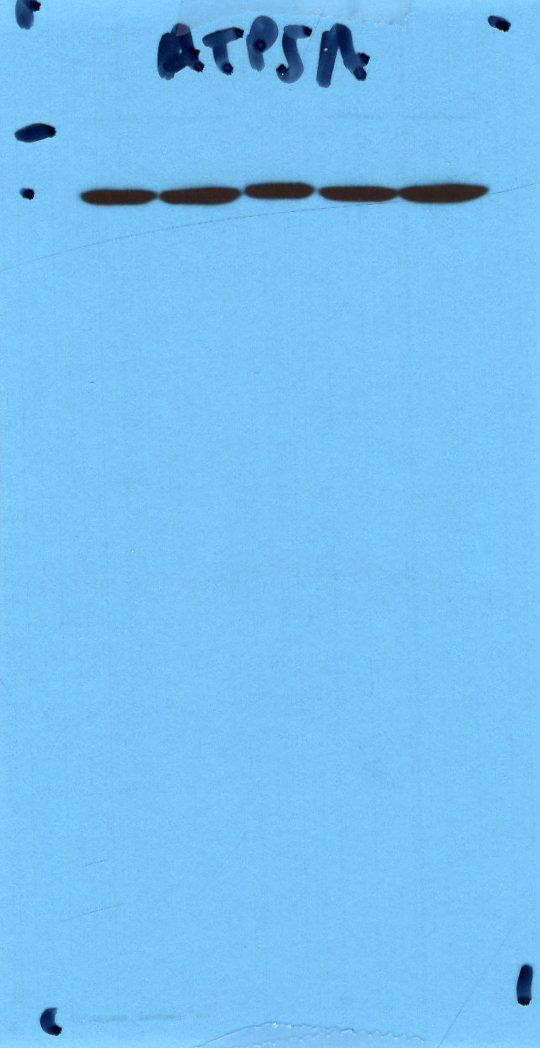

Supplement: Figure 5—figure supplement 2—source data 1. [file elife-78915-fig5-figsupp2-data1.zip › Fig 5-figure supplement 2-source data 1/Fig5s2A_atp5a.jpg]

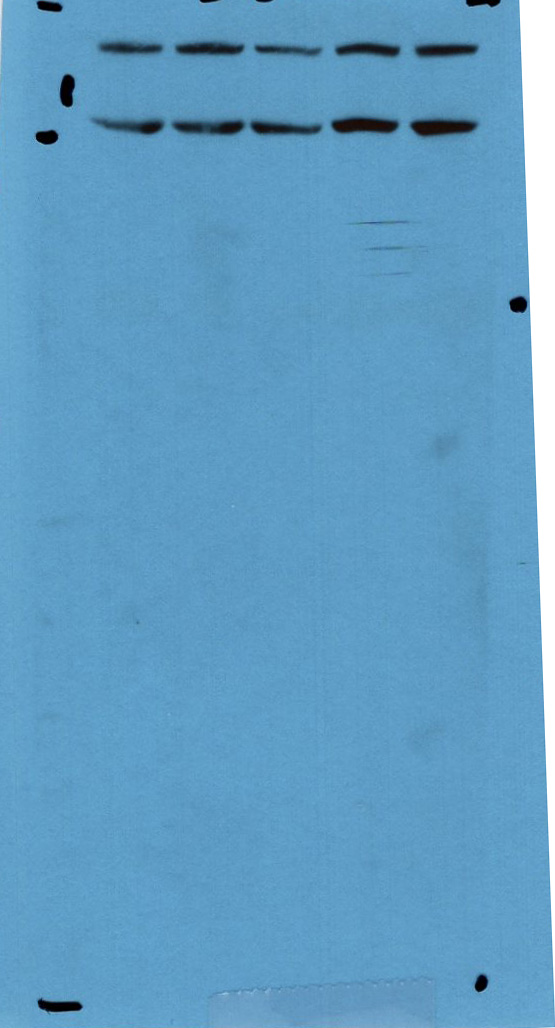

Supplement: Figure 5—figure supplement 2—source data 1. [file elife-78915-fig5-figsupp2-data1.zip › Fig 5-figure supplement 2-source data 1/Fig5s2A_tub.jpg]

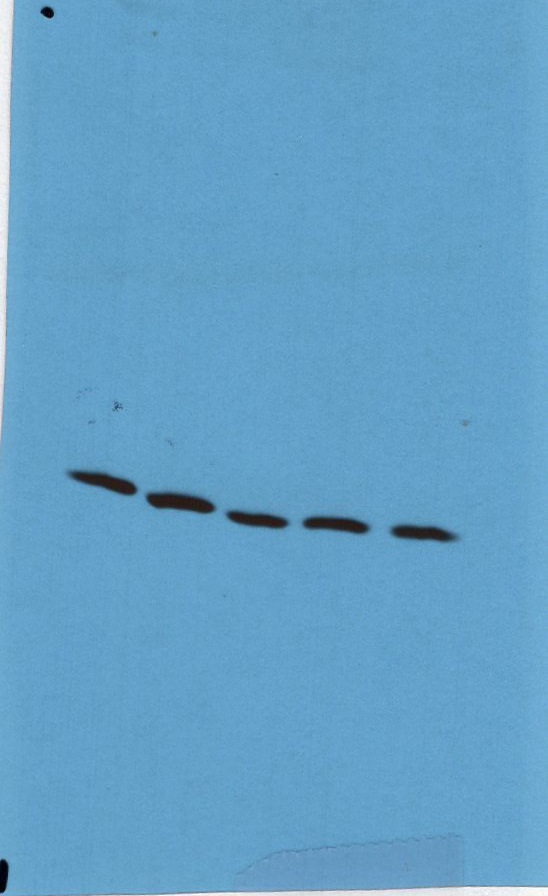

Supplement: Figure 5—figure supplement 2—source data 1. [file elife-78915-fig5-figsupp2-data1.zip › Fig 5-figure supplement 2-source data 1/Fig5s2A_ndufa9.jpg]

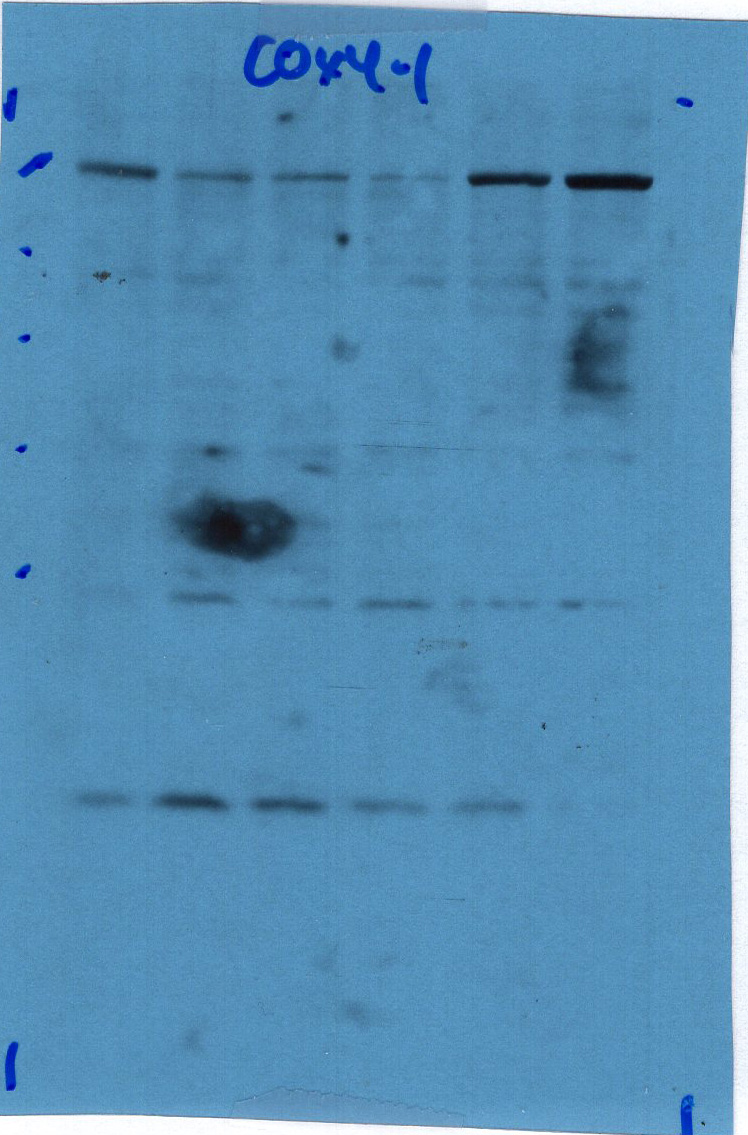

Supplement: Figure 5—figure supplement 2—source data 1. [file elife-78915-fig5-figsupp2-data1.zip › Fig 5-figure supplement 2-source data 1/Fig5s2C_cox4i1.jpg]

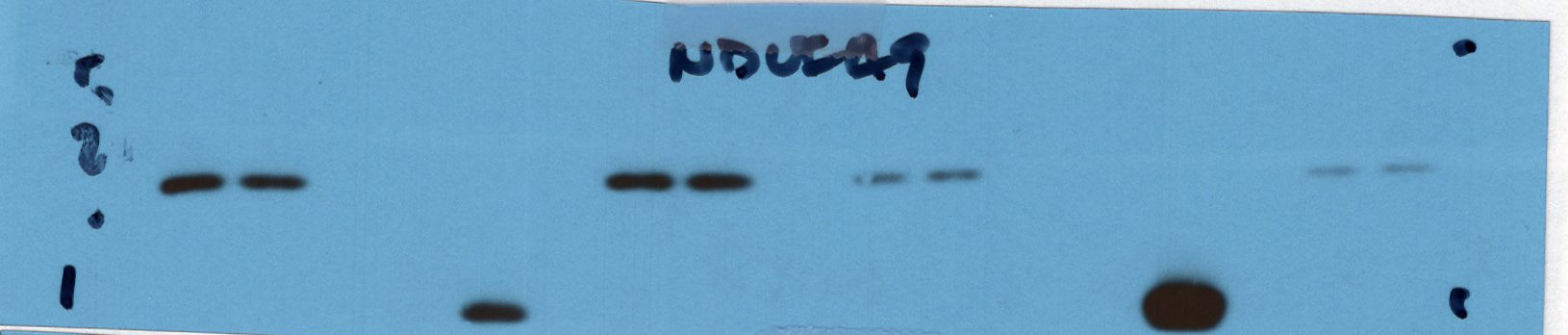

Supplement: Figure 5—figure supplement 2—source data 1. [file elife-78915-fig5-figsupp2-data1.zip › Fig 5-figure supplement 2-source data 1/Fig5s2D_ndufa9.jpg]

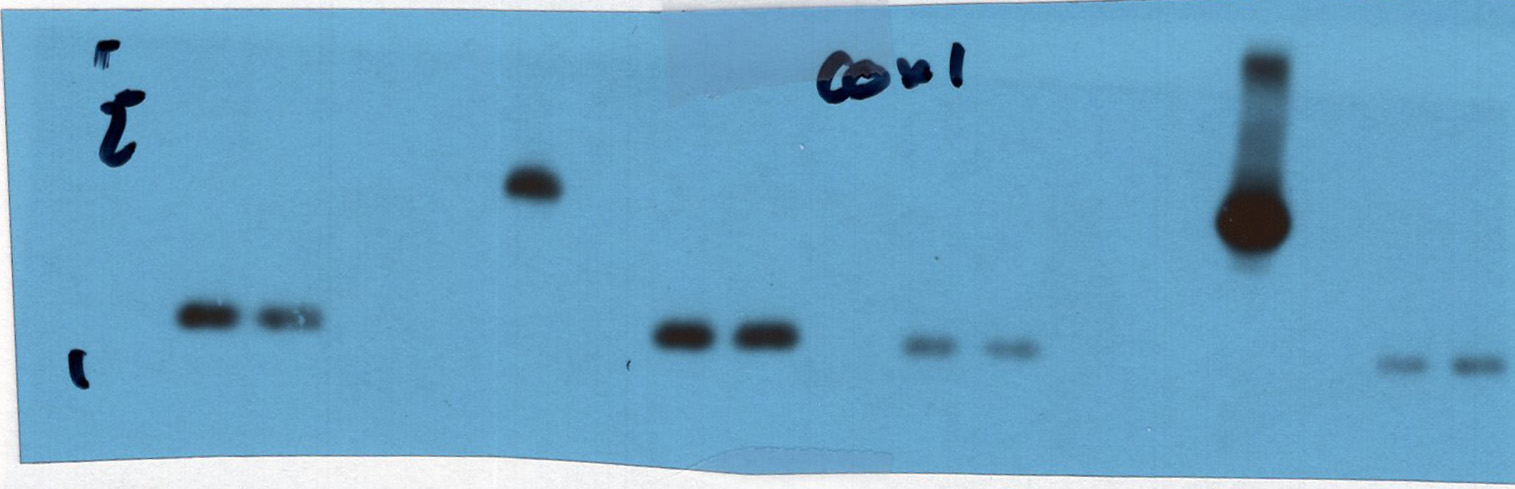

Supplement: Figure 5—figure supplement 2—source data 1. [file elife-78915-fig5-figsupp2-data1.zip › Fig 5-figure supplement 2-source data 1/Fig5s2D_cox1.jpg]

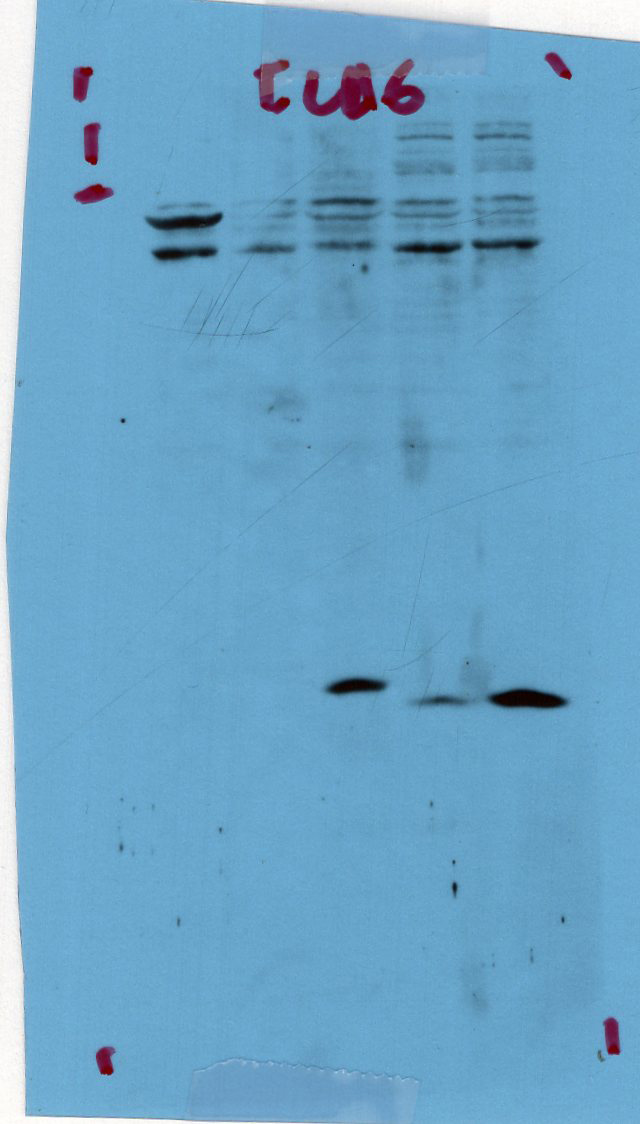

Supplement: Figure 5—figure supplement 2—source data 1. [file elife-78915-fig5-figsupp2-data1.zip › Fig 5-figure supplement 2-source data 1/Fig5s2A_flag.jpg]

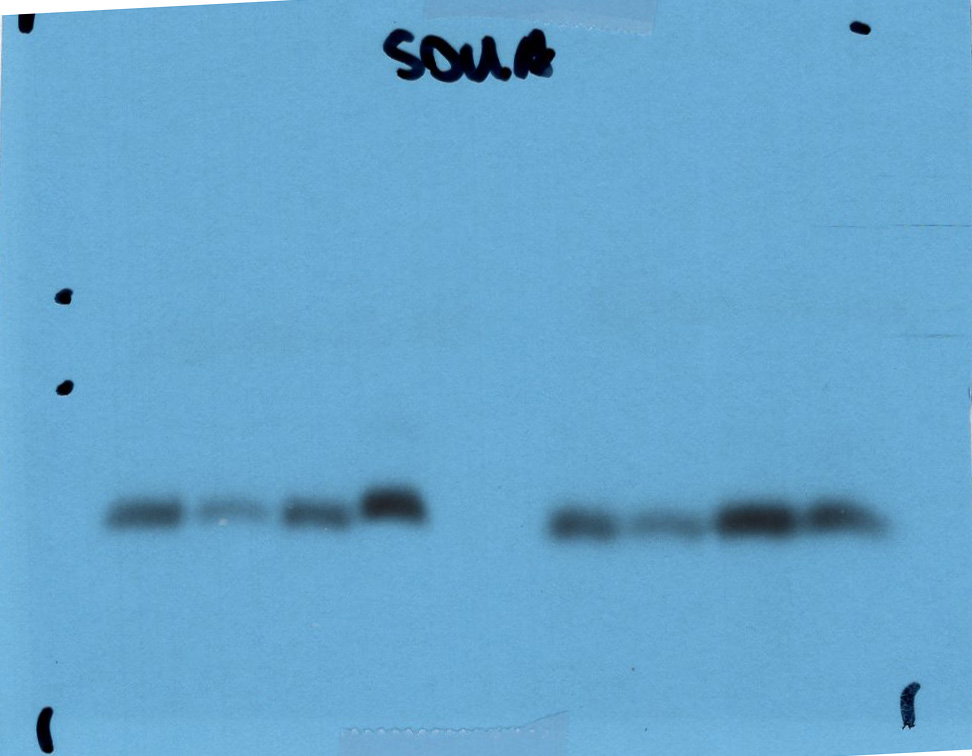

Supplement: Figure 5—figure supplement 2—source data 1. [file elife-78915-fig5-figsupp2-data1.zip › Fig 5-figure supplement 2-source data 1/Fig5s2E_sdha.jpg]

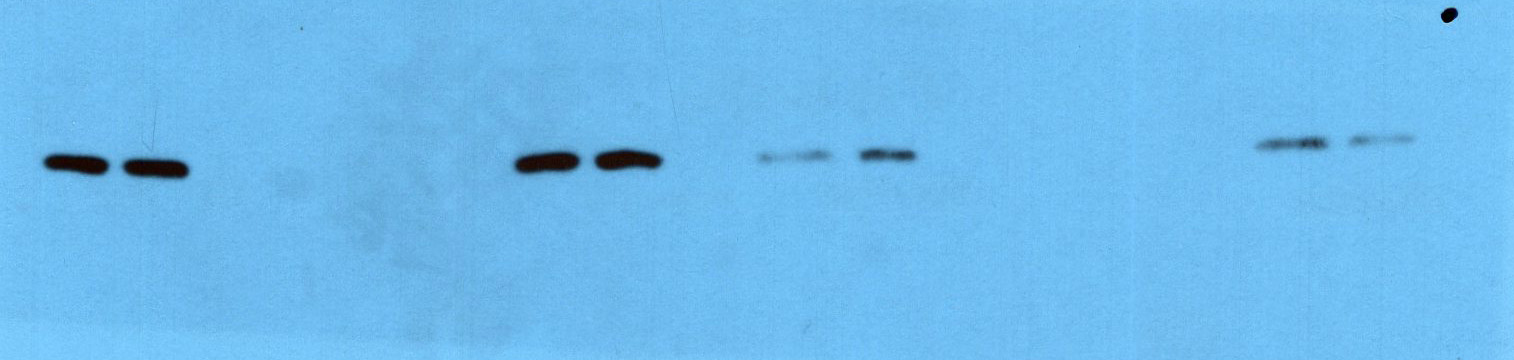

Supplement: Figure 5—figure supplement 2—source data 1. [file elife-78915-fig5-figsupp2-data1.zip › Fig 5-figure supplement 2-source data 1/Fig5s2D_cox4i1.jpg]

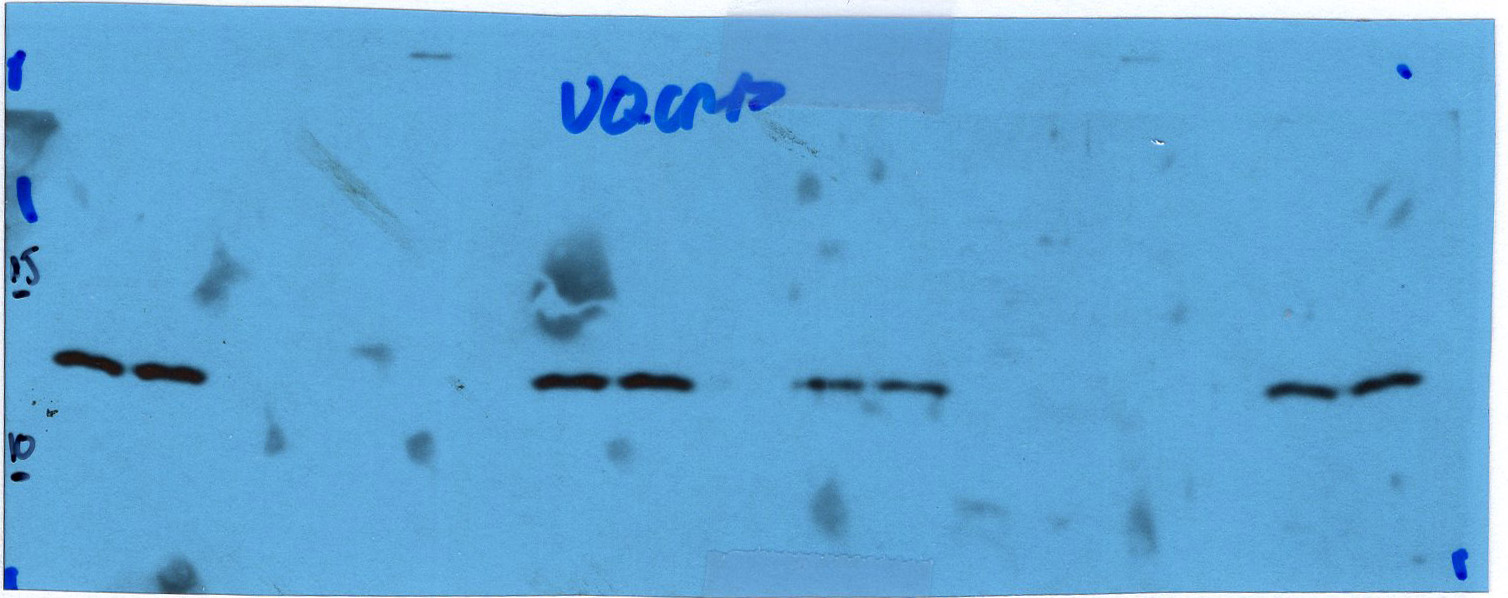

Supplement: Figure 5—figure supplement 2—source data 1. [file elife-78915-fig5-figsupp2-data1.zip › Fig 5-figure supplement 2-source data 1/Fig5s2D_uqcrb.jpg]

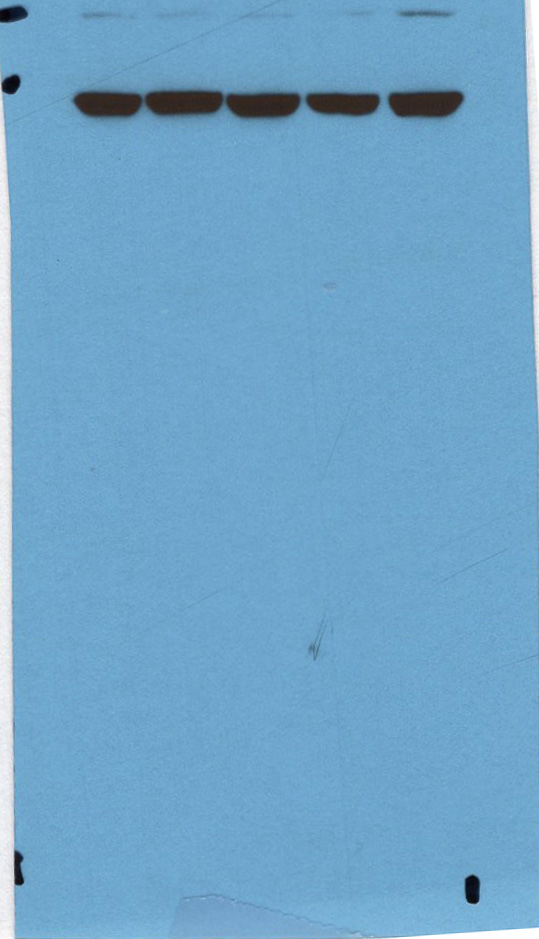

Supplement: Figure 5—figure supplement 2—source data 1. [file elife-78915-fig5-figsupp2-data1.zip › Fig 5-figure supplement 2-source data 1/Fig5s2A_sdha.jpg]

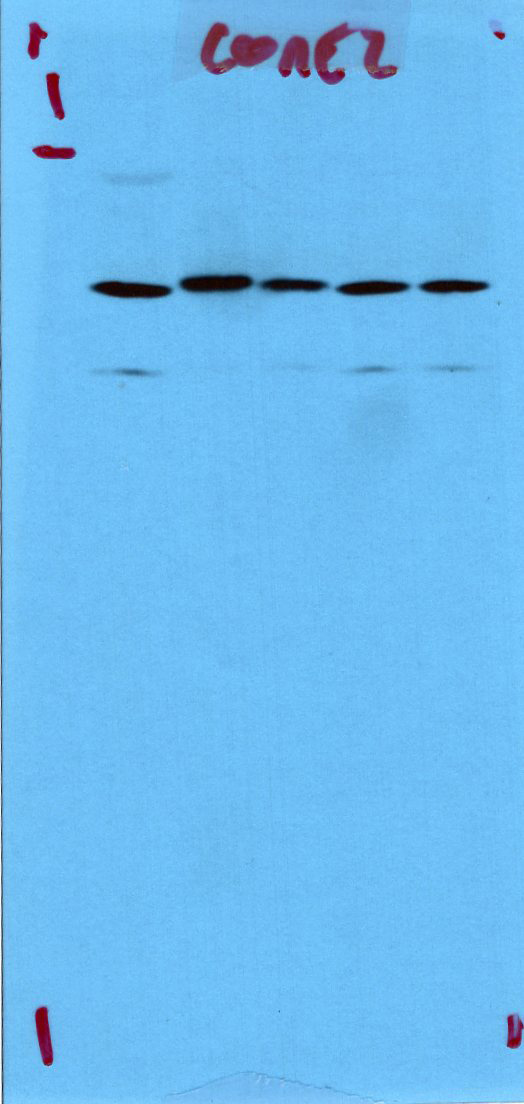

Supplement: Figure 5—figure supplement 2—source data 1. [file elife-78915-fig5-figsupp2-data1.zip › Fig 5-figure supplement 2-source data 1/Fig5s2A_core2.jpg]

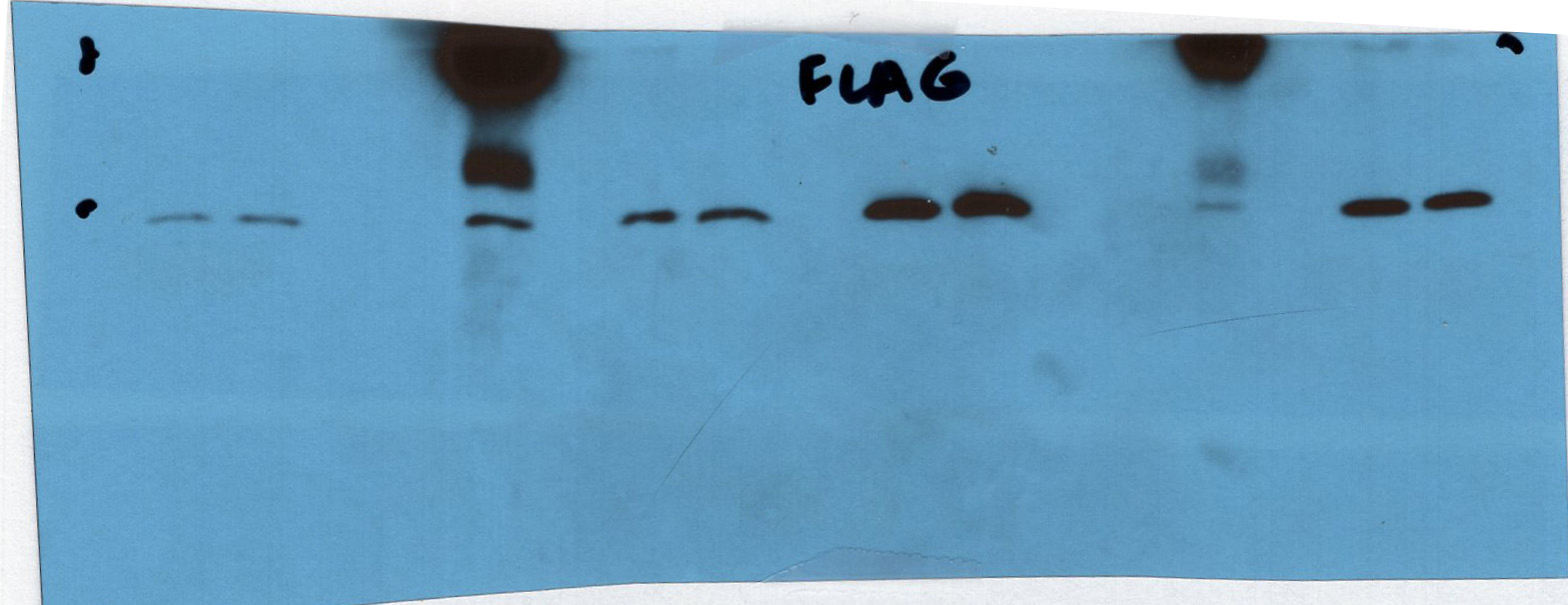

Supplement: Figure 5—figure supplement 2—source data 1. [file elife-78915-fig5-figsupp2-data1.zip › Fig 5-figure supplement 2-source data 1/Fig5s2D_flag.jpg]

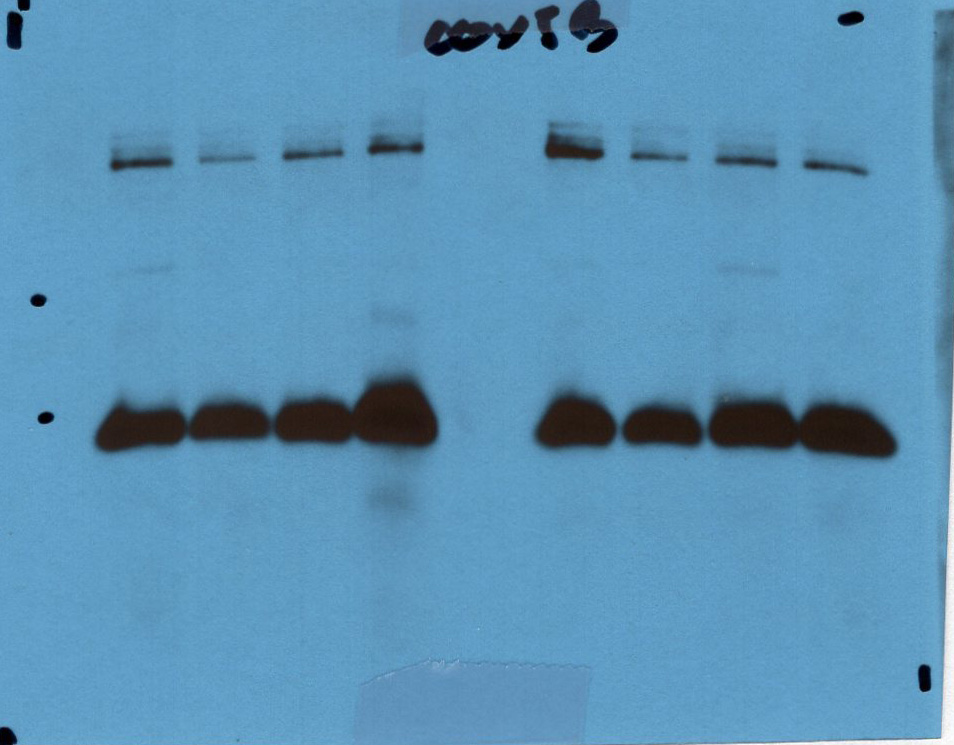

Supplement: Figure 5—figure supplement 2—source data 1. [file elife-78915-fig5-figsupp2-data1.zip › Fig 5-figure supplement 2-source data 1/Fig5s2E_cox5b.jpg]

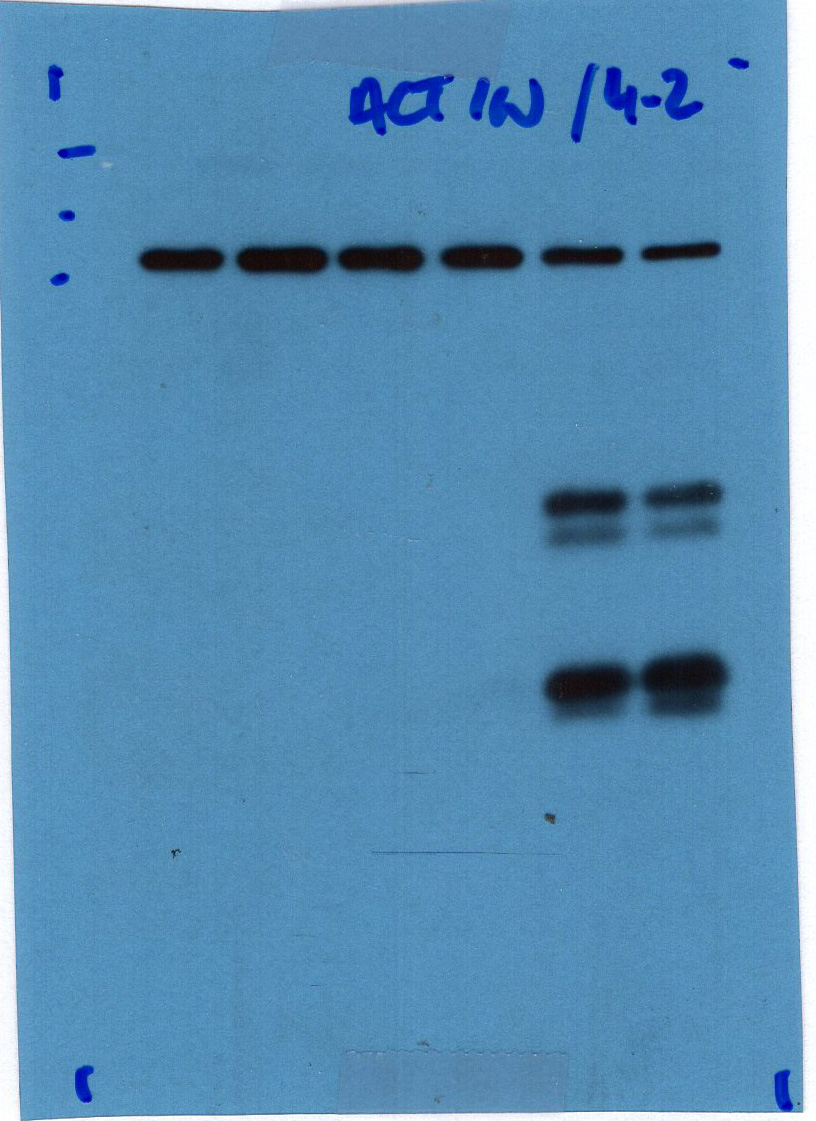

Supplement: Figure 5—figure supplement 2—source data 1. [file elife-78915-fig5-figsupp2-data1.zip › Fig 5-figure supplement 2-source data 1/Fig5s2C_cox4i2_actin.jpg]

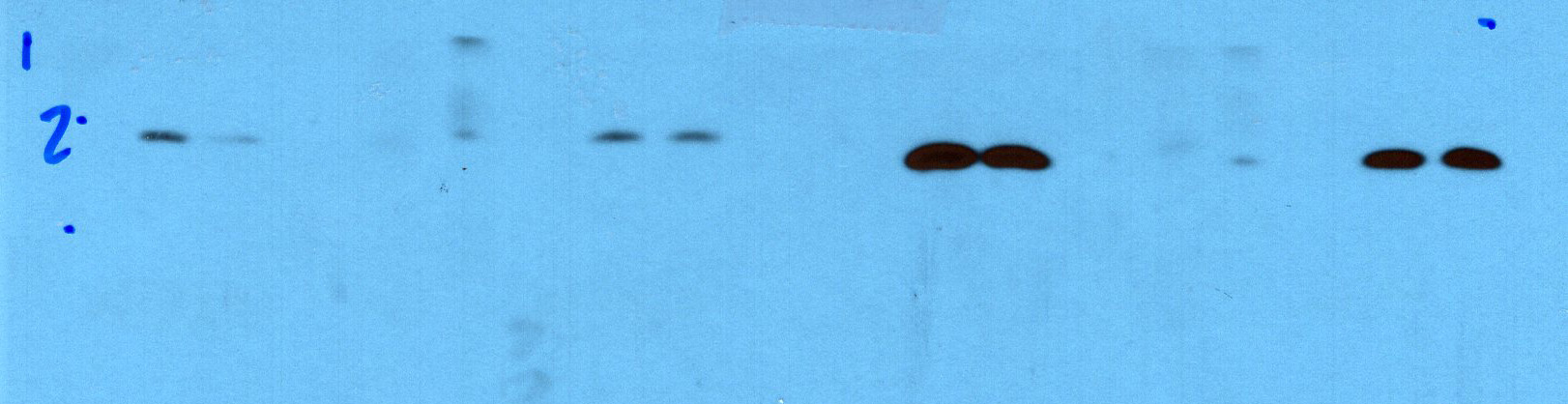

Supplement: Figure 5—figure supplement 2—source data 1. [file elife-78915-fig5-figsupp2-data1.zip › Fig 5-figure supplement 2-source data 1/Fig5s2D_cytc.jpg]

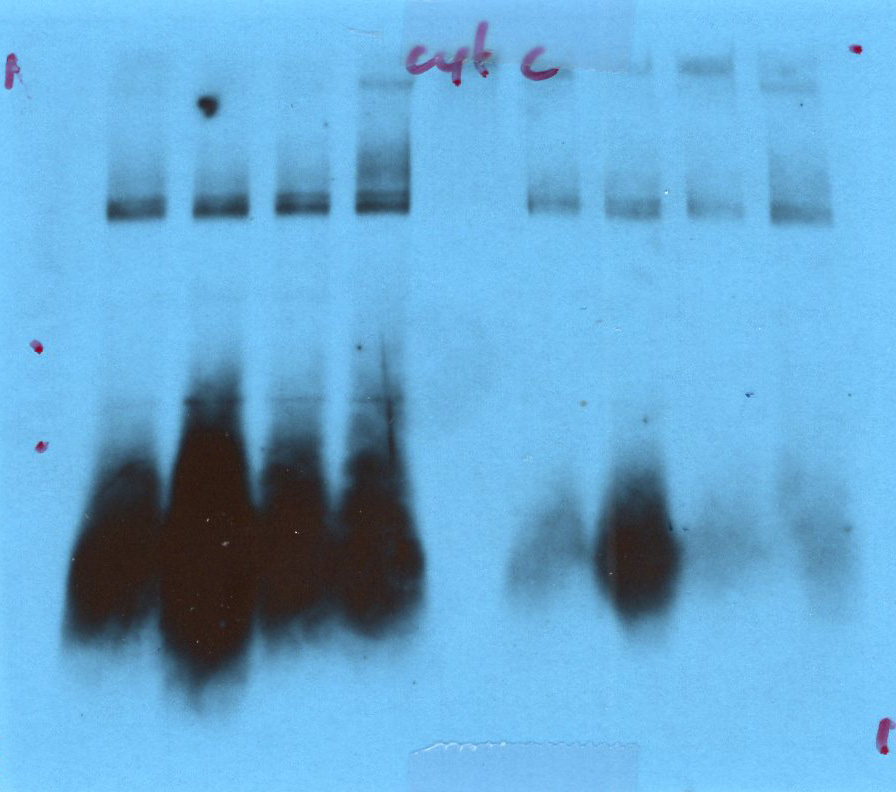

Supplement: Figure 5—figure supplement 2—source data 1. [file elife-78915-fig5-figsupp2-data1.zip › Fig 5-figure supplement 2-source data 1/Fig5s2E_cytc.jpg]

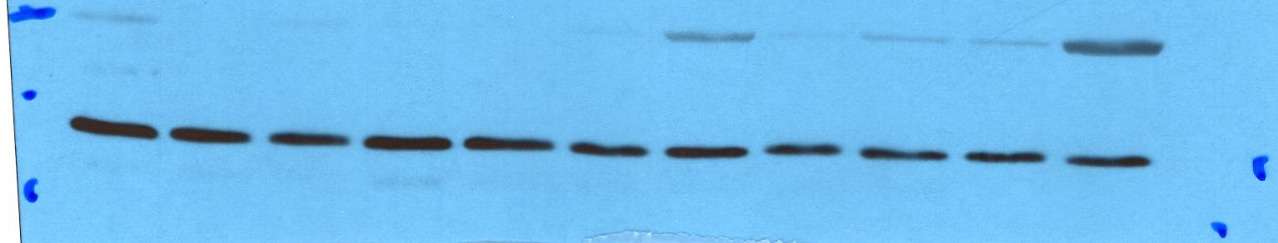

Supplement: Figure 5—figure supplement 2—source data 1. [file elife-78915-fig5-figsupp2-data1.zip › Fig 5-figure supplement 2-source data 1/Fig5s2B_actin.jpg]

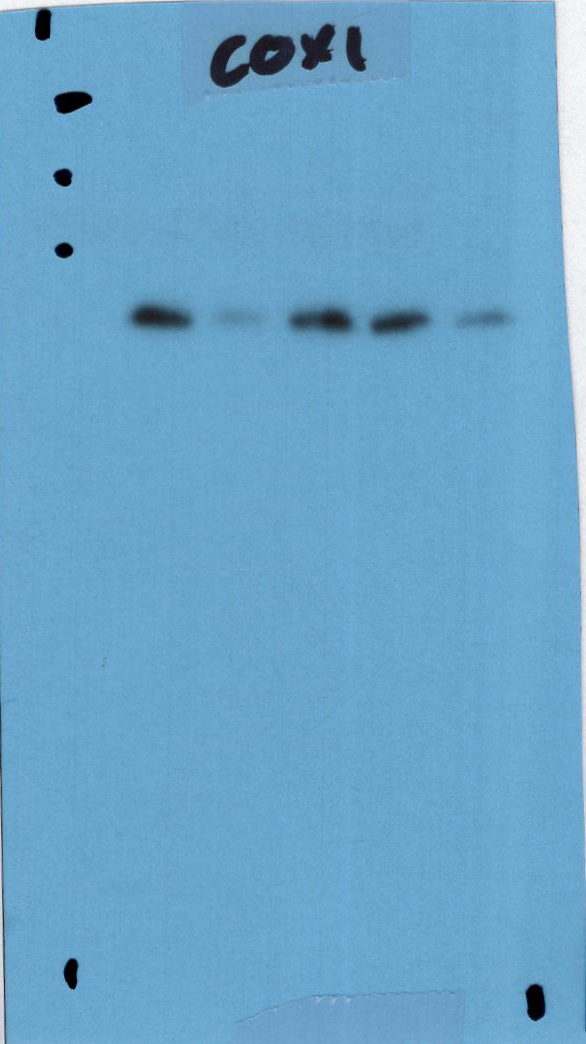

Supplement: Figure 5—figure supplement 2—source data 1. [file elife-78915-fig5-figsupp2-data1.zip › Fig 5-figure supplement 2-source data 1/Fig5s2A_cox1.jpg]

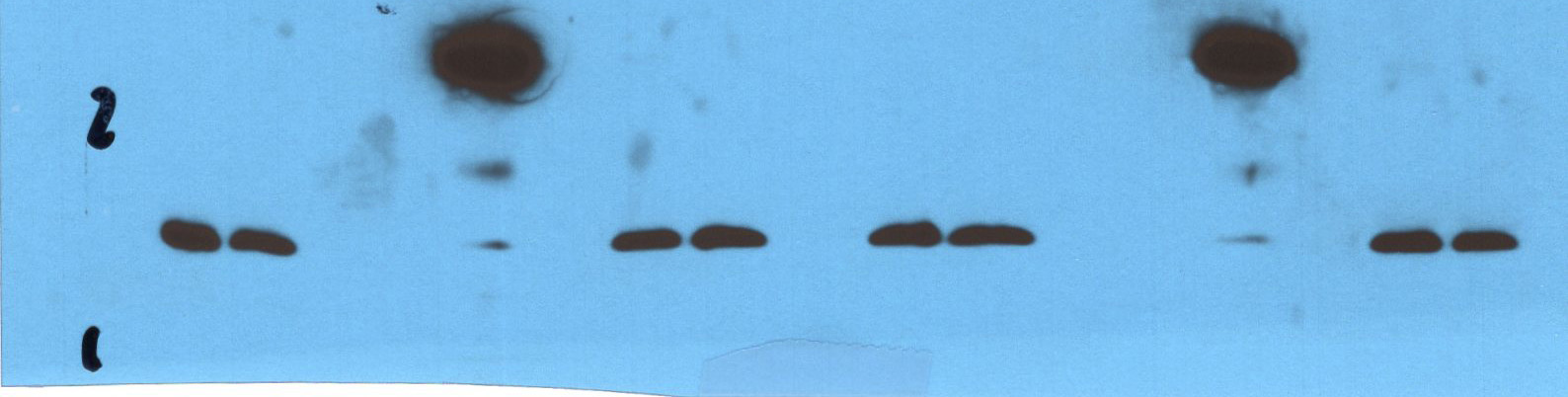

Supplement: Figure 5—figure supplement 2—source data 1. [file elife-78915-fig5-figsupp2-data1.zip › Fig 5-figure supplement 2-source data 1/Fig5s2D_cox5b.jpg]

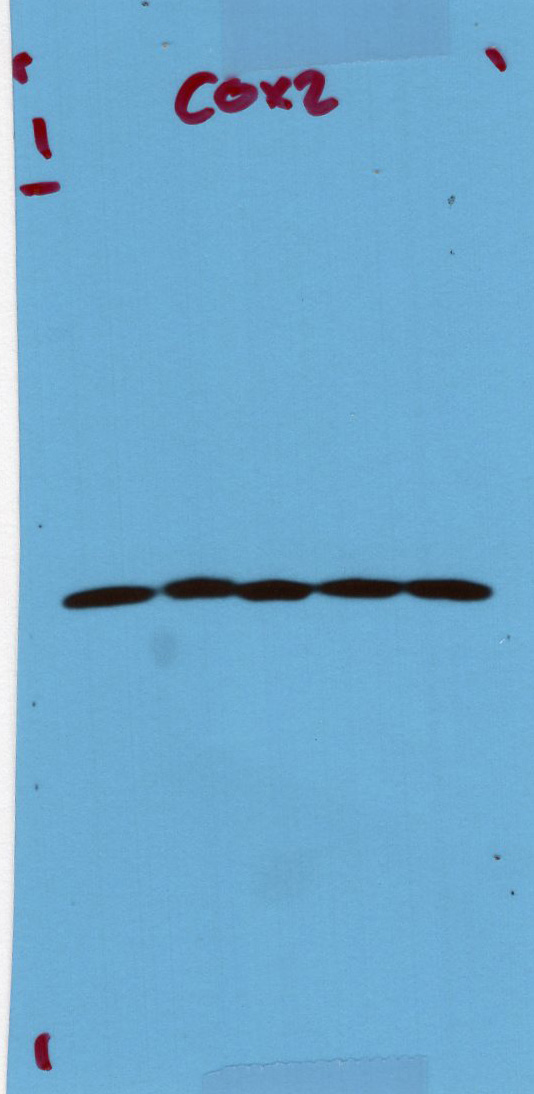

Supplement: Figure 5—figure supplement 2—source data 1. [file elife-78915-fig5-figsupp2-data1.zip › Fig 5-figure supplement 2-source data 1/Fig5s2A_cox2.jpg]

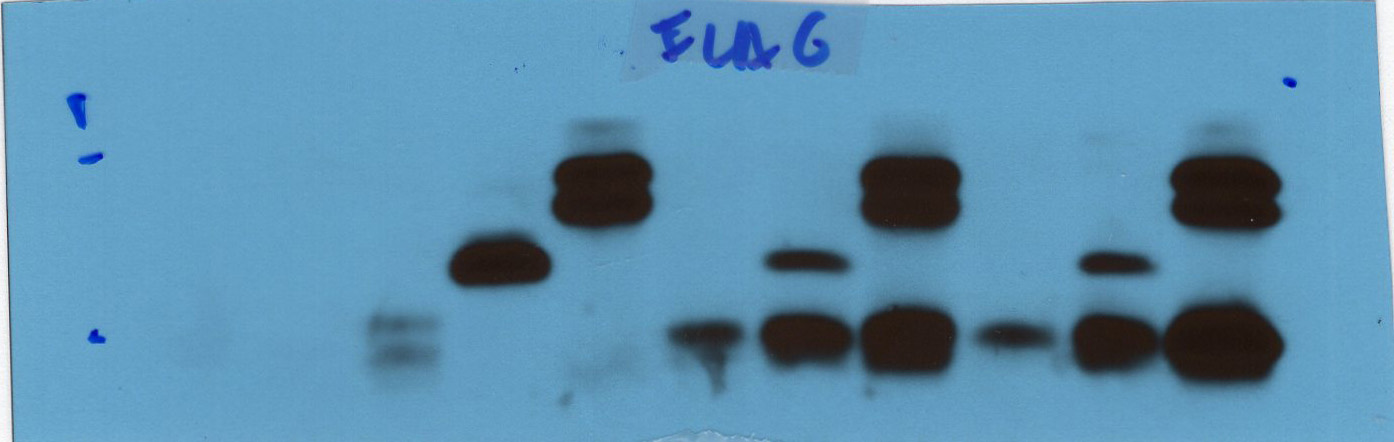

Supplement: Figure 5—figure supplement 2—source data 1. [file elife-78915-fig5-figsupp2-data1.zip › Fig 5-figure supplement 2-source data 1/Fig5s2B_flag.jpg]

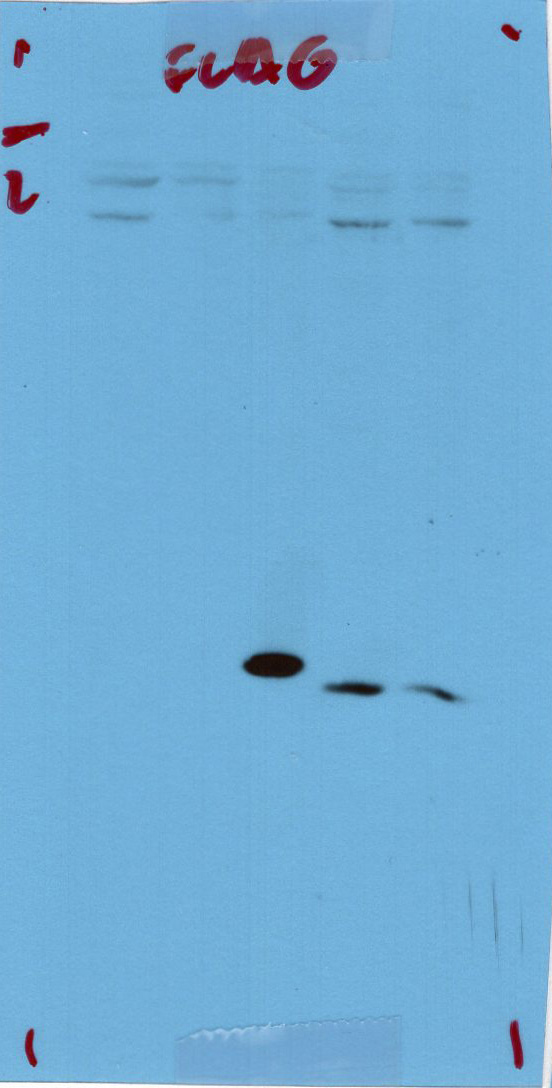

Supplement: Figure 5—figure supplement 3—source data 1. [file elife-78915-fig5-figsupp3-data1.zip › Fig 5-figure supplement 3-source data 1/Fig5s3A_flag.jpg]

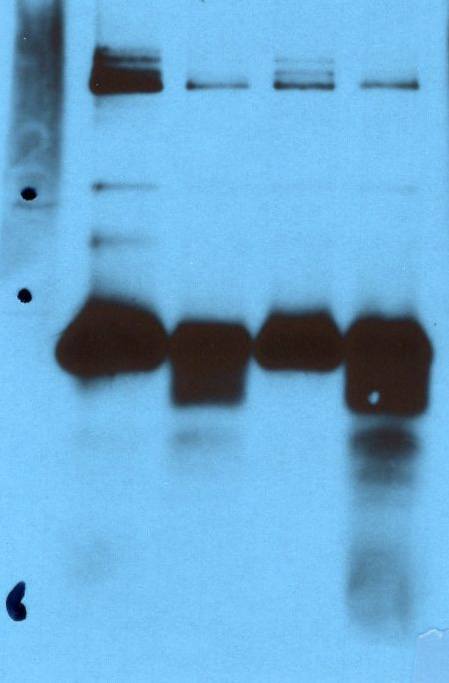

Supplement: Figure 5—figure supplement 3—source data 1. [file elife-78915-fig5-figsupp3-data1.zip › Fig 5-figure supplement 3-source data 1/Fig5s3D_Digcox5b1.jpg]

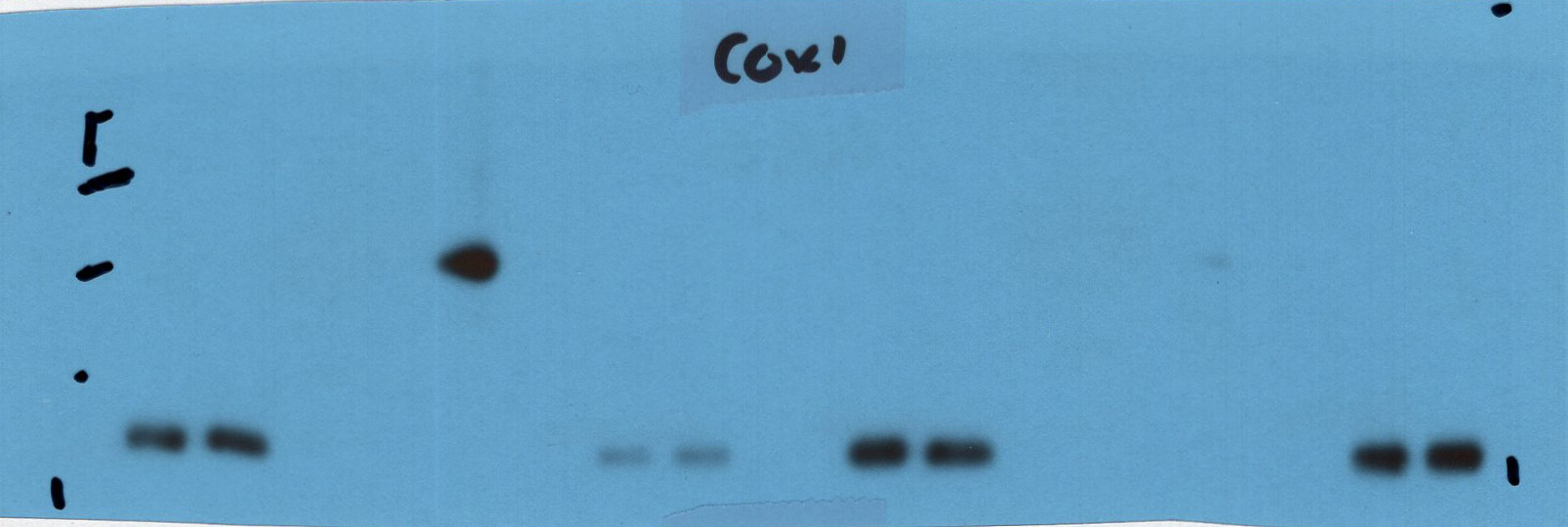

Supplement: Figure 5—figure supplement 3—source data 1. [file elife-78915-fig5-figsupp3-data1.zip › Fig 5-figure supplement 3-source data 1/Fig5s3C_cox1.jpg]

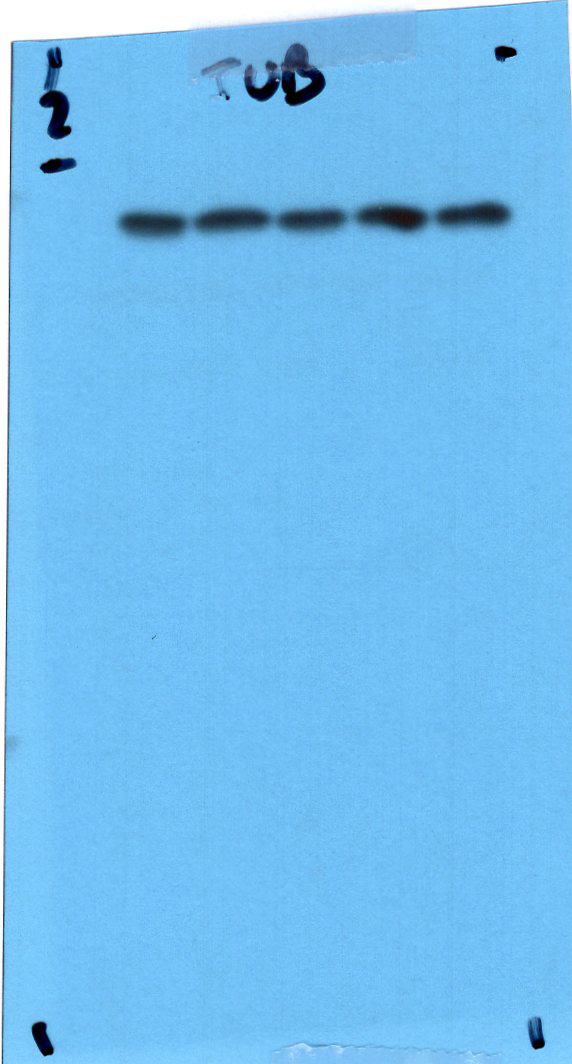

Supplement: Figure 5—figure supplement 3—source data 1. [file elife-78915-fig5-figsupp3-data1.zip › Fig 5-figure supplement 3-source data 1/Fig5s3A_tub.jpg]

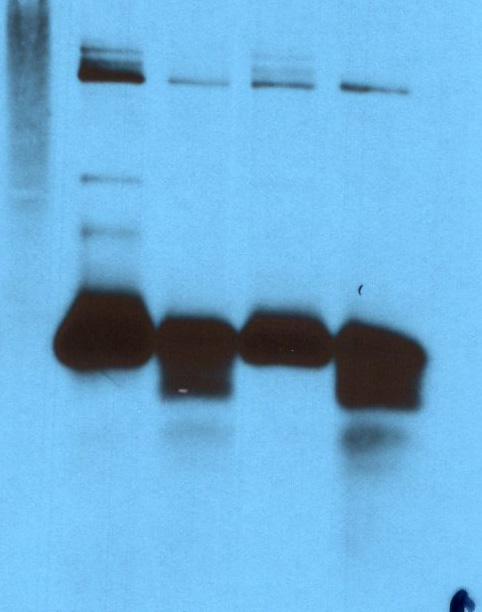

Supplement: Figure 5—figure supplement 3—source data 1. [file elife-78915-fig5-figsupp3-data1.zip › Fig 5-figure supplement 3-source data 1/Fig5s3D_Digcox5b2.jpg]

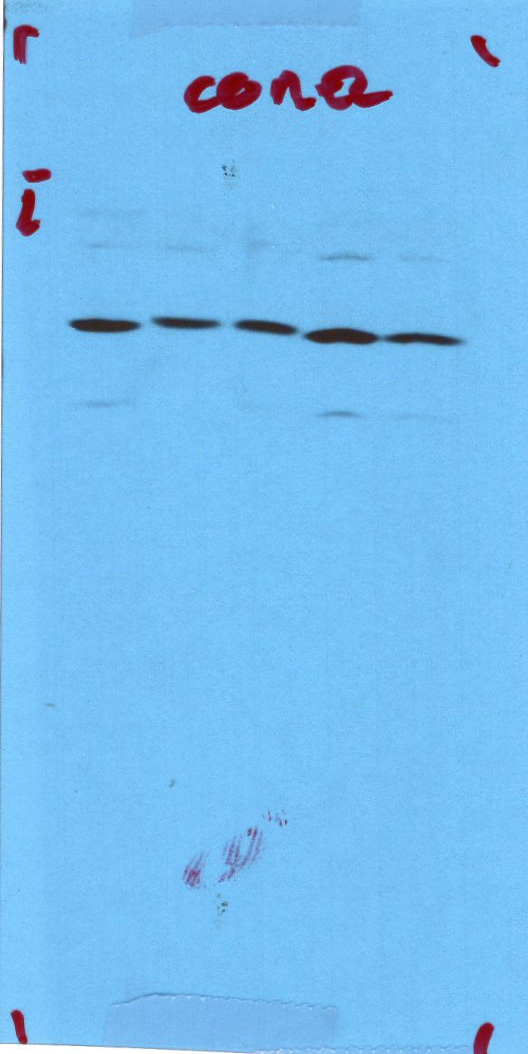

Supplement: Figure 5—figure supplement 3—source data 1. [file elife-78915-fig5-figsupp3-data1.zip › Fig 5-figure supplement 3-source data 1/Fig5s3A_core2.jpg]

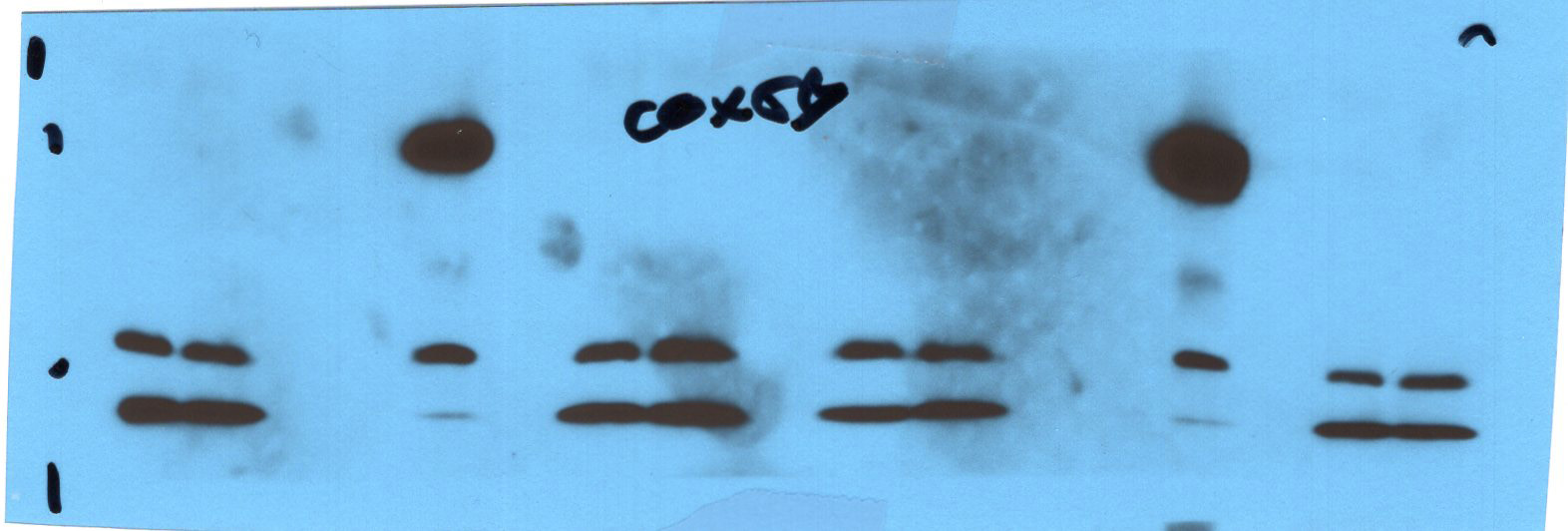

Supplement: Figure 5—figure supplement 3—source data 1. [file elife-78915-fig5-figsupp3-data1.zip › Fig 5-figure supplement 3-source data 1/Fig5s3C_cox5b.jpg]

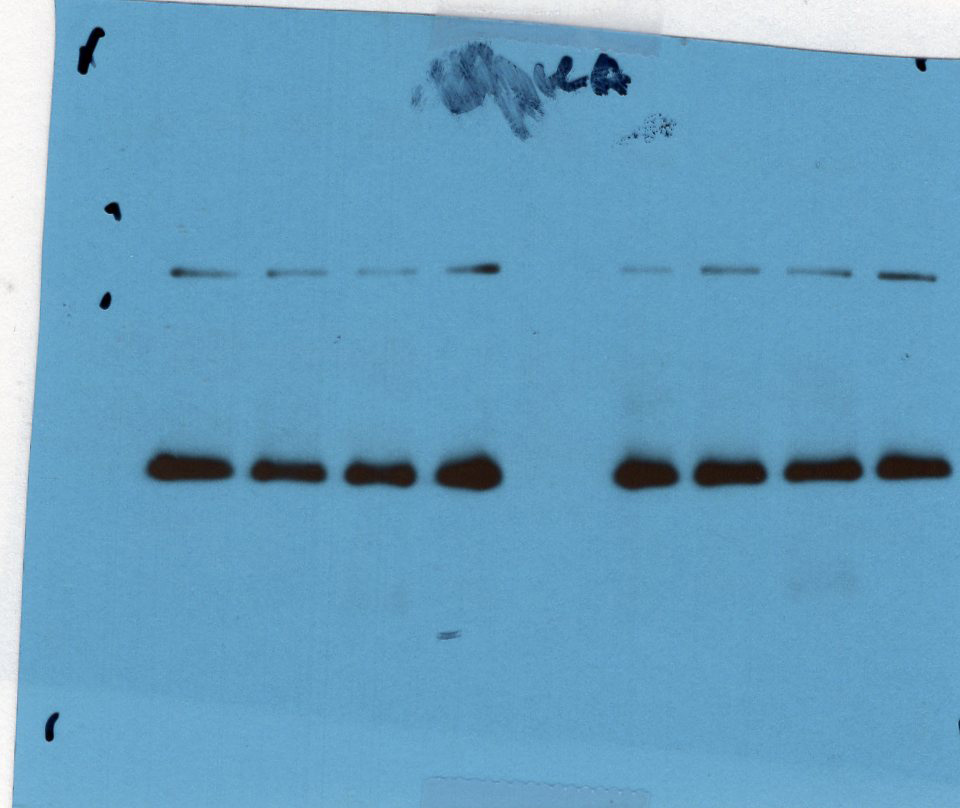

Supplement: Figure 5—figure supplement 3—source data 1. [file elife-78915-fig5-figsupp3-data1.zip › Fig 5-figure supplement 3-source data 1/Fig5s3D_DDMcore2sdha.jpg]

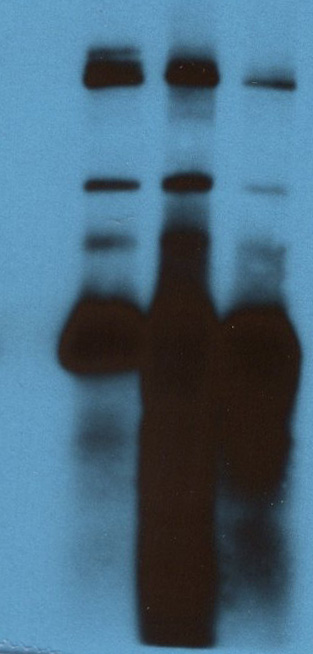

Supplement: Figure 5—figure supplement 3—source data 1. [file elife-78915-fig5-figsupp3-data1.zip › Fig 5-figure supplement 3-source data 1/Fig5s3B_cox5b.jpg]

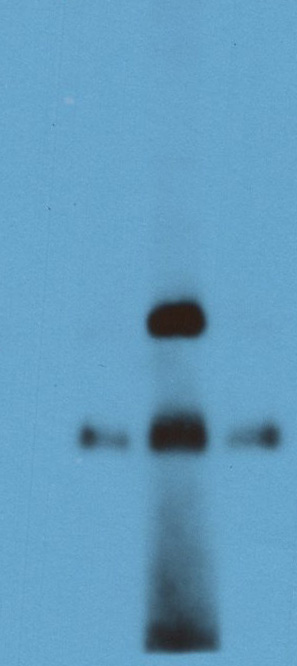

Supplement: Figure 5—figure supplement 3—source data 1. [file elife-78915-fig5-figsupp3-data1.zip › Fig 5-figure supplement 3-source data 1/Fig5s3B_sdha.jpg]

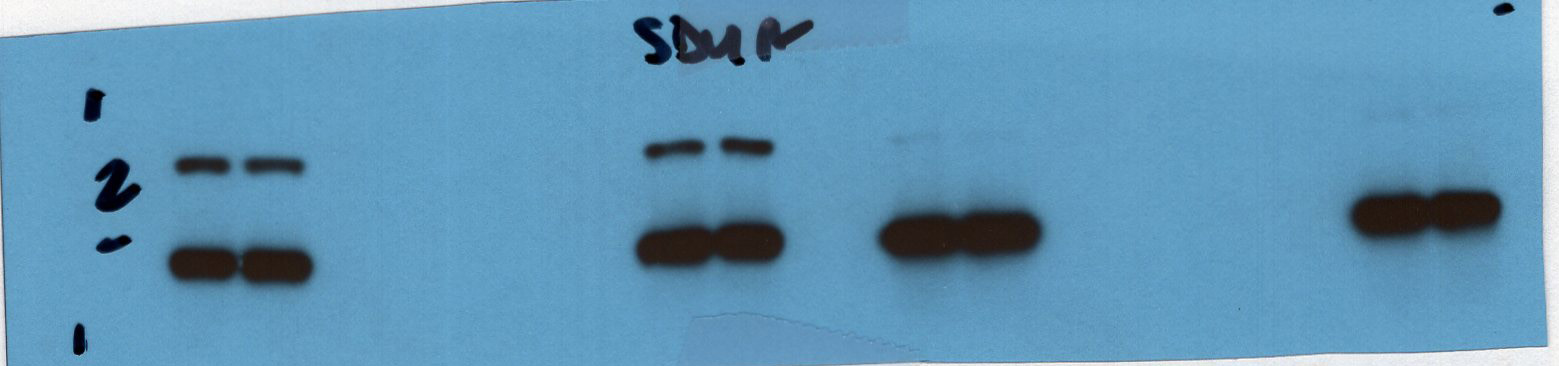

Supplement: Figure 5—figure supplement 3—source data 1. [file elife-78915-fig5-figsupp3-data1.zip › Fig 5-figure supplement 3-source data 1/Fig5s3C_sdha.jpg]

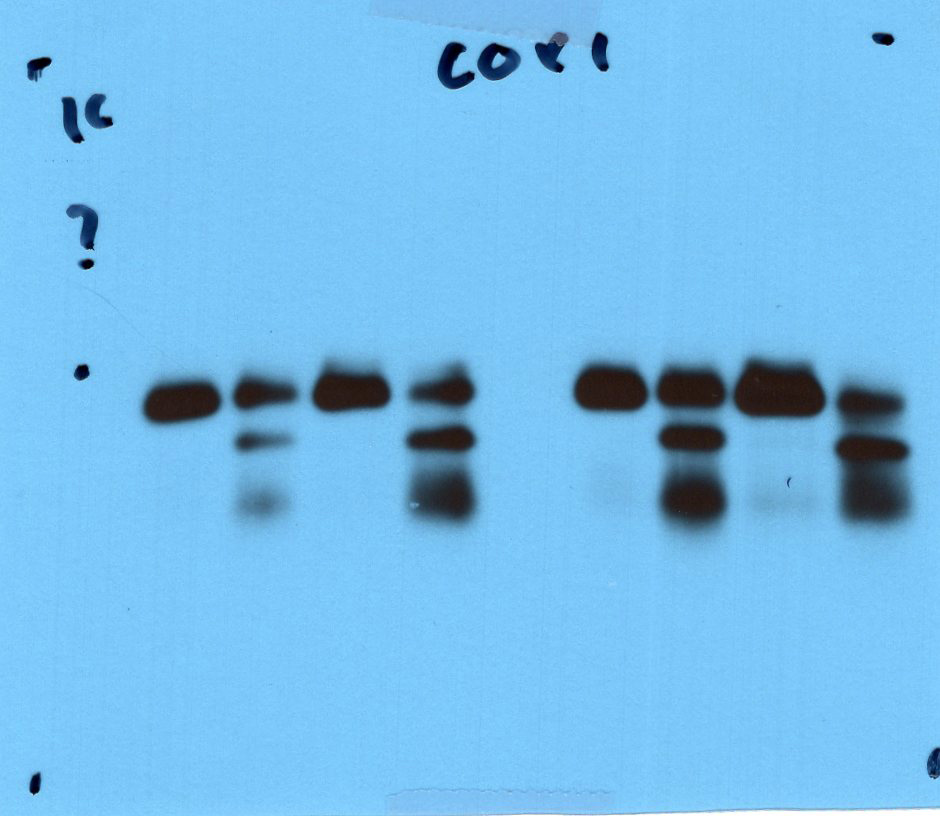

Supplement: Figure 5—figure supplement 3—source data 1. [file elife-78915-fig5-figsupp3-data1.zip › Fig 5-figure supplement 3-source data 1/Fig5s3D_DDMcox1.jpg]

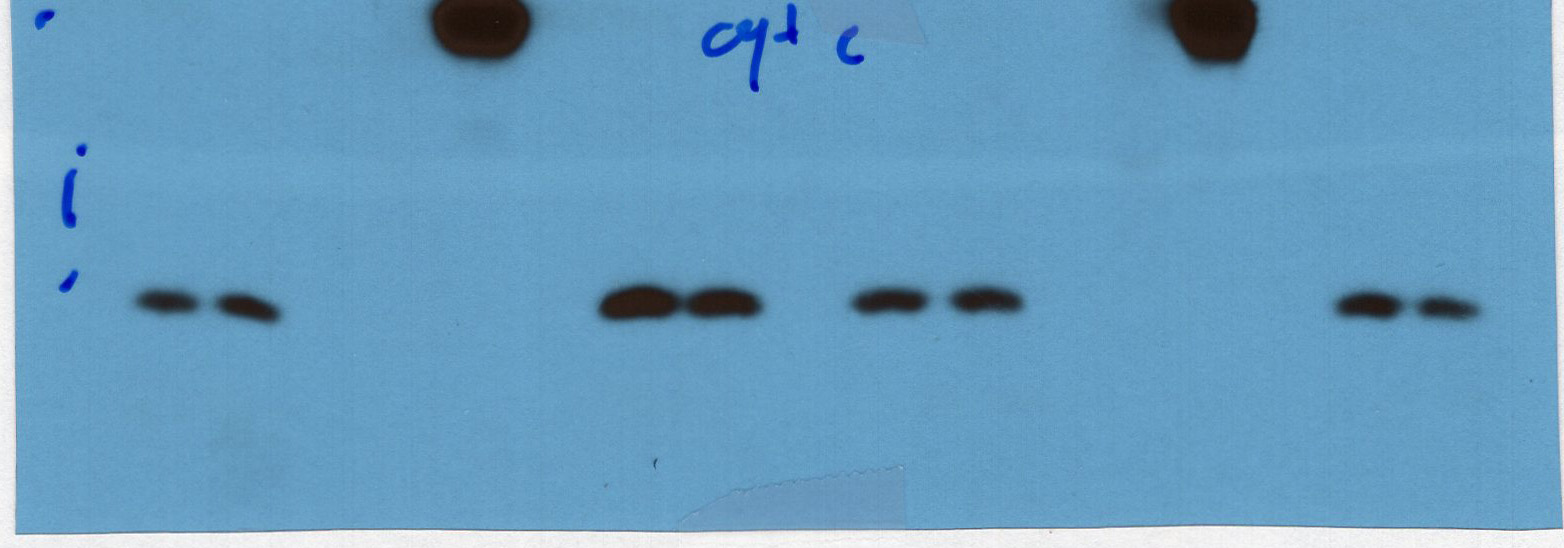

Supplement: Figure 5—figure supplement 3—source data 1. [file elife-78915-fig5-figsupp3-data1.zip › Fig 5-figure supplement 3-source data 1/Fig5s3C_cytc.jpg]

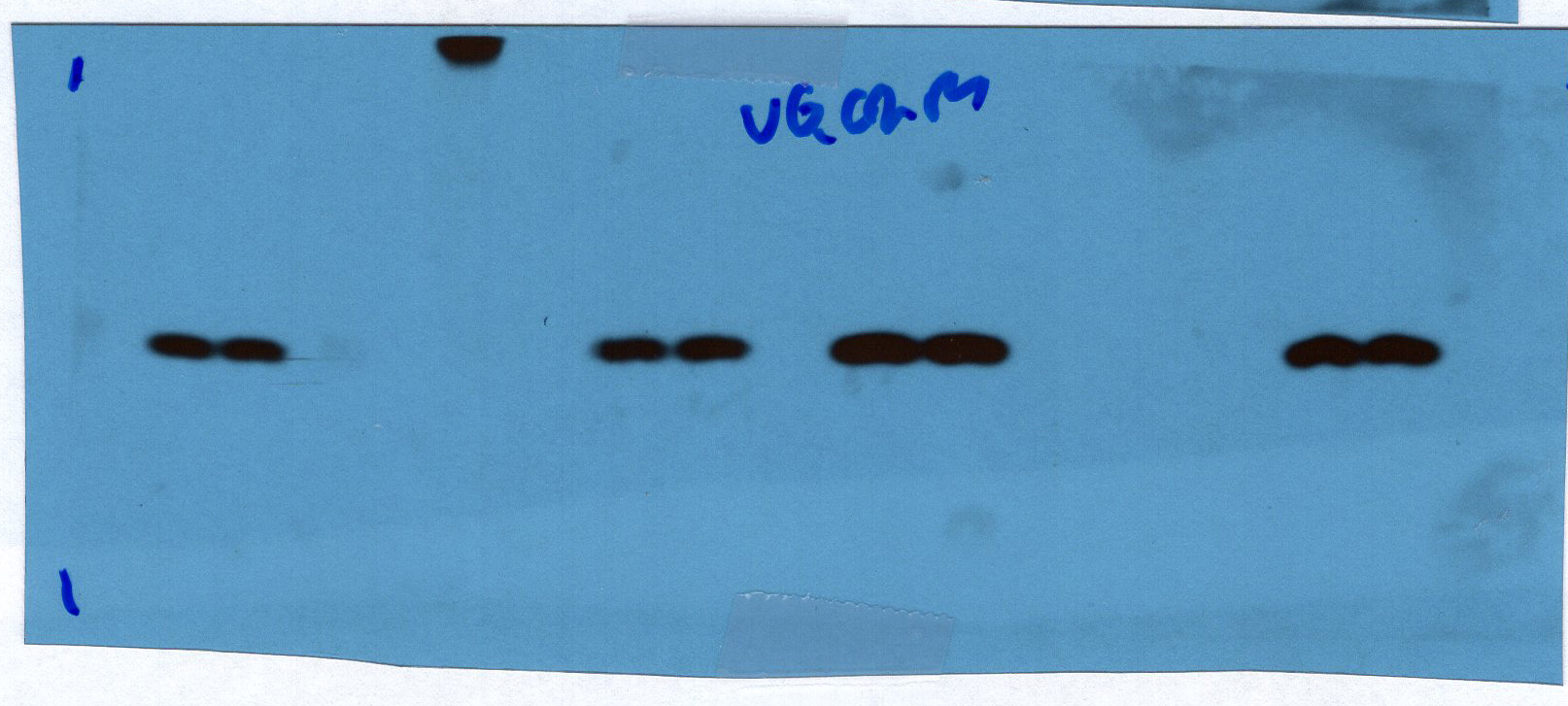

Supplement: Figure 5—figure supplement 3—source data 1. [file elife-78915-fig5-figsupp3-data1.zip › Fig 5-figure supplement 3-source data 1/Fig5s3C_uqcrb.jpg]

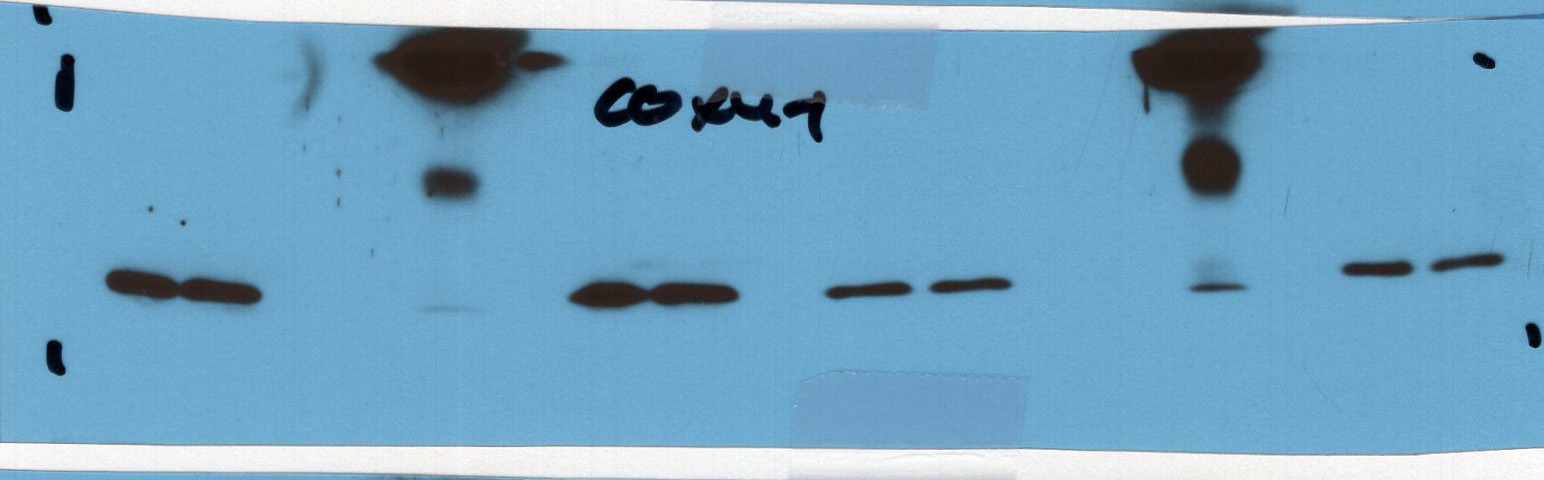

Supplement: Figure 5—figure supplement 3—source data 1. [file elife-78915-fig5-figsupp3-data1.zip › Fig 5-figure supplement 3-source data 1/Fig5s3C_cox4i1.jpg]

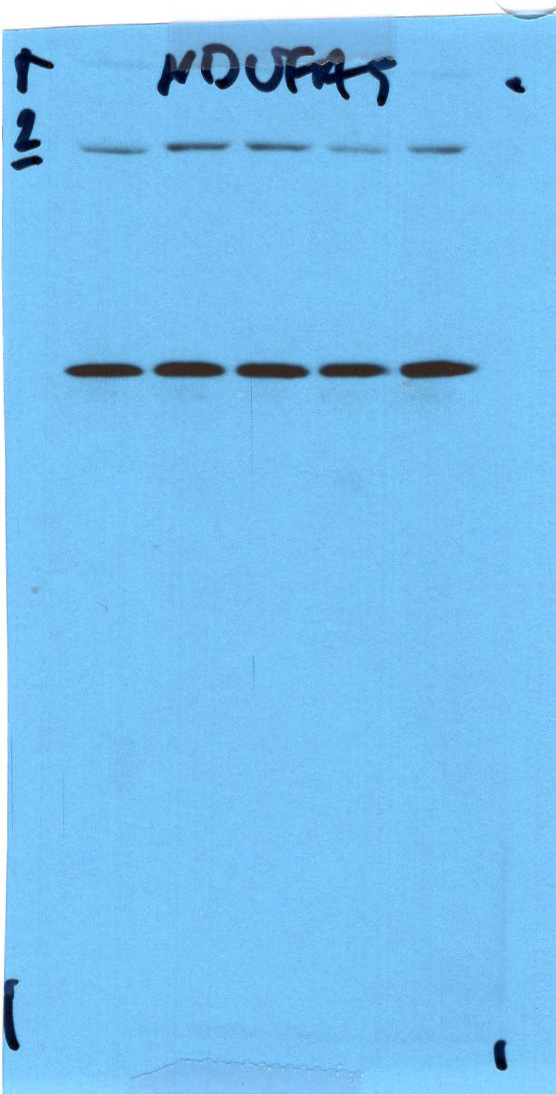

Supplement: Figure 5—figure supplement 3—source data 1. [file elife-78915-fig5-figsupp3-data1.zip › Fig 5-figure supplement 3-source data 1/Fig5s3A_ndufa9.jpg]

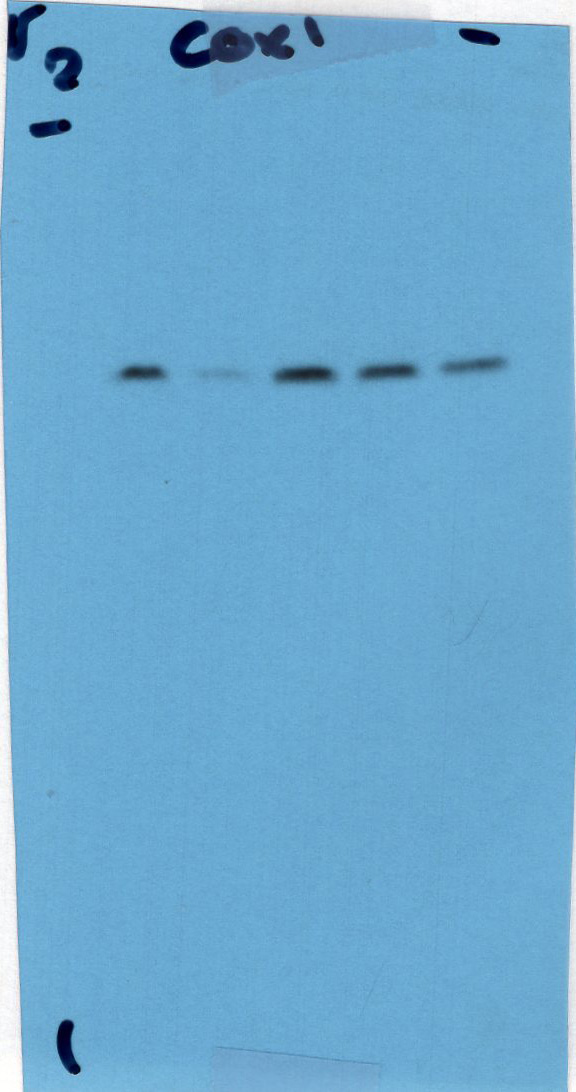

Supplement: Figure 5—figure supplement 3—source data 1. [file elife-78915-fig5-figsupp3-data1.zip › Fig 5-figure supplement 3-source data 1/Fig5s3A_cox1.jpg]

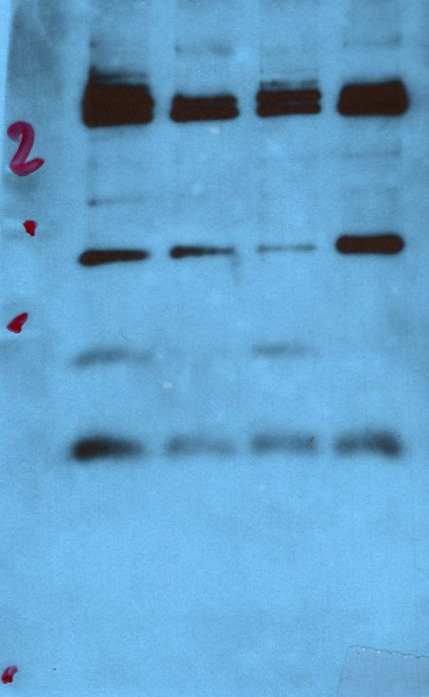

Supplement: Figure 5—figure supplement 3—source data 1. [file elife-78915-fig5-figsupp3-data1.zip › Fig 5-figure supplement 3-source data 1/Fig5s3D_Digsdha2.jpg]

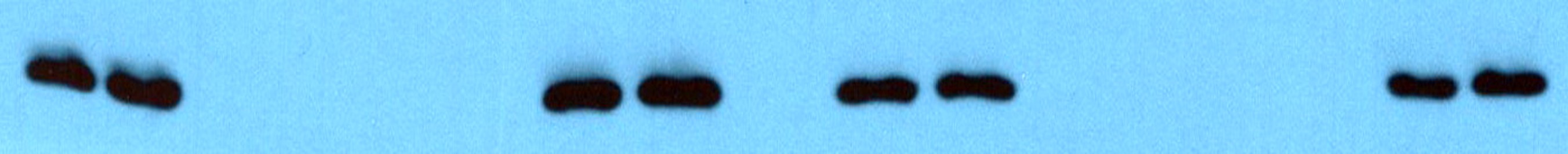

Supplement: Figure 5—figure supplement 3—source data 1. [file elife-78915-fig5-figsupp3-data1.zip › Fig 5-figure supplement 3-source data 1/Fig5s3C_core2.jpg]

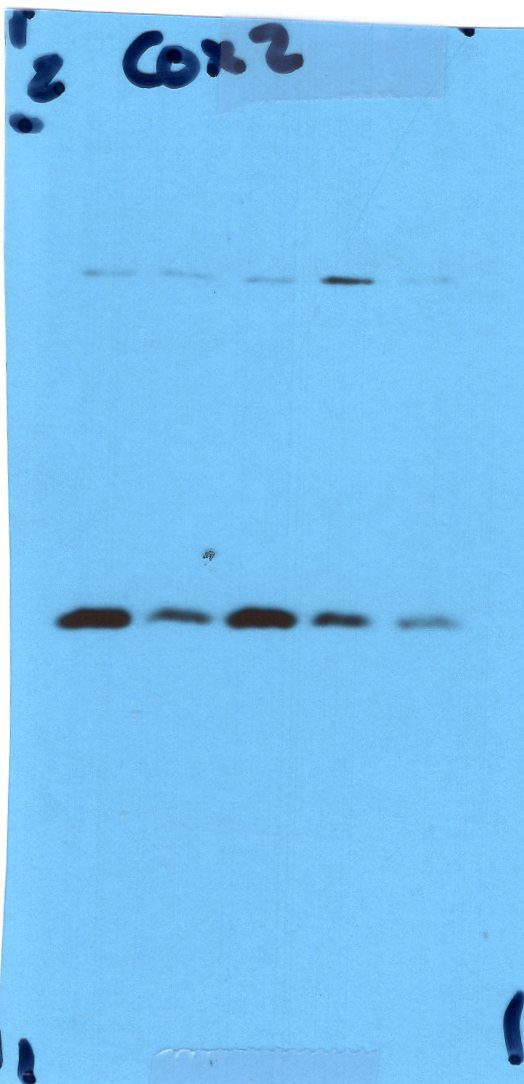

Supplement: Figure 5—figure supplement 3—source data 1. [file elife-78915-fig5-figsupp3-data1.zip › Fig 5-figure supplement 3-source data 1/Fig5s3A_cox2.jpg]

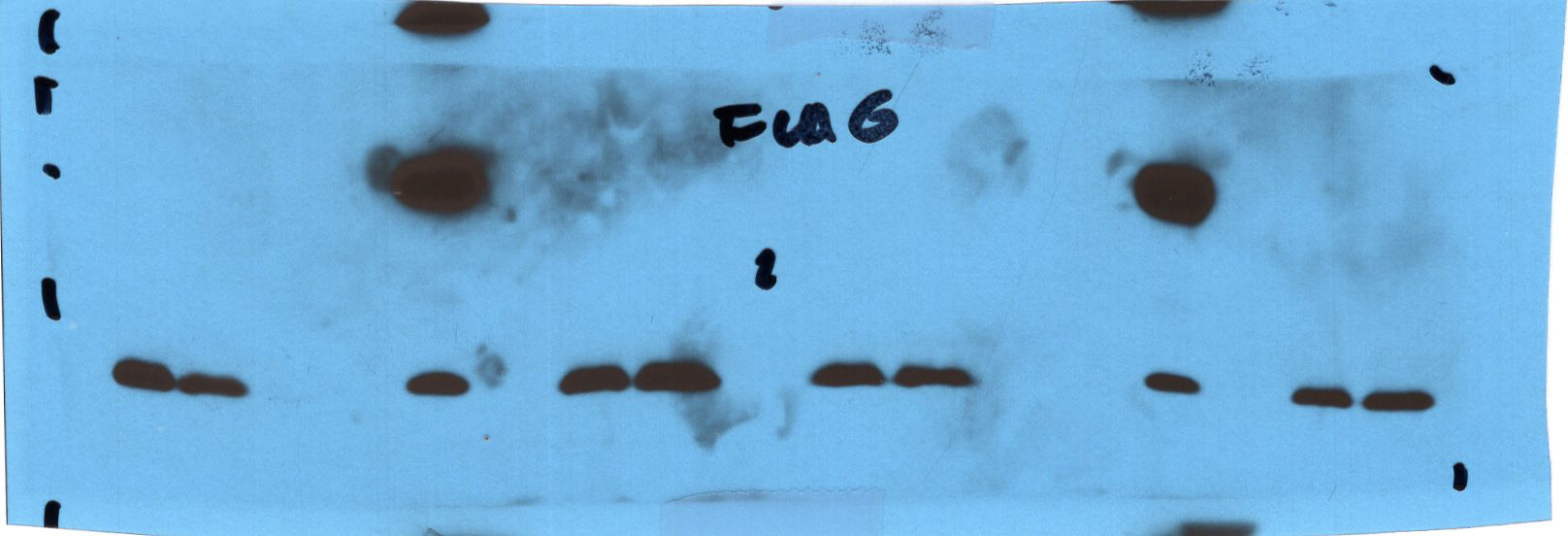

Supplement: Figure 5—figure supplement 3—source data 1. [file elife-78915-fig5-figsupp3-data1.zip › Fig 5-figure supplement 3-source data 1/Fig5s3C_flag.jpg]

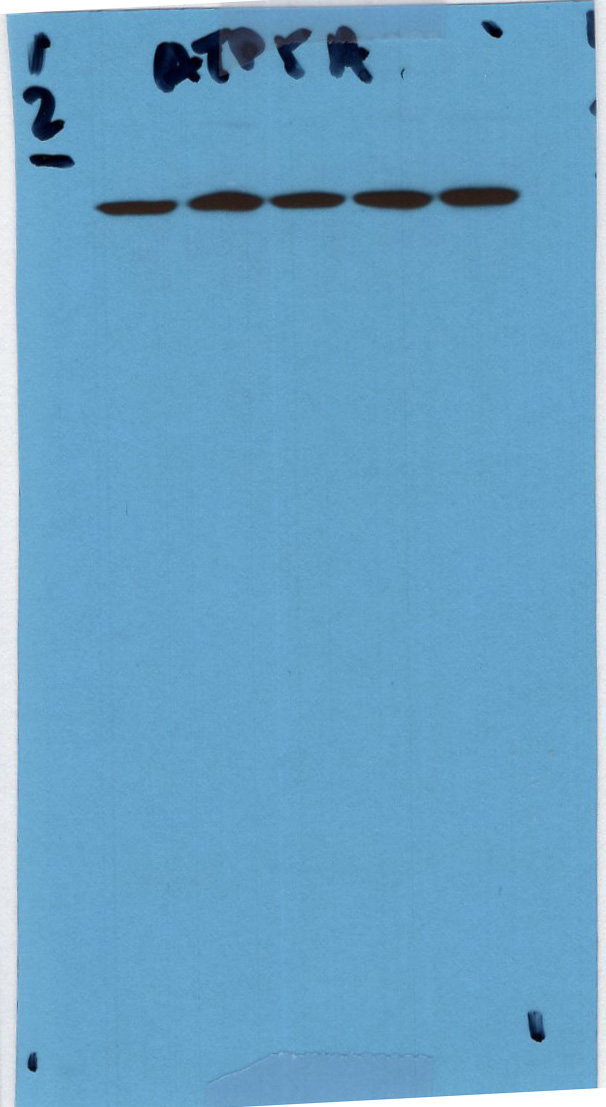

Supplement: Figure 5—figure supplement 3—source data 1. [file elife-78915-fig5-figsupp3-data1.zip › Fig 5-figure supplement 3-source data 1/Fig5s3A_atp5a.jpg]

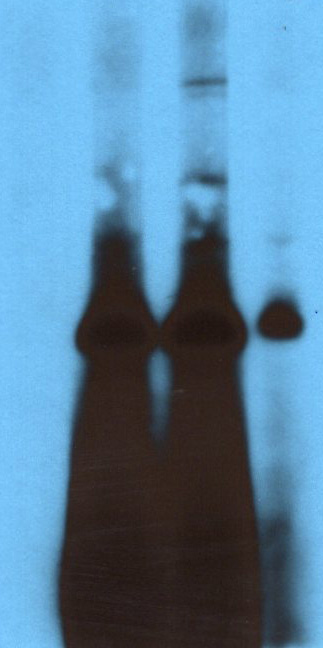

Supplement: Figure 5—figure supplement 3—source data 1. [file elife-78915-fig5-figsupp3-data1.zip › Fig 5-figure supplement 3-source data 1/Fig5s3B_flag.jpg]

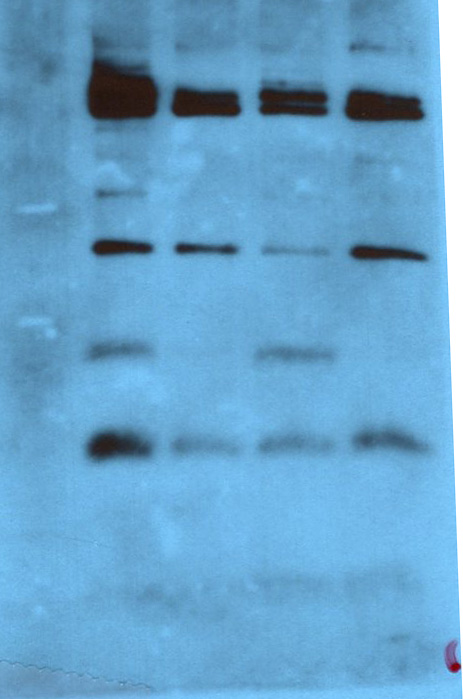

Supplement: Figure 5—figure supplement 3—source data 1. [file elife-78915-fig5-figsupp3-data1.zip › Fig 5-figure supplement 3-source data 1/Fig5s3D_Digsdha1.jpg]

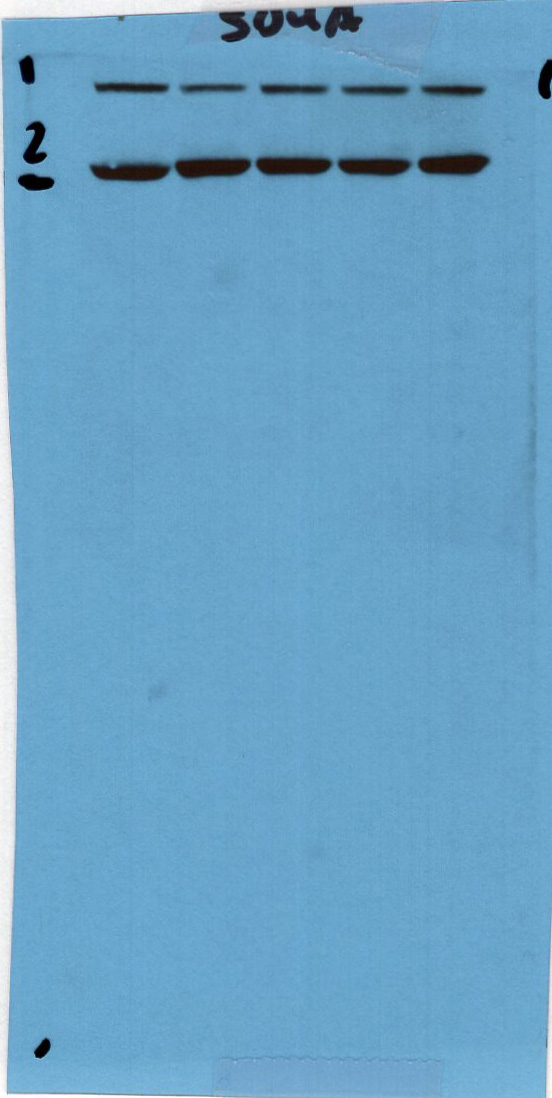

Supplement: Figure 5—figure supplement 3—source data 1. [file elife-78915-fig5-figsupp3-data1.zip › Fig 5-figure supplement 3-source data 1/Fig5s3A_sdha.jpg]

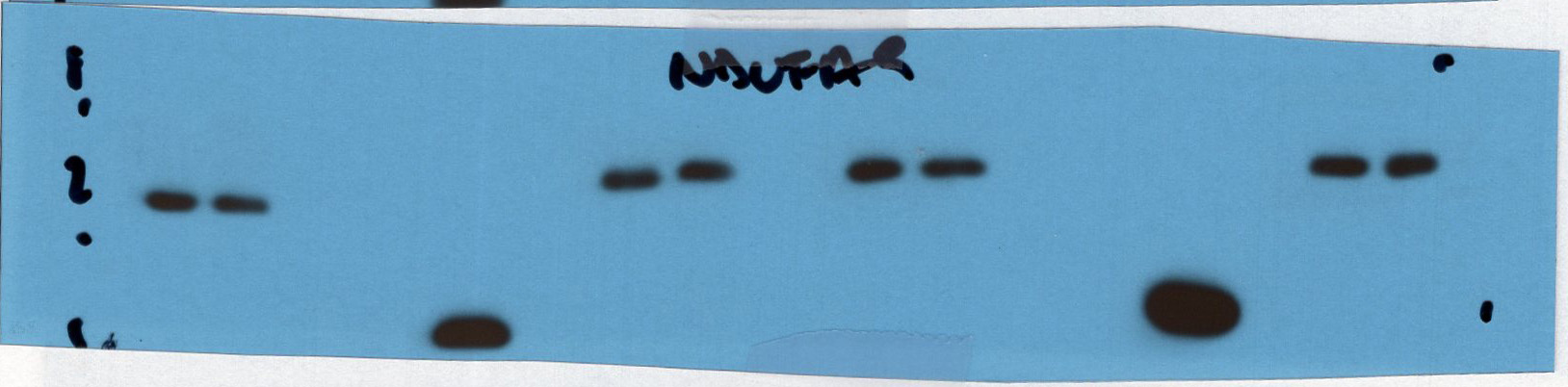

Supplement: Figure 5—figure supplement 3—source data 1. [file elife-78915-fig5-figsupp3-data1.zip › Fig 5-figure supplement 3-source data 1/Fig5s3C_ndufa9.jpg]
