# Supplementary material for: Tissue-specific mitochondrial HIGD1C promotes oxygen sensitivity in carotid body chemoreceptors
Source: eLife. 2022 Oct 18;11:e78915. doi: 10.7554/eLife.78915 (PMC9635879; doi:10.7554/eLife.78915)
Supplement: Figure 5—source data 1. [file elife-78915-fig5-data1.zip › Fig 5-source data 1/Fig 5-source data 1.pdf]

Figure 5 – panel A

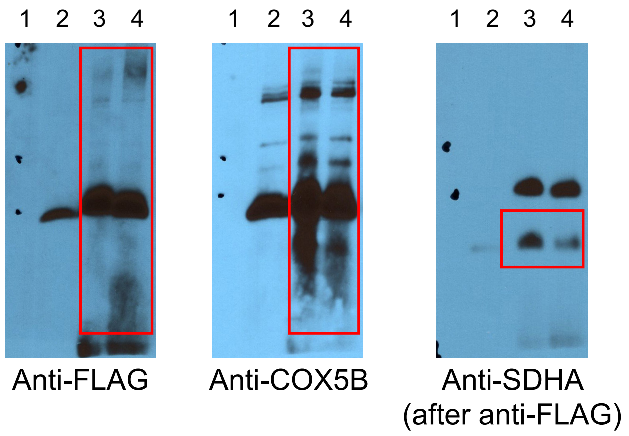

1. Ladder (apoferritin 450/900kDa)
2. HIGD1A-KO+HIGD1C (75μg protein)
3. HIGD1A-KO+HIGD1C (150μg protein)
4. HIGD1A-KO+Higd1c (150μg protein)

Figure 5 – panel B

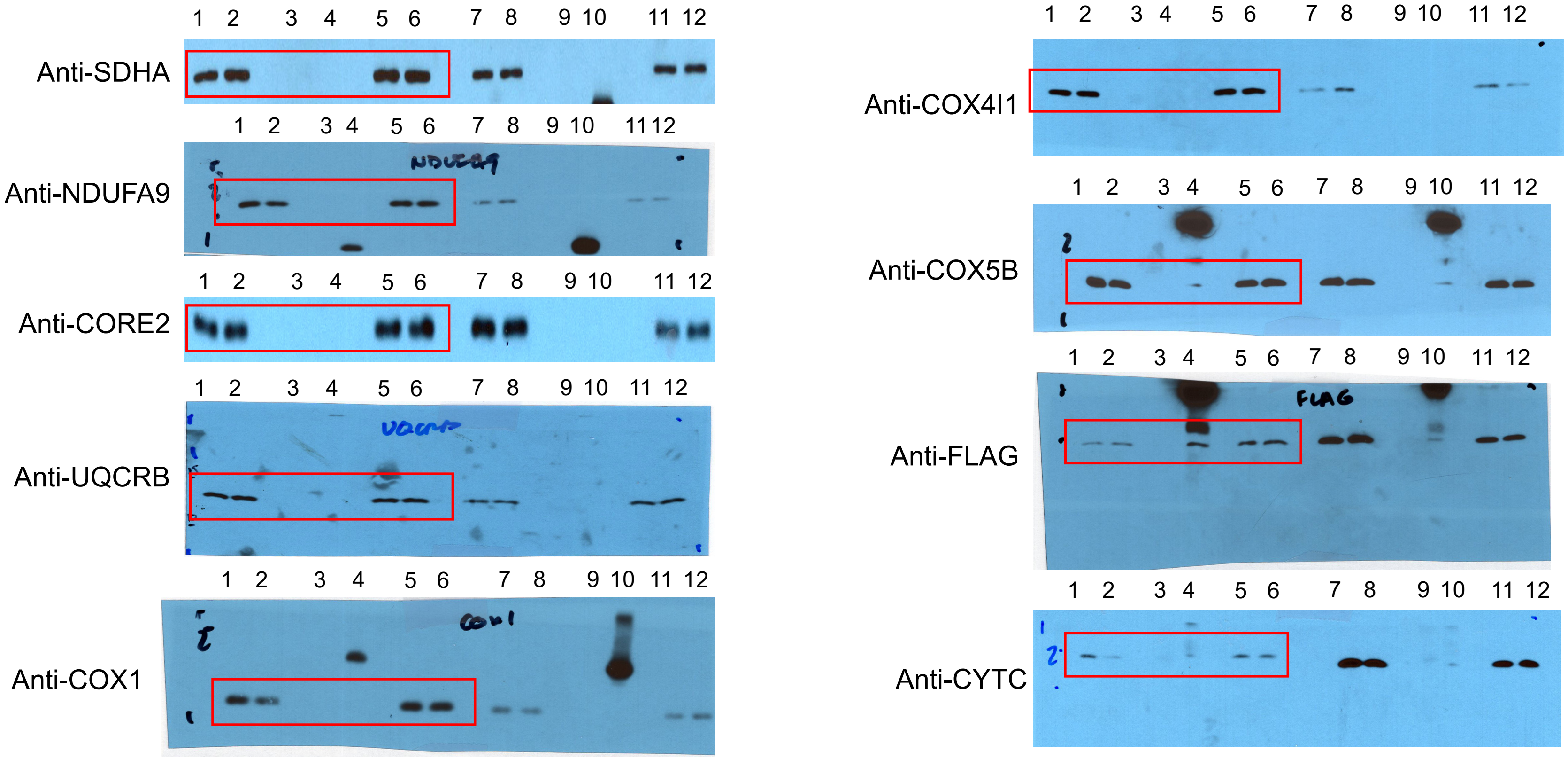

1. Extract control
2. Unbound control
3. IP control
4. IP FLAG
5. Unbound FLAG
6. Extract FLAG

HIGD1A-KO +HIGD1C-FLAG

7. Extract control
8. Unbound control
9. IP control
10. IP FLAG
11. Unbound FLAG
12. Extract FLAG

Figure5-figure supplement 2 panel D

Figure 5 – panel C

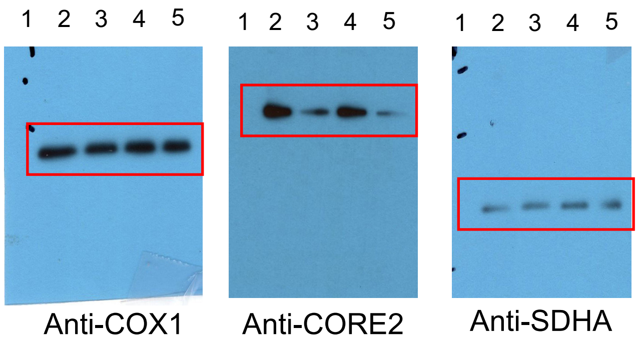

DDM

1. Ladder (apoferritin 450/900kDa)
2. WT
3. HIGD1A-KO+EV
4. HIGD1A-KO+HIGD1A
5. HIGD1A-KO+HIGD1C

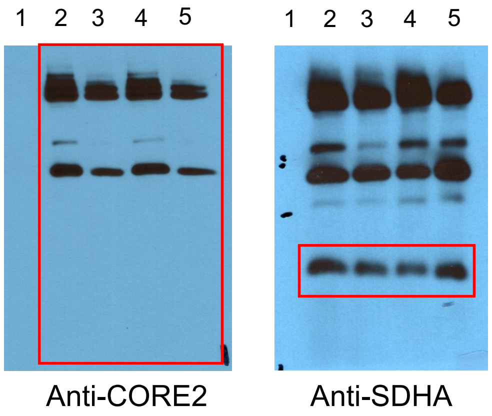

DIGITONIN

1. Ladder (apoferritin 450/900kDa)
2. WT
3. HIGD1A-KO+EV
4. HIGD1A-KO+HIGD1A
5. HIGD1A-KO+HIGD1C
