# Supplementary material for: Tissue-specific mitochondrial HIGD1C promotes oxygen sensitivity in carotid body chemoreceptors
Source: eLife. 2022 Oct 18;11:e78915. doi: 10.7554/eLife.78915 (PMC9635879; doi:10.7554/eLife.78915)
Supplement: Figure 5—figure supplement 1—source data 1. [file elife-78915-fig5-figsupp1-data1.zip › Fig 5-figure supplement 1-source data 1/Fig 5-figure supplement 1-source data 1.pdf]

Figure 5 figure supplement 1– panel A

1. Ladder
2. WT+EV
3. WT+COX4I1
4. WT+COX4I2
5. WT+HIGD1C
6. WT+HIGD1C+COX4I1
7. WT+HIGD1C+COX4I2
8. WT+Higd1c
9. WT+Higd1c+COX4I1
10. WT+Higd1c+COX4I2

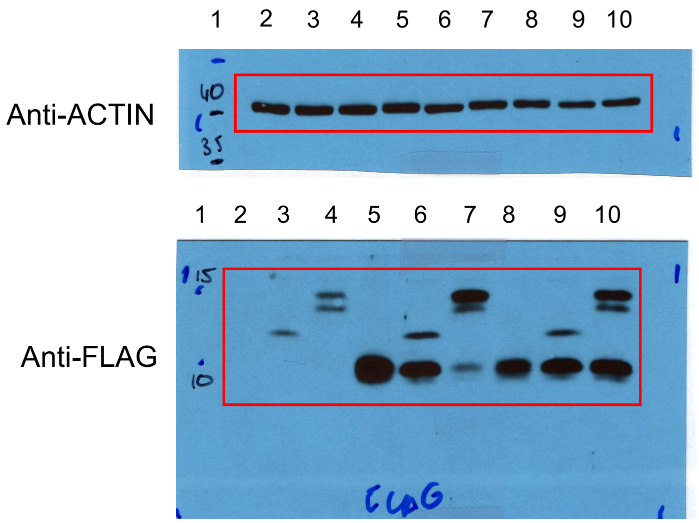

Figure 5 figure supplement 1– panel B

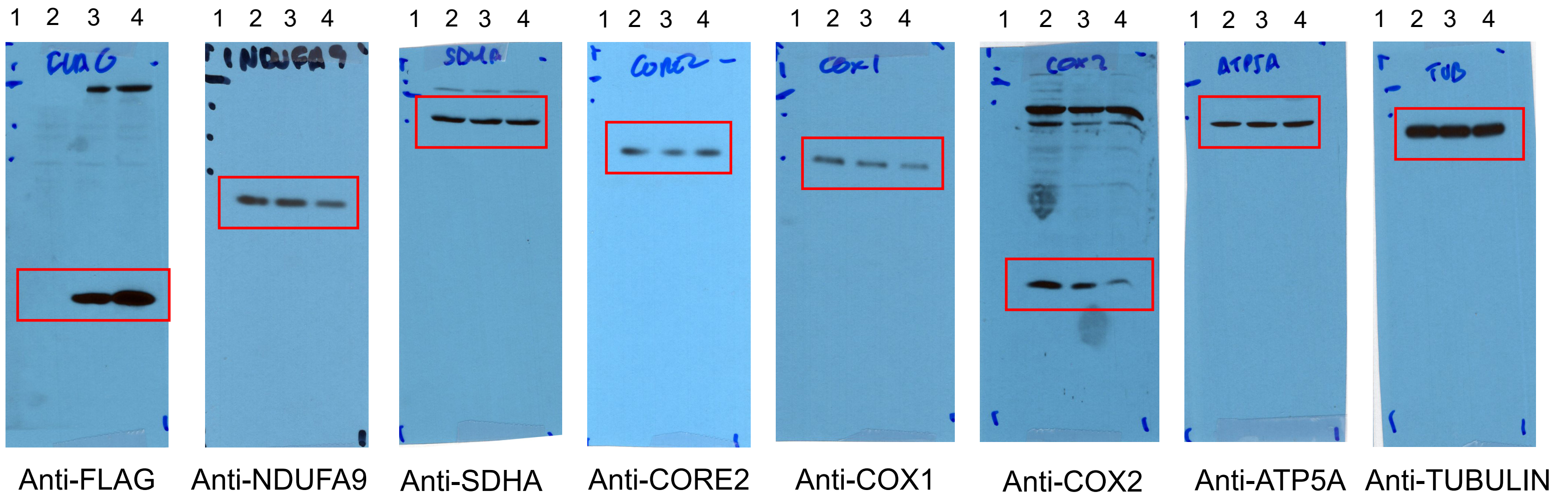

1. Ladder
2. WT+EV
3. WT+HIGD1C
4. WT+Higd1c

Figure 5 figure supplement 1– panel C

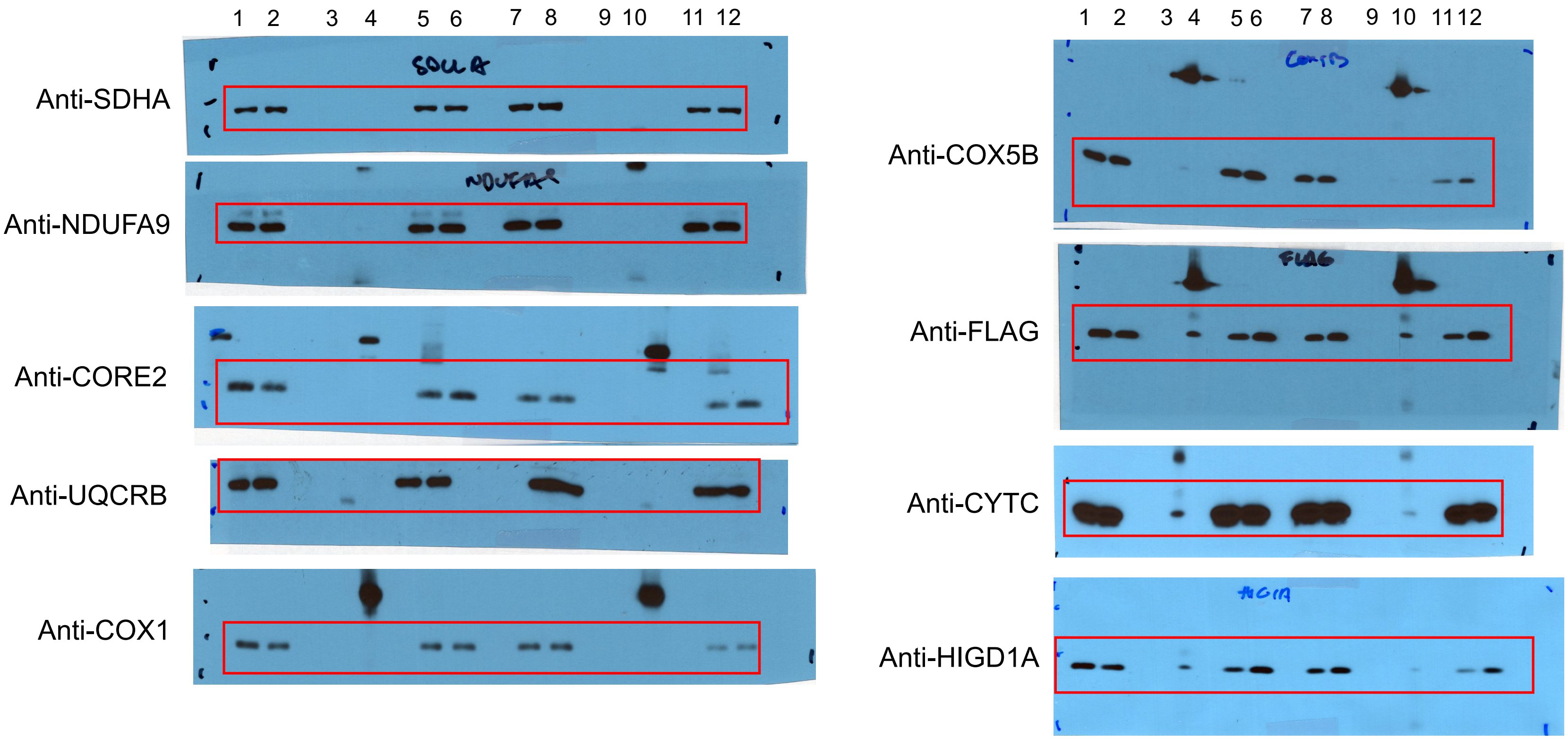

1. Extract control
2. Unbound control
3. IP control
4. IP FLAG
5. Unbound FLAG
6. Extract FLAG

HEK293T +HIGD1C-FLAG

7. Extract control
8. Unbound control
9. IP control
10. IP FLAG
11. Unbound FLAG
12. Extract FLAG

HEK293T +Higd1c-FLAG

Figure 5 figure supplement 1– panel D

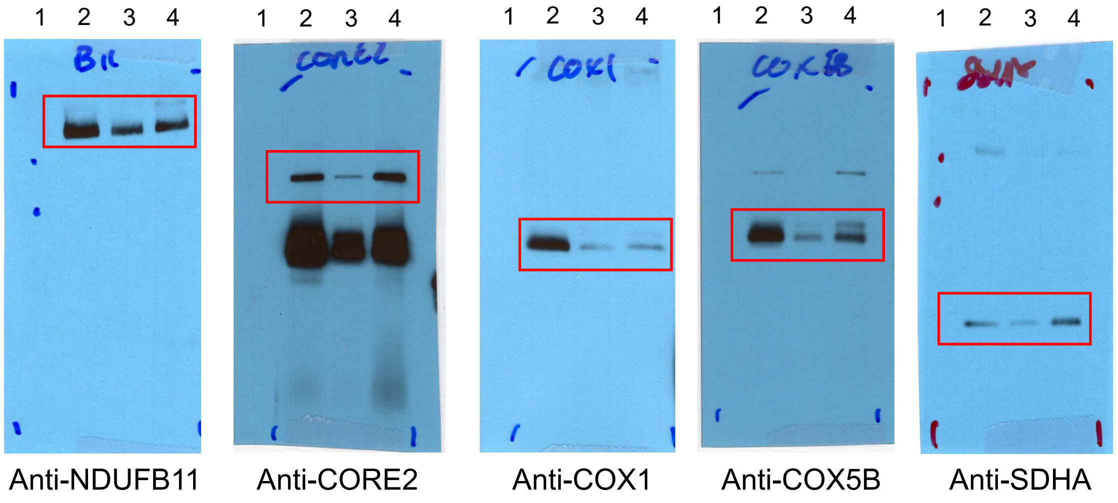

1. Ladder apoferritin
2. WT+EV
3. WT+HIGD1C
4. WT+Higd1c

Figure 5 figure supplement 1– panel E

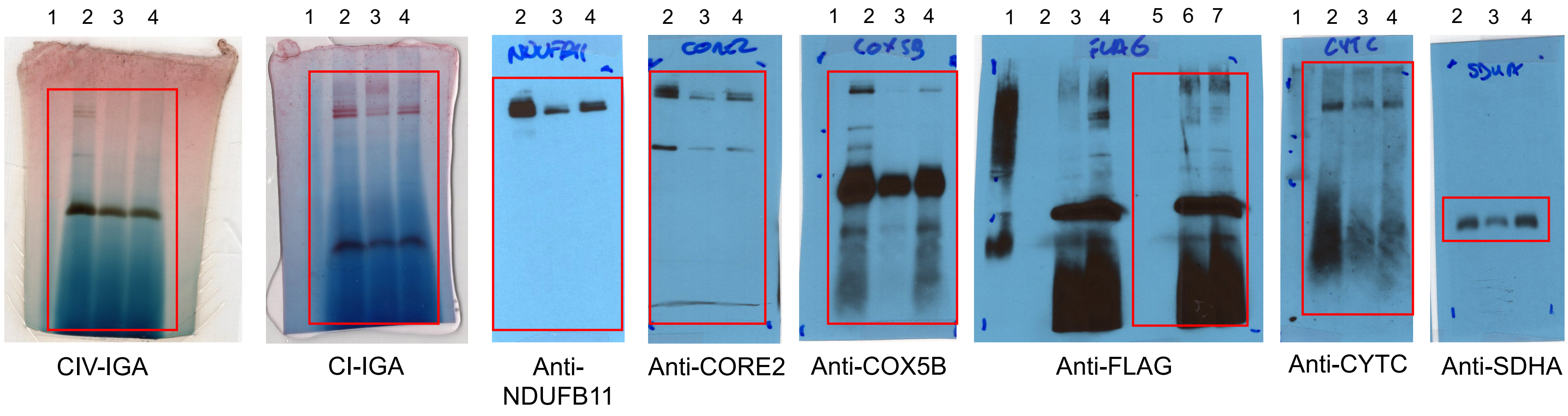

1. Ladder apoferritin
2. WT+EV
3. WT+HIGD1C
4. WT+Higd1c
5. WT+EV
6. WT+HIGD1C
7. WT+Higd1c

(5–7: 2nd rep)
