# Supplementary material for: Tissue-specific mitochondrial HIGD1C promotes oxygen sensitivity in carotid body chemoreceptors
Source: eLife. 2022 Oct 18;11:e78915. doi: 10.7554/eLife.78915 (PMC9635879; doi:10.7554/eLife.78915)
Supplement: Figure 5—figure supplement 2—source data 1. [file elife-78915-fig5-figsupp2-data1.zip › Fig 5-figure supplement 2-source data 1/Fig 5-figure supplement 2-source data 1.pdf]

Figure 5 figure supplement 2– panel A

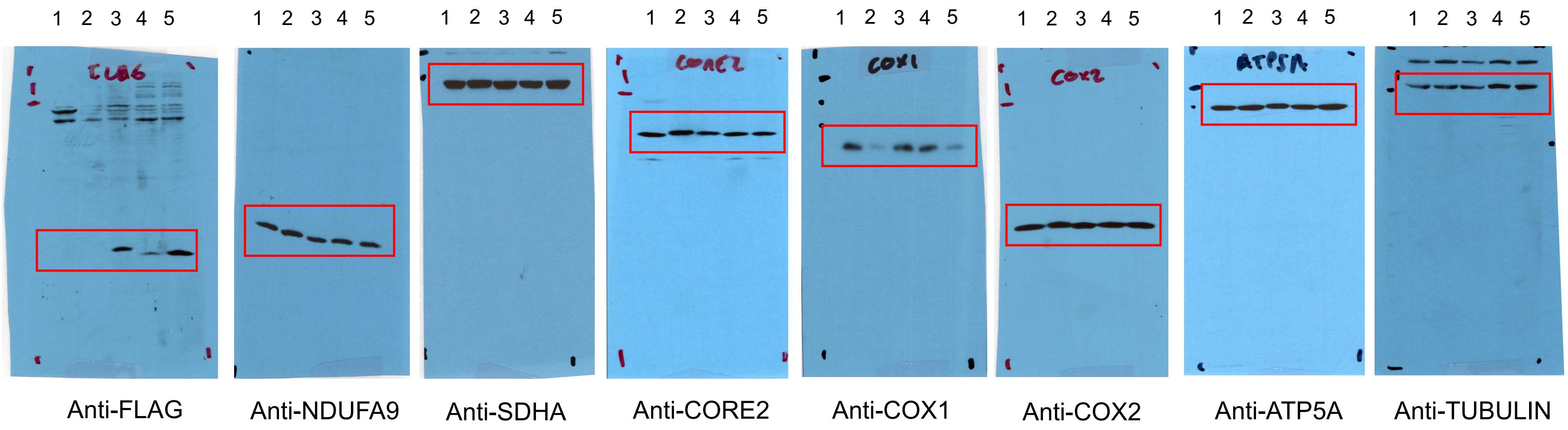

1. WT
2. HIGD1A-KO+EV
3. HIGD1A-KO+HIGD1A
4. HIGD1A-KO+HIGD1C
5. HIGD1A-KO+Higd1c

Figure 5 figure supplement 2– panel B

1. WT
2. HIGD1A-KO+EV
3. HIGD1A-KO+HIGD1A
4. HIGD1A-KO+COX4I1
5. HIGD1A-KO+COX4I2
6. HIGD1A-KO+HIGD1C
7. HIGD1A-KO+HIGD1C+COX4I1
8. HIGD1A-KO+HIGD1C+COX4I2
9. HIGD1A-KO+Higd1c
10. HIGD1A-KO+Higd1c+COX4I1
11. HIGD1A-KO+Higd1c+COX4I2

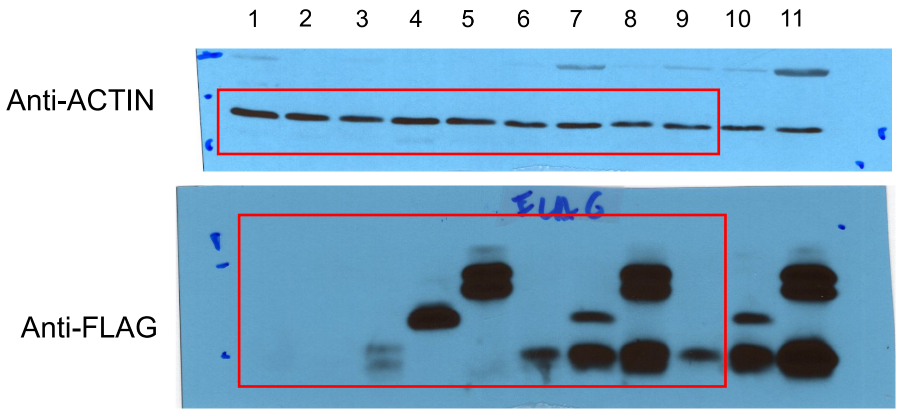

Figure 5 figure supplement 2– panel C

1. WT
2. HIGD1A-KO+EV
3. HIGD1A-KO+HIGD1A
4. HIGD1A-KO+HIGD1C
5. HIGD1A-KO+HIGD1C+COX4I2
6. HIGD1A-KO+HIGD1C+COX4I2+siCOX4I1

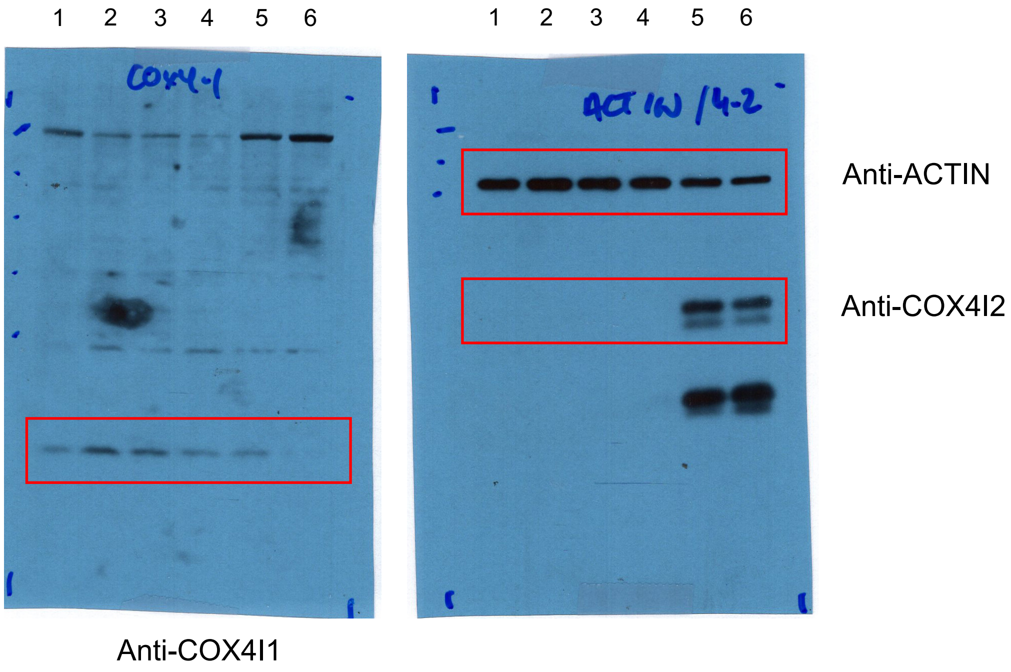

Figure 5 figure supplement 2– panel D

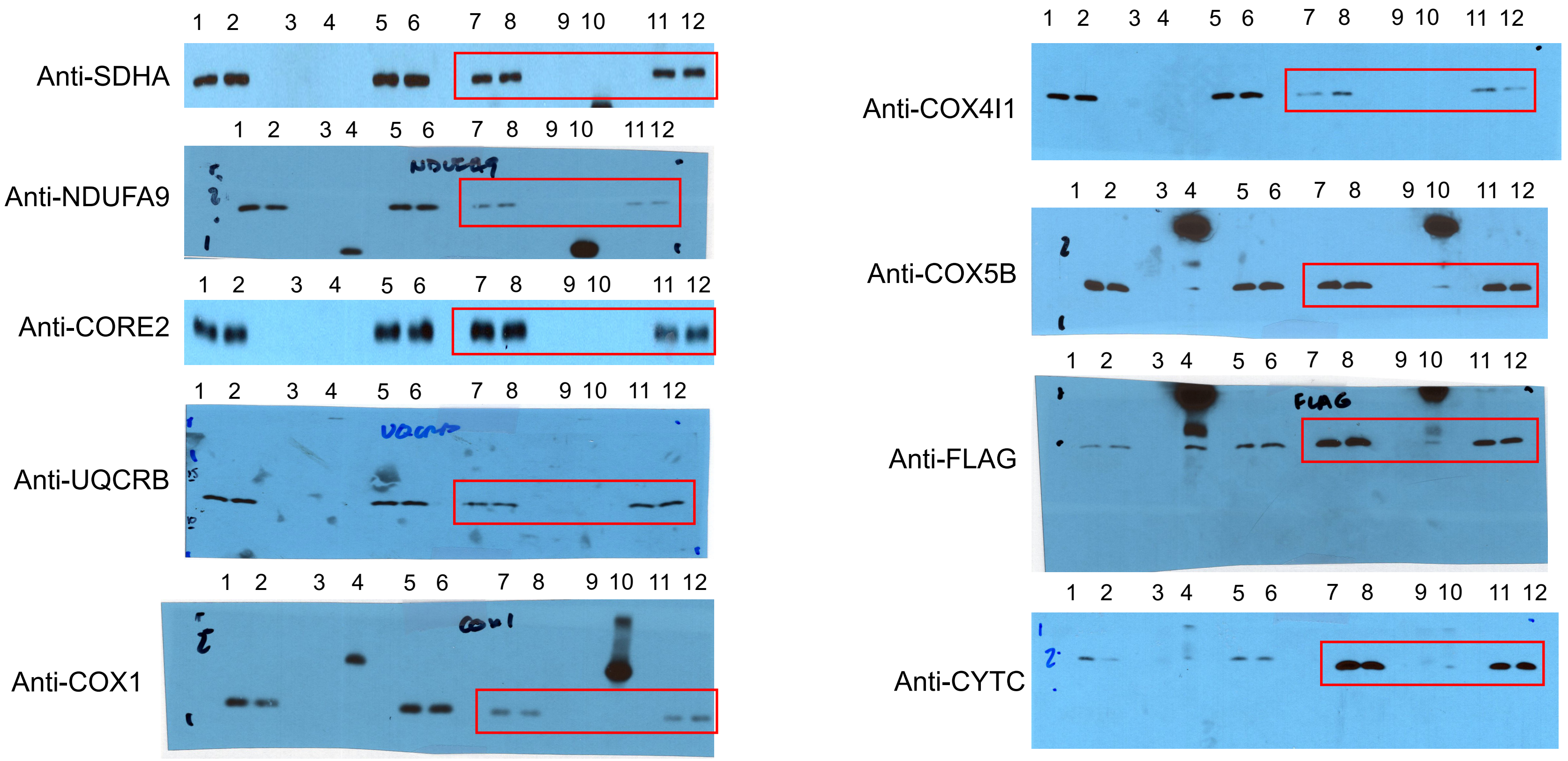

1. Extract control
2. Unbound control
3. IP control
4. IP FLAG
5. Unbound FLAG
6. Extract FLAG

Figure5-panel B

7. Extract control
8. Unbound control
9. IP control
10. IP FLAG
11. Unbound FLAG
12. Extract FLAG

HIGD1A-KO
+Higd1c-FLAG

Figure 5 figure supplement 2– panel E

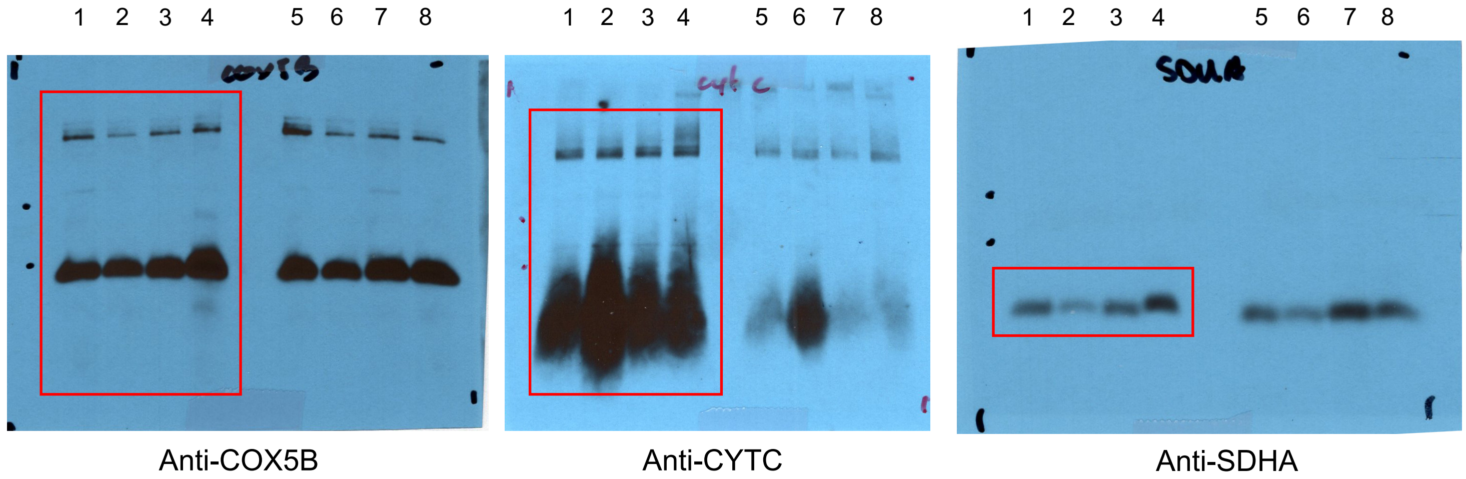

1. WT
2. HIGD1A-KO+EV
3. HIGD1A-KO+HIGD1A
4. HIGD1A-KO+HIGD1C
5. WT
6. HIGD1A-KO+EV
7. HIGD1A-KO+HIGD1A
8. HIGD1A-KO+HIGD1C

(Lanes 5–8:) 2nd rep
