# Supplementary material for: Tissue-specific mitochondrial HIGD1C promotes oxygen sensitivity in carotid body chemoreceptors
Source: eLife. 2022 Oct 18;11:e78915. doi: 10.7554/eLife.78915 (PMC9635879; doi:10.7554/eLife.78915)
Supplement: Figure 5—figure supplement 3—source data 1. [file elife-78915-fig5-figsupp3-data1.zip › Fig 5-figure supplement 3-source data 1/Fig 5-figure supplement 3-source data 1.pdf]

Figure 5 - figure supplement 3– panel A

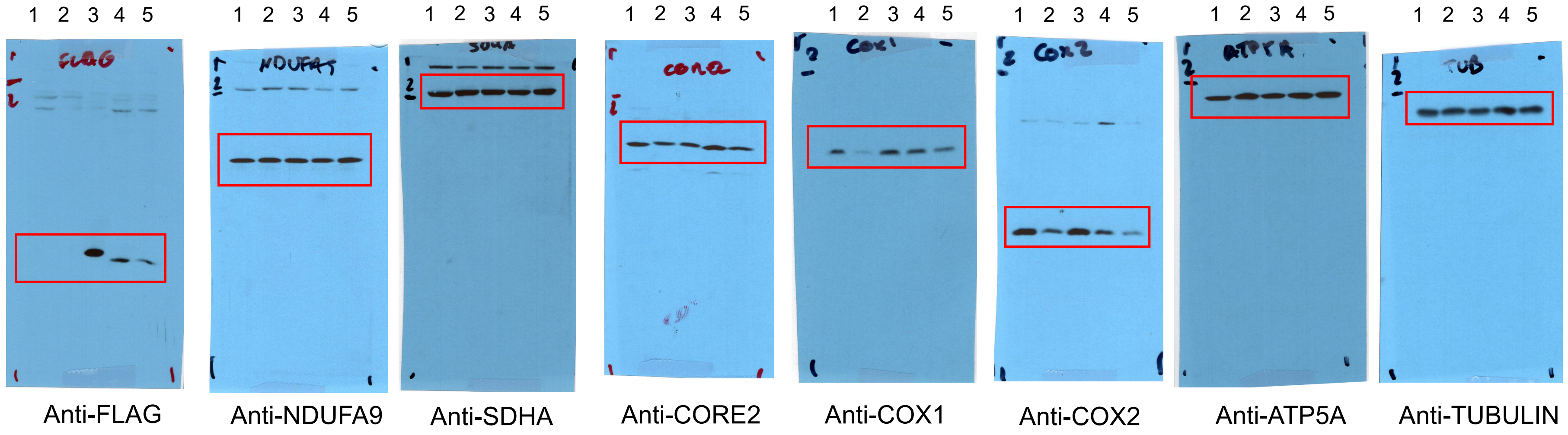

1. WT
2. HIGD2A-KO+EV
3. HIGD2A-KO+HIGD2A
4. HIGD2A-KO+HIGD1C
5. HIGD2A-KO+Higd1c

Figure 5 – figure supplementary 3 - panel B

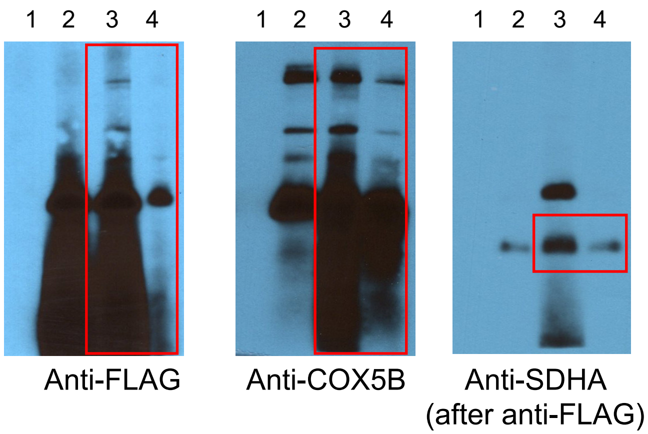

1. Ladder (apoferritin)
2. HIGD2A-KO+HIGD2A
3. HIGD2A-KO+HIGD1C
4. HIGD2A-KO+Higd1c

Figure 5 – figure supplementary 3 - panel C

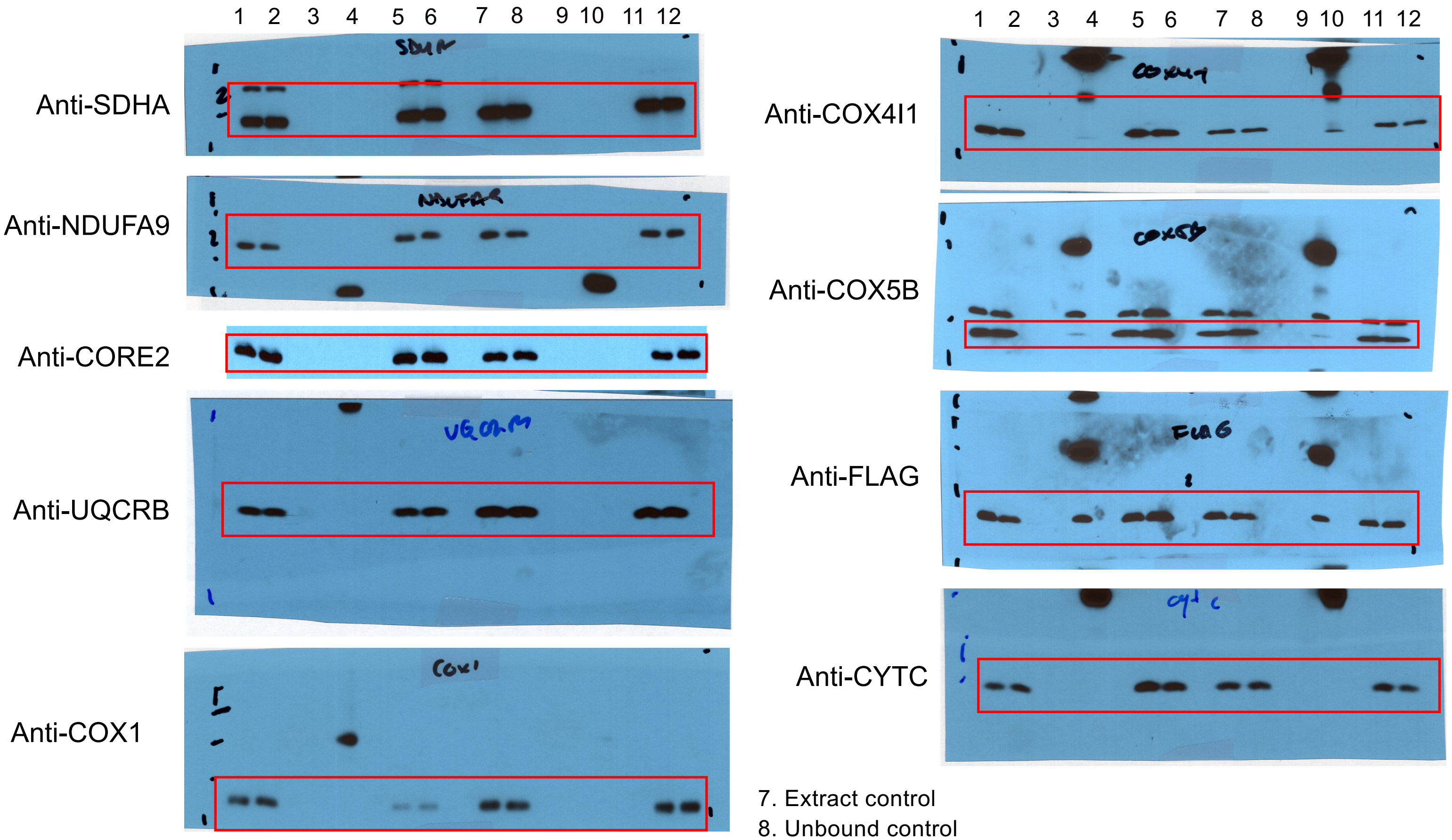

1. Extract control
2. Unbound control
3. IP control
4. IP FLAG
5. Unbound FLAG
6. Extract FLAG

HIGD2A-KO
+Higd1c-FLAG

7. Extract control
8. Unbound control
9. IP control
10. IP FLAG
11. Unbound FLAG
12. Extract FLAG

HIGD2A-KO
+Higd1c-FLAG

Figure 5 – figure supplementary 3 - panel D

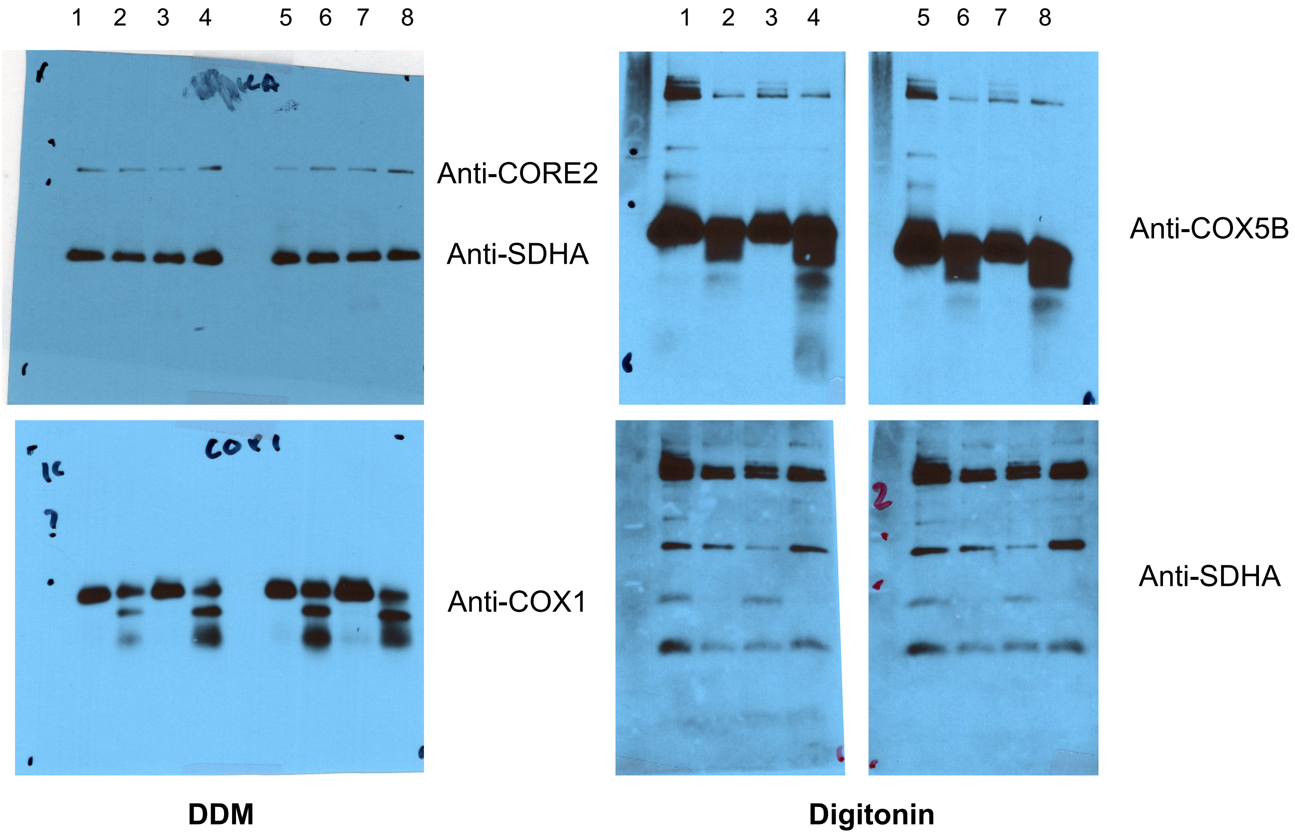

1. WT
2. HIGD2A-KO+EV
3. HIGD2A-KO+HIGD2A
4. HIGD2A-KO+HIGD1C
5. WT
6. HIGD2A-KO+EV
7. HIGD2A-KO+HIGD2A
8. HIGD2A-KO+Higd1c
